# Multiscale Molecular Simulations of Polymer-Matrix Nanocomposites or What Molecular Simulations Have Taught us About the Fascinating Nanoworld

**DOI:** 10.1007/s11831-016-9207-y

**Published:** 2017-02-22

**Authors:** Georgios G. Vogiatzis, Doros N. Theodorou

**Affiliations:** 1 School of Chemical Engineering, National Technical University of Athens, 9 Heroon Polytechniou Street, Zografou Campus, 15780 Athens, Greece; 2 Present Address: Department of Mechanical Engineering, Eindhoven University of Technology, PO Box 513, 5600MB Eindhoven, The Netherlands

## Abstract

Following the substantial progress in molecular simulations of polymer-matrix nanocomposites, now is the time to reconsider this topic from a critical point of view. A comprehensive survey is reported herein providing an overview of classical molecular simulations, reviewing their major achievements in modeling polymer matrix nanocomposites, and identifying several open challenges. Molecular simulations at multiple length and time scales, working hand-in-hand with sensitive experiments, have enhanced our understanding of how nanofillers alter the structure, dynamics, thermodynamics, rheology and mechanical properties of the surrounding polymer matrices.

## Introduction

Polymer-matrix nanocomposites (PNCs) have drawn intense research interest over the last decade owing to both the rich fundamental physics associated with mixing macromolecules and particles and their unique mechanical, optical, magnetic and other material properties [1]. Driven by the need to develop functionally superior materials, significant effort has been invested in understanding the structure, dynamics, thermodynamics, rheology and mechanical properties of polymer-nanoparticle (NP) mixtures.

There are numerous excellent reviews of the field available [1–19]. The present overview, organized according to the answers to specific questions posed and not according to the simulation methods employed, aims at illustrating how molecular simulations have enhanced our understanding of the complex and fascinating field of PNCs.

### Polymer-Matrix Nanocomposites

In the simplest sense, a *composite* is an object made up of two or more distinct parts. Within materials science and engineering, composite materials are put together from two or more components that remain distinct or separate within the final product. Composites can be found anywhere, being as simple as a matrix material that envelops a reinforcing material, such as concrete surrounding steel bars, the latter preventing failure under tension. The *real challenge* is that the options in making a composite material are almost limitless, but only a few sets of materials will *combine synergistically*, and the design criteria may not be obvious. The observation that, other things being equal, the effectiveness of the filler increases with an increase in *surface to volume ratio* has provided large impetus to the shift from micron- to nanosized particles. With the appearance of synthetic methods that can produce nanometer sized fillers, resulting in an enormous increase of surface area, a new class of materials emerged, known as PNCs, i.e., polymer hosts filled with nanoparticles, which possess properties that typically differ significantly from those of the pure polymer, even at low nanoparticle concentrations [1, 15].

Nanocomposite materials contain particles of size $\alpha _\text{p} \sim 10 \;\text{nm}$ dispersed at a volume fraction, $\varphi$, often lower than $10^{-3}$ within a polymer matrix. They are thus characterized by particle number densities $\rho _\text{n} = 3\varphi /\left( 4 \pi \alpha _\text{p}^3\right) \approx 10^{20}\;\text{m}^{-3}$, interfacial areas per unit volume $3 \varphi /\alpha _\text{p} \approx 10^6\;\text{m}^{-1}$, and interparticle spacings, $\rho _\text{n}^{-1/3} - 2 \alpha _\text{p} \approx 100 \;\text{nm}$ that are commensurate with the particle dimensions, $\alpha _\text{p}$ and the radii of gyration of matrix chains, $R_\text{g} \approx 10 \;\text{nm}$.

The practice of adding nanoscale filler particles to reinforce polymeric materials can be traced back to the early years of the composite industry, in the second half of the 19th century. Charles Goodyear, inventor of vulcanized rubber, attempted to prepare nanoparticle-toughened automobile tires by blending carbon black, zinc oxide, and/or magnesium sulfate particles with vulcanized rubber [20]. Another example was the clay-reinforced resin known as Bakelite that was introduced in the early 1900s as one of the first mass-produced polymer–nanoparticle composites and fundamentally transformed the nature of practical household materials [21–24]. Then, a long period of time passed till the early 1990s when it was first demonstrated that the thermal and mechanical properties of Nylon-6 were improved by the addition of a few percent (2–4 % w/w) mica-type layered silicates to the extent that it could be used in an automotive engine compartment [25, 26].

Even though some property improvements have been achieved in nanocomposites, nanoparticle dispersion is difficult to control, with both thermodynamic and kinetic processes playing significant roles. It has been demonstrated that dispersed spherical nanoparticles can yield a range of multifunctional behavior, including a viscosity decrease, reduction of thermal degradation, increased mechanical damping, enriched electrical and/or magnetic performance, and control of thermomechanical properties [27–31]. The tailor-made properties of these systems are very important to the manufacturing procedure, as they fully overcome many of the existing operational limitations. As a final product, a polymeric matrix enriched with dispersed particles may have better properties than the neat polymeric material and can be used in more demanding and novel applications. Therefore, an understanding and quantitative description of the physicochemical properties of these materials is of major importance for their successful production.

As part of this renewed interest in nanocomposites, researchers also began seeking design rules that would allow them to engineer materials that combine the desirable properties of nanoparticles and polymers. In light of the diversity of polymers and nanoparticles, the potential for use of PNCs is nearly limitless. The ensuing research revealed a number of key challenges in producing nanocomposites that exhibit a desired behavior. The greatest stumbling block to the large-scale production and commercialization of nanocomposites is the dearth of cost-effective methods for controlling the *dispersion of the nanoparticles* in polymeric hosts. The nanoscale particles typically aggregate, which negates any benefits associated with the nanoscopic dimension. PNCs generally possess nonequilibrium morphologies due to the complex interplay of enthalpic and entropic interactions leading to particle aggregation, particle bridging interactions, and phase separation at various length scales [32, 33]. The second challenge is associated with understanding and predicting property enhancements in these materials, which are intimately connected to their morphology.

Nanocomposite research has recently expanded to consider more complicated systems involving polymer blends and block copolymers, where novel electrical, magnetic and optical properties arise [15, 34, 35].

### Multiscale Modeling

Understanding the fascinating and complex structure and dynamics of polymeric materials has been an ongoing challenge for many decades. From the point of view of molecular simulations, the spectrum of length and time scales associated with polymer melts of long chains poses a formidable challenge to studying their long-time dynamics [36, 37]. The topological constraints arising from chain connectivity and uncrossability (entanglements) dominate intermediate and long-time relaxation [38] and transport phenomena when polymers become sufficiently long. Atomistic molecular simulations of dense phases of soft matter prove to be difficult for many systems across length and time scales of practical interest. Even coarse-grained particle-based simulation methods may not be applicable due to the lack of faithful descriptions of polymer–polymer and polymer–surface interactions. Since complex interactions between constituent phases at the atomic level ultimately manifest themselves in macroscopic properties, a broad range of length and time scales must be addressed and a combination of modeling techniques is therefore required to simulate meaningfully the bulk-level behavior of nanocomposites [9].

*Soft condensed matter* is a relatively new term describing a huge class of rather different materials such as colloids, polymers, membranes, complex molecular assemblies, complex fluids etc. Though these materials are rather different in their structures, there is one unifying aspect, which makes it very reasonable to treat such systems from a common point of view. Compared to “hard matter” the characteristic *energy density is much smaller*. While the typical energy of a chemical bond (C–C bond) is about $10^{-18} \,\text{J} \approx 250 k_\text{B}T$ at room temperature of $300 \;\text{K}$, the nonbonded interactions are of the order of $k_\text{B}T$ and allow for strong density fluctuations even though the molecular connectivity is never affected ($k_\text{B}$ is the Boltzmann’s constant). It is instructive to compare the cohesive energy density, which gives a first estimate of the elastic constants, between a typical “hard matter” crystal to soft matter. The ratio between the two shows that polymeric systems are typically 100–10,000 times softer than classical crystals. As a consequence the *average thermal energy* $k_\text{B}T$ is not negligible for these systems any more, but rather *defines the essential energy scale*. This means that entropy, which typically contributes to the free energy a term of the order of $k_\text{B}T$ per degree of freedom, plays a crucial role. Especially in the case of macromolecules, this is mainly intramolecular entropy, which for a linear polymer of length *N* contributes to the free energy a term of order $N k_\text{B} T,$ representing about 90% of the free energy of polymeric materials [39]. As an immediate consequence it is clear that typical quantum chemical electronic structure calculations (Hartree-Fock or DFT) which focus on obtaining the energy as a function of nuclear coordinates cannot be sufficient to characterize soft condensed matter and will even be less sufficient to properly predict/interpret macroscopic properties. Molecular theoretical and simulation methods which incorporate entropic effects are required for this.

The length and time scales governing polymer physics range from Å and femtoseconds for the vibrations of atomic bonds to millimeters and seconds for crack propagation in polymer composites. The entities used as basic degrees of freedom are: electrons (quantum chemistry), atoms (classical forcefields), monomers or groups of monomers (coarse-grained or mesoscopic models) and entire polymer chains (soft fluids). All these methods and many others have been applied side by side to polymers. Until recently, however, multiscale methods with rigorous bridging between the different scales have been few.

#### Atomistic Molecular Dynamics (MD)

The stepping stone of classical molecular simulations is atomistic Molecular Dynamics (MD). As accurate MD potentials are developed for a broad range of materials based on quantum chemistry calculations and with the increase of supercomputer performance, atomistic MD simulations have become a very powerful tool for analyzing complex physical phenomena in polymeric materials, including dynamics, viscosity and shear thinning. However, as discussed above, entangled polymer systems are characterized by a wide range of spatial and temporal scales. It is still not feasible to equilibrate atomistic MD simulations of highly entangled polymer chain systems, due to their long relaxation times, long-range electrostatic interactions and tremendous number of atoms. The atomistic MD model for such a system, with a typical size of about a micrometer and a relaxation time on the scale of microseconds (or even up to the scale of seconds for long-chain polymer melts), would consist of billions of atoms and would require billions of time steps to run, which is obviously beyond the capability of the technique, even with the most sophisticated supercomputers available today.

#### Monte Carlo (MC)

A robust sampling of the configuration space of polymeric substances is a prerequisite for the reliable prediction of their physical properties. The constraints posed by atomistic MD simulations can be overcome by resorting to MC simulations, which enable us to use the complete arsenal of equilibrium statistical mechanics, e.g. perform sampling in all sorts of ensembles [36, 37, 40–42]. Through the design of efficient unphysical moves, configurational sampling can be dramatically enhanced. MC moves such as concerted rotation [43], configurational bias  [44, 45], and internal configurational bias [46] have thus successfully addressed the problem of equilibrating polymer systems of moderate chain lengths.

Even these moves prove incapable of providing equilibration when applied to long-chain polymer melts, however. A solution to this problem was given by the development and efficient implementation of a chain connectivity-altering MC move, end-bridging [47, 48]. Using end-bridging, atomistic systems consisting of a large number of long chains, up to C$_{6000}$, have been simulated in full atomistic detail [48, 49]. Despite its efficiency in equilibrating long-chain polymer melts, end-bridging cannot equilibrate monodisperse polymer melts; a finite degree of polydispersity is necessary for the move to operate. While this is not a drawback in modeling industrial polymers, which are typically polydisperse, an ability to equilibrate strictly monodisperse polymers is highly desirable for comparing against theory or model experimental systems. Morover, end-bridginig relies on the existence of chain ends, rendering itself inappropriate for dense phases of chains with nonlinear architectures. These limitations have been overcome by the introduction of Double Bridging (DB) and Intramolecular Double Rebridging (IDR) [50, 51]. The key innovation of those moves is the construction of two bridging trimers between two different chains, as far as the former is concerned, or along the same chain, as far as the latter move is concerned, thus preserving the initial chain lengths.

MC simulations using atomistic forcefields have inherent limitations, as Doxastakis et al. have shown [52]. The hard interactions between atoms reduce the acceptance rate of the moves. Thus, it is essential to resort to parallel tempering techniques in order to allow motion of the system in its phase space [53].

#### Coarse Graining (CG)

Polymers show a hierarchy of length and time scales. However, the connectivity in a polymer molecule enforces an interdependence between features on different scales. As a consequence, the choice of where one building block ends and where the next one begins is not unique, and it is not obvious how to abstract from a fundamental degree of freedom and use it in an implicit way in a coarser model. Thus, we will use the generic term *“coarse-grained”* for any model employing the idea of soft interacting particles (blobs) equal to or larger than the monomers constituting the polymeric chains.

The degree of coarse-graining is application-driven and describes the number of atoms/molecules in a typical blob considered by the coarse-grained model. It is closely related to the minimal features of the atomistic model that should be retained in order to reproduce the desired properties from the coarse-grained model. Mapping an atomistic model to a coarse-grained one is very important in defining the positions of coarse-grained particles and directly influences the parameterization of the coarse-grained force field.

A general procedure in coarse-graining usually involves: defining the observable of interest and determining the degree of coarse-graining; deciding an appropriate mapping of the atomistic model to the coarse-grained one; deriving interactions between the coarse-grained particles; reproducing target functions with the coarse-grained model; optimizing parameters/functions in the coarse-grained model and validating its range of applicability; conducting coarse-grained simulations.

#### Mesoscopic Simulations

A major challenge in simulating realistic PNCs is that neither the length nor the time scales can be adequately addressed by atomistic simulations alone, because of the extensive computational load. Until relatively recently, a somewhat neglected level of description in materials modeling has been the *mesoscopic* regime, lying between atomic (or super-atomic like) particles and finite element-based representations of a continuum, and covering characteristic length scales of $10^{-8} \;\text{}$–$10^{-5}\;\text{m}$. At this scale, the system is still too small to be regarded as a continuum, yet too large to be simulated efficiently using atomic models. In a more precise way, a *mesoscale* can be defined as an intermediate length scale at which the phenomena at the next level below (e.g. particle motions) can be regarded as having been equilibrated, and at which new phenomena emerge with their own characteristic time scales.

Among the several mesoscopic methods applied to the study of polymers, Self Consistent Field theory has been a well-founded tool [54]. This method adopts a field-theoretic description of the polymeric fluids and makes a saddle-point (mean-field) approximation. An alternative to invoking the saddle-point approximation is performing a normal Metropolis Monte Carlo (MC) simulation, with the effective potential energy of the system given by field-theoretic functionals. One of the first attempts has been made by Laradji et al. [55] for polymer brushes and then by Daoulas and Müller [56] and Detcheverry et al. [57, 58] for polymeric melts. The coordinates of all particles in the system are explicitly retained as degrees of freedom and evolve through MC moves. Tracking the motion of mesoscopic particles requires the use of stochastic dynamics [59].

## Selected Unresolved Issues in PNCs

PNCs have been an area of intense industrial and academic research for the past twenty years. Irrespectively of the measure employed - articles, patents, or funding-efforts in PNCs have been exponentially growing worldwide over the last 10 years. PNCs represent a radical alternative to conventional filled polymers or polymer blends-a staple of the modern plastics industry. Considering the multitude of potential nanoparticles, polymeric resins, and applications, the field of PNCs is immense [60]. The restricted class of polymer nanocomposites defined above still presents a complex and fascinating problem in statistical mechanics due to the richness of physical phenomena in mixtures of flexible polymer coils and hard impenetrable objects. Despite the unprecedented efforts placed on PNCs research there are still open questions which have not been definitely addressed yet. In the following we will summarize a few of them; later we will analyze the perspective simulations and theoretical calculations have provided us with.

Fundamental issues and questions include, but are not limited to: the packing and structure of dense mixtures of long polymer chains and hard impenetrable fillers, in the presence of attractive, neutral or repulsive interactions; perturbation of polymer packing and the possible nonexistence of a bulk region of the polymer matrix, especially in the case of PNC films; non-universal filler-induced polymer conformational changes triggered by interfacial effects and/or modification of the excluded volume screening mechanism of a pure polymer melt; the way in which geometric and chemical factors determine, in a nonadditive manner, the competing entropic and enthalpic contributions to the mixture free energy, miscibility and the physical nature of phase separated states. In all cases, the large particle surface-to-volume ratio leads to an amplification of a number of rather distinct molecular processes, implying pervasive interference between layers of polymers around nanoparticle surfaces.

### Segmental Dynamics and the Glass Transition Temperature

When cooling a glass forming liquid, instead of freezing at a well defined temperature, one observes a huge increase of the viscosity which takes place continuously. Such glass formers can be either simple liquids or polymer liquids, and many features are similar in both regarding the glass transition. One defines the *glass transition temperature*, $T_\text{g}$, as the temperature at which the dominant relaxation time on the molecular scale (or monomeric scale in the case of polymers) reaches about ~100 s, which corresponds typically to a viscosity of $10^{12} \;\text{Pa}\;\text{s}$ in the case of simple liquids. Typically for such glass forming liquids, the viscosity increases by twelve orders of magnitude over a change of temperature of about $100\;\text{K}$ down to $T_g$. The underlying mechanisms involved in this dramatic increase are still poorly understood [61, 62].

Experimental results on polymer dynamics and the glass transition in PNCs are not conclusive concerning the mechanism and the details of this modification. Increases or decreases in $T_\text{g}$ by as much as $30\;\text{K}$ [63] have been reported depending on polymer–nanoparticle interactions. Reduction of $T_\text{g}$ has been reported in the case of weak interactions between filler and polymer [64]. In other cases the addition of nanoparticles causes no significant change to the glass transition of the polymer, presumably because effects causing increase and decrease of polymer mobility are present simultaneously, effectively canceling out each other [65]. Moreover, strong interactions between the filler particles and the polymer suppress crystallinity, yielding new segmental relaxation mechanisms in semicrystalline polymers, originating from polymer chains restricted between condensed crystal regions and the semi-bound polymer in an interfacial layer with strongly reduced mobility [66].

Concerning the spatial extent of the $T_\text{g}$-shift, several studies [67, 68] on PNCs show an increase of the glass transition temperature, suggesting that the mobility of the entire volume of the polymer is restricted by the presence of the nanoparticles. However, there are many experimental results suggesting that the restriction of chain mobility caused by the nanoparticles does not extend throughout the material but affects only the chains within a few nanometers of the filler surface [69]. The existence of such an interfacial layer seems relatively well-established in the case of silica-filled elastomers, however its exact nature is not well understood: experimental results have been described in terms of one or two distinct interfacial layers or a gradual change in dynamics with changing distance from the particle.

### Enhancing Nanoparticle Dispersion by Surface Grafting

One of the biggest challenges is the rational control of *filler clustering* or *aggregation*, which often adversely affects material properties. The idea of achieving a good, uniform nanoparticle dispersion state has been the focus of considerable research, especially because of its favorable impact on optical and some mechanical properties of the resulting composites [70, 71]. In the past few years, several research groups have modified the surface of nanoparticle fillers in an effort to improve their dispersion in a polymer matrix. A promising strategy for controlling the dispersion and morphology of PNCs is to graft polymer chains onto the nanoparticles to form a brush layer [33]. The free chain/brush interfacial interactions may be “tuned” by controlling grafting density, $\sigma _\text{g}$, the degree of polymerization of the grafted chains, $N_\text{g}$, and of the polymer host, $N_\text{f}$, the nanoparticle size, $\alpha _\text{p}$, and its shape. For example, if nanoparticles are grafted with chains compatible with the matrix polymer, filler dispersion is favored [72–76]. Motivated by this concept, experimentalists have synthesized nanometer sized particles with high surface grafting density [74, 77, 78]. At fixed polymer chemistry, when the molecular weight of matrix polymer is lower than that of grafted polymer, nanoparticles disperse. On the contrary, if the molecular weight of the matrix polymer is higher than that of the grafted polymer, nanoparticles are thought to aggregate [74]. Since both the matrix and the brush have the same chemical structure, the immiscibility for longer matrix chains is entropic in origin and attributable to the concept of “brush autophobicity” [72, 79–83].

### Mechanical and Rheological Properties

The dispersion of micro- or nano-scale rigid particles within a polymer matrix often—but by no means always—produces an enhancement in the mechanical properties of these materials. As mentioned earlier, the most important application of this sort involves rigid inorganic particles (originally carbon black, later also silica) in a cross-linked elastomer matrix, where an improvement of mechanical properties is sought. This so-called *rubber reinforcement* is a complex phenomenon, which may involve an enhanced grip of tires on wet roads, an improved resistance to wear and abrasion, low rolling resistance, and an increase of tires’ ultimate mechanical strength (toughness, tearing resistance).

There is a variety of phenomena seeking an explanation. For the sake of readability, we will focus on a subset of them. Under very small cyclic deformations, there is a linear viscoelastic regime characterized by a very significant increase (sometimes even by two orders of magnitude compared to the reference unfilled network) of the in-phase storage modulus, both under elongation and under shear [84]. At medium-to-large strains, filled elastomers exhibit a markedly non-linear response which is absent in unfilled elastomers (“Payne effect”) [85]. The degree of non-linearity increases strongly with particle loading. An order-of-magnitude drop in the modulus is often observed on going to 5–10 % deformation (under shear), bringing the asymptotic modulus of the filled systems much closer to that of the reference unfilled network.

Other related effects are commonly observed in filled elastomers. One is deformation hysteresis (“Mullins effect”): under cyclic deformation, the elastic modulus in the first cycle is higher than that in the following ones [86]. This points to some kind of “damage” of the material, which, however, is often reversible. The original properties can be recovered within a few hours, by high-temperature annealing of the sample. Secondly, fillers affect also the dissipative, out-of-phase components of the modulus. This is expected, since, probably, friction of the polymer chains against the filler surfaces, or of two particles against each other produces new energy dissipation mechanisms, which are absent in unfilled elastomers. Elastic and dissipative effects likely share a common origin. Finally, reinforcement effects have a remarkable temperature dependence. The small-strain (linear) modulus of filled rubbers decreases with temperature, pointing to important enthalpic effects. The situation is completely reversed compared to unfilled elastomers, where the modulus increases linearly with absolute temperature due to the entropic nature of rubber elasticity.

## From Statistical Mechanics to Computer Simulations

Our discussion starts by introducing the formalism of statistical mechanics and briefly describing the basic methods used in computer simulations. We limit ourselves to the absolute minimum of definitions and methods to be presented, trying not to sacrifice consistency and rigor.

### Motion in Phase Space

Statistical physics describes a system of *N* particles at a given state as one point in 6*N*/dimensional phase space, containing the atom positions and momenta (and neglecting the internal degrees of freedom of atoms) [87]. In classical mechanics, the state of the system is completely specified in terms of a set of *generalized coordinates* $\{ \mathbf {q}_i \}$ and *generalized momenta* $\{ \mathbf {p}_i \}$, where $i = 1, \dots , N$ [88]. We will refer to the 3*N*-dimensional set from which the generalized coordinates of the system $\{\mathbf {q}\} \equiv \left\{ \mathbf {q}_1, \mathbf {q}_2, \dots , \mathbf {q}_N\right\}$ take on values as *configuration space*, and to the 3*N*-dimensional set from which the generalized momenta $\{\mathbf {p}\} \equiv \left( \mathbf {p}_1, \mathbf {p}_2, \dots , \mathbf {p}_N\right)$ take on values as *momentum space*. Any instantaneous *microscopic* state of the system can be written as a point:

1 $$\begin{aligned} \Gamma = \left( \left\{ \mathbf {q}_i\right\} , \left\{ \mathbf {p}_i\right\} \right) \end{aligned}$$

in the *phase space* of the system. The set of values of the macroscopic observables, such as temperature, pressure, etc., describes the system’s *macroscopic* state. One macroscopic state corresponds to all the microscopic states that provide the same values of the macroscopic observables, defined by the macroscopic state.

If we know the *Hamiltonian*, $\mathscr{H}\left( \left\{ \mathbf {q}_i \right\} , \left\{ \mathbf {p}_i\right\} , t\right)$, for the system, then the time evolution of the quantities $\mathbf {q}_i$ and $\mathbf {p}_i$ ($i = 1, \dots , N$) is given by Hamilton’s equations of motion

2 $$\begin{aligned} \dot{\mathbf {p}_i} \equiv \frac{\partial \mathbf {p}_i}{\partial t} = - \frac{\partial \mathscr {H}\left( \left\{ \mathbf {q}_i \right\} , \left\{ \mathbf {p}_i\right\} , t\right) }{\partial \mathbf {q}_i } \end{aligned}$$

and

3 $$\begin{aligned} \dot{\mathbf {q}_i} \equiv \frac{\partial \mathbf {q}_i}{\partial t} = \frac{\partial \mathscr {H}\left( \left\{ \mathbf {q}_i \right\} , \left\{ \mathbf {p}_i\right\} , t\right) }{\partial \mathbf {p}_i} \end{aligned}$$

where $i= 1, 2, \dots , N$ and $\partial / \partial \mathbf {x} \equiv \nabla _{\mathbf {x}}$ symbolizes the gradient operator with respect to the vectorial quantity $\mathbf {x}$. As the system evolves in time and its state changes, the system point traces out a trajectory in $\Gamma$-space. Since the subsequent motion of a classical system is uniquely determined by the initial conditions, it follows that no two trajectories in phase space can cross. If the Hamiltonian $\mathscr {H}$ does not depend explicitly on time, then $\mathscr {H}$ is a constant of the motion. Such is the case for *conservative* systems.

#### Time Average

Any property of the system, $\mathscr {A}$, is then a function of the points traversed by the system in phase space. The instantaneous property at a time *t* is $\mathscr {A}\left( \Gamma \left( t\right) \right)$ and the macroscopically meaningful observable property $\mathscr {A}_\text{obs}$ is the time average of this,

4 $$\begin{aligned} \mathscr {A}_\text{obs} = \left\langle \mathscr {A}\left( \Gamma \left( t\right) \right) \, \right\rangle _{t} = \lim _{t_\text{obs} \rightarrow \infty } \frac{1}{t_\text{obs}} \int _0^{t_\text{obs}} \mathscr {A}\left( \Gamma \left( t\right) \right) \text{d}t \end{aligned}$$

In experiments, the *time average* comes about quite naturally, since almost all experimental methods measure over much longer time scales than the longest relaxation time of the system. A straightforward approach, in order to get $\mathscr {A}$ from molecular simulations, is to determine a time average, taking a discrete sum over *M* time steps of length $\Delta t$:

5 $$\begin{aligned} \mathscr {A}_\text{obs} \simeq \lim _{M \rightarrow \infty } \frac{1}{M \Delta t} \sum _{j = 1}^{M} \mathscr {A} \left( \Gamma \left( j \Delta t\right) \right) \Delta t \end{aligned}$$

This is the approach undertaken in Molecular Dynamics (MD) simulations, where the atoms’ trajectory is followed as a function of time, so it is straightforward to obtain the average.

#### Phase Space Probability Density

When we deal with real systems, we can never specify exactly the state of the system, despite the deterministic character of its motion in phase space. There will always be some uncertainty in the initial conditions. Therefore, it is useful to consider $\Gamma$ as a stochastic variable and to introduce a probability density $\rho \left( \Gamma , t\right)$ on the phase space. In doing so, we envision the phase space filled with a continuum (or fluid) of state points. If the fluid were composed of individual discrete points, then each point would be equipped with a probability in accordance with our initial knowledge of the system and would carry this probability for all time, since probability is conserved. Because state points must always lie somewhere in the phase space, we have the normalization condition

6 $$\begin{aligned} \displaystyle \int _\Gamma \rho \left( \Gamma , t\right) \text{d}\Gamma \equiv \displaystyle \int _\Gamma \rho \left( \{ \mathbf {q}_i \}, \{ \mathbf {p}_i \}, t \right) \text{d}^{3N} p \; \text{d}^{3N} q = 1 \end{aligned}$$

where the integration takes place over the entire phase space. Similarly, the probability of finding the system in a small finite region *D* of $\Gamma$-space at time *t* is found by integrating the probability density over that region:

7 $$\begin{aligned} P \left( D, t\right) = \displaystyle \int _D \rho \left( \{\mathbf {q}_i \}, \{\mathbf {p}_i \}, t\right) \text{d}^{3N} p \; \text{d}^{3N} q \end{aligned}$$

The probability density for finding a system in the vicinity of $\Gamma$ depends on the macroscopic state of the system, i.e. on the macroscopic constraints defining the system’s size, spatial extent, and interactions with its environment. A set of microscopic states distributed in phase space according to a certain probability density is called an *ensemble*. A very important measure of the probability distribution of an equilibrium ensemble is the *partition function* *Q*. This appears as a normalizing factor in the probability distribution defined by the ensemble.

#### Ensemble Average

The *ergodic hypothesis*, originally due to L. Boltzmann [89], states that, over long periods of time, the time spent by a system in some region of the phase space of microstates with the same energy is proportional to the volume of that region, i.e., that all accessible microstates are equiprobable over a long period of time. Ergodicity is based on the assumption (provable for some Hamiltonians) that any dynamical trajectory, given sufficient time, will visit all “representative” regions in phase space, the density distribution of points in phase space traversed by the trajectory converging to a stationary distribution.

According to the ergodic hypothesis we can calculate the observables of a system in equilibrium as averages over phase space with respect to the probability density of an equilibrium ensemble, $\rho _\text{ens} \left( \Gamma \right)$. If $\rho _\text{ens}\left( \Gamma \right)$ obeys the normalization condition, Eq. (6), on the entire phase space $\Gamma$ and also is zero for all points outside the hypersurface $\mathscr {H}\left( \Gamma \right) = E$, the ensemble average can be defined as:

8 $$\begin{aligned} \mathscr {A}_\text{obs} = \left\langle \mathscr {A} \right\rangle _\text{ens} = \int \mathscr {A} \left( \Gamma \right) \rho _\text{ens}\left( \Gamma \right) \text{d}\Gamma \end{aligned}$$

In Monte Carlo (MC) simulations, the desired thermodynamic quantities are determined as *ensemble averages*:

9 $$\begin{aligned} \left\langle \mathscr {A} \right\rangle _\text{ens} = \frac{\sum \limits _{\Gamma } \mathscr {A} \left( \Gamma \right) \rho _\text{ens} \left( \Gamma \right) }{\sum \limits _{\Gamma } \rho _\text{ens} \left( \Gamma \right) } \end{aligned}$$

If we wish to obtain an average over points in phase space, there is no need to simulate any real time dependence of the system; one need only construct a sequence of states in phase space in the correct ensemble. In the context of equilibrium simulations, it is always important to make sure that the algorithm used in the simulation is ergodic. This means that no particular region in phase space should be excluded from sampling by the algorithm. Such an exclusion would render the simulation wrong, even if the simulated object itself is ergodic. From a practical point of view, the ergodicity of the system can and should be checked through reproducibility of the calculated thermodynamic properties (pressure, temperature, etc.) in runs with different initial conditions.

### Statistical Ensembles

#### Microcanonical (*NVE*) Ensemble

In the *microcanonical* (*NVE*) ensemble the number of particles, *N*, the volume of the system, *V* and the total energy, *E*, are conserved. This corresponds to a completely closed system which does not interact in any way with the environment and lies in a container of fixed volume, *V*. For simplicity, we neglect the intramolecular degrees of freedom. Then, the system energy will be a sum of kinetic, $\mathscr {K}$, and potential, $\mathscr {V}$ energies. Since the total energy *E* must be conserved, the criterion for adding states in the ensemble would be

10 $$\begin{aligned} \mathscr {H} \left( \{ \mathbf {q}_i \}, \{ \mathbf {p}_i \} \right) = \,&\mathscr {K}\left( \{ \mathbf {q}_i \}, \{ \mathbf {p}_i \} \right) + \mathscr {V}\left( \{ \mathbf {q}_i \}, \{ \mathbf {p}_i \} \right) \nonumber \\ = &\text{ constant} = E_0 \end{aligned}$$

which means that not all, but only those states in phase space $\Gamma$ that have total energy $E_0$ are allowed. This can also be stated so that the probability density of the ensemble is

11 $$\begin{aligned} \rho _{NVE} \left( \{ \mathbf {q}_i \}, \{ \mathbf {p}_i \} \right) = \frac{1}{Q_{NVE}} \delta \left[ \mathscr {H} \left( \{ \mathbf {q}_i \}, \{ \mathbf {p}_i \} \right) - E_0\right] \end{aligned}$$

where $\delta$ is the Kronecker delta for a discrete system, and the Dirac delta function for a continuous one. The partition function in the microcanonical ensemble, $Q_{NVE}$, is:

12 $$\begin{aligned} Q_{NVE} = \sum _{\Gamma } \delta \left[ \mathscr {H} \left( \Gamma \right) - E_0\right] \end{aligned}$$

The summation over states, $\sum _\Gamma$, is used if microscopic states are discrete and $\rho \left( \Gamma \right)$ has the meaning of a probability. For one-component classical systems, the sum can be replaced by an integral, yielding

13 $$\begin{aligned} Q_{NVE}&= \frac{1}{N!} \frac{1}{h^{3N}} \int \text{d}\Gamma \delta \left[ \mathscr {H} \left( \Gamma \right) - E_0\right] \nonumber \\&= \frac{1}{N!} \frac{1}{h^{3N}} \int \prod _{i=1}^N \text{d}^3 r_i \; \text{d}^3 p_i \; \delta \left[ \mathscr {H} \left( \{ \mathbf {q}_i \}, \{ \mathbf {p}_i \} \right) - E_0\right] \end{aligned}$$

where *N*! takes care of the indistinguishability of particles of the same species and $h^{3N}$ is the ultimate resolution for counting states allowed by the uncertainty principle.

The proper thermodynamic potential for the microcanonical ensemble is the *entropy*:

14 $$\begin{aligned} S = k_\text{B} \ln {\left( Q_{NVE} \right) } \end{aligned}$$

where $k_\text{B}$ is the Boltzmann constant. We therefore have a statistical thermodynamic definition of entropy as a quantity proportional to the logarithm of the number of microscopic states under given *N*, *V*, *E*. Eq. (14) establishes a fundamental thermodynamic equation in the entropy representation.

#### Canonical (*NVT*) Ensemble

In the *canonical* (*NVT*) ensemble the number of particles, *N*, the volume of the system, *V*, and temperature, *T* are conserved. This corresponds to a closed system, which, however, can exchange heat with a large surrounding bath. The energy is fluctuating, but the temperature is constant, describing the probability distribution of energy fluctuations. The total energy of the system is given by its Hamiltonian, $\mathscr {H} \left( \{ \mathbf {q}_i \}, \{ \mathbf {p}_i \} \right)$. The probability density of the ensemble is:

15 $$\begin{aligned} \rho _{NVT}\left( \{ \mathbf {q}_i \}, \{ \mathbf {p}_i \}\right) = \frac{1}{Q_{NVT}} \frac{1}{N! h^{3N}} \exp {\left[ -\frac{\mathscr {H}\left( \{ \mathbf {q}_i \}, \{ \mathbf {p}_i \}\right) }{k_\text{B} T} \right] } \end{aligned}$$

with $k_\text{B}$ being the Boltzmann’s constant and $Q_{NVT}$ the partition function in the *NVT* ensemble:

16 $$\begin{aligned} Q_{NVT} = \frac{1}{N! h^{3N}} \int \prod _{i=1}^N \text{d}^3 q_i \; \text{d}^3 p_i \; \exp {\left[ -\frac{\mathscr {H}\left( \{ \mathbf {q}_i \}, \{ \mathbf {p}_i \}\right) }{k_\text{B} T} \right]. } \end{aligned}$$

The thermodynamic function of the system is the *Helmholtz energy*:

17 $$\begin{aligned} A = - k_\text{B} T \ln {\left( Q_{NVT}\right) } \end{aligned}$$

Eq. (17) defines a fundamental equation in the Helmholtz energy representation by expressing *A* as a function of *N*, *V*, *T*.

#### Isothermal - Isobaric Ensemble (*NpT*)

The isothermal-isobaric ensemble describes the equilibrium distribution in phase space of a system under constant number of particles, temperature, and pressure. The volume of the system is allowed to fluctuate. Thus, a point in phase space is defined by specifying *V*, $\left\{ \mathbf {q}_i\right\}$ and $\left\{ \mathbf {p}_i\right\}$, where the domain from which the $\mathbf {q}_i$s take on values depend on the value of *V*.

The probability density of the *NpT* ensemble can be derived from that of the microcanonical ensemble, by considering a bath around the system which acts as both a heat and a work reservoir for the system under study. The probability density, in a classical statistical mechanical formulation, is:

18 $$\begin{aligned} \rho _{NpT} \left( \{ \mathbf {q}_i \}, \{ \mathbf {p}_i \} ; V\right) =&\frac{1}{Q_{NpT}} \nonumber \\& \times \exp {\left[ -\frac{\mathscr {H}\left( \{ \mathbf {q}_i \}, \{ \mathbf {p}_i \} \right) +pV}{k_\text{B}T} \right] } \end{aligned}$$

where $Q_{NpT}$ is the *isothermal-isobaric partition function*:

19 $$\begin{aligned} Q_{NpT} = \frac{1}{N! h^{3N}} \frac{1}{V_0}&\displaystyle \int \text{d}V \displaystyle \int \prod _{i=1}^N \text{d}^3 q_i \; \text{d}^3 p_i \nonumber \\& \times \exp {\left[ -\frac{\mathscr {H}\left( \{ \mathbf {q}_i \}, \{ \mathbf {p}_i \} \right) +pV}{k_\text{B}T} \right] } \end{aligned}$$

where $V_0$ denotes some basic unit of volume introduced to make the partition function dimensionless (the exact magnitude of $V_0$ is immaterial).

The connection between the formalism of the isothermal-isobaric ensemble and macroscopic thermodynamic properties is established via the Gibbs energy:

20 $$\begin{aligned} G\left( N, p, T\right) = - k_\text{B}T \ln {\left( Q_{NpT} \left( N, p, T \right) \right) } \end{aligned}$$

#### Configurational Integral

As long as the *Born-Oppenheimer approximation* [90] is valid (as it practically always is in equilibrium thermodynamics) the potential energy of the system, $\mathscr {V}\left( \Gamma \right)$, depends only on the generalized coordinates, $\left\{ \mathbf {q}_i \right\}$. Similarly, the kinetic energy, $\mathscr {K}\left( \Gamma \right)$, depends only on the momenta $\left\{ \mathbf {p}_i \right\}$. Hence we can rewrite the expression for the system Hamiltonian as:

21 $$\begin{aligned} \mathscr {H}\left( \Gamma \right) = \mathscr {K} \left( \left\{ \mathbf {p}_i\right\} \right) + \mathscr {V}\left( \left\{ \mathbf {q}_i \right\} \right) \end{aligned}$$

It can be now seen that, in a classical (as opposed to quantum mechanical) treatment, the partition function, e.g. of the *NVT* ensemble, factorizes into a product of kinetic (ideal gas) and potential (excess) parts:

22 $$\begin{aligned} Q_{NVT} = \frac{1}{N!}\frac{1}{h^{3N}}&\displaystyle \int \prod _{i=1}^{N} \text{d}^3 p_i \; \exp {\left[ -\frac{\mathscr {K}\left( \left\{ p_i\right\} \right) }{k_\text{B} T} \right] } \nonumber \\ & \times \displaystyle \int \prod _{i=1}^{N} \text{d}^3 q_i \exp {\left[ -\frac{\mathscr {V}\left( \left\{ q_i\right\} \right) }{k_\text{B} T} \right] } \end{aligned}$$

This can be written as a product of the ideal gas contribution, and the excess contribution as:

23 $$\begin{aligned} Q_{NVT} = Q_{NVT}^\text{id} V^{-N} \mathscr {Z}_{NVT} \end{aligned}$$

where:

24 $$\begin{aligned} \mathscr {Z}_{NVT} = \displaystyle \int \prod _{i=1}^{N} \text{d}^3 q_i \, \exp {\left[ -\frac{\mathscr {V}\left( \left\{ q_i\right\} \right) }{k_\text{B} T} \right] } \end{aligned}$$

is the so called *configurational integral*. The partition function of the ideal gas is:

25 $$\begin{aligned} Q_{NVT} = \frac{V^N}{N! \Lambda ^{3N}} \end{aligned}$$

with $\Lambda$ being the *de Broglie* or *thermal wavelength*:

26 $$\begin{aligned} \Lambda = \left( \frac{h^2}{2 \pi m k_\text{B} T}\right) ^{1/2} \end{aligned}$$

From the perspective of a particle-based model, the fundamental problem of equilibrium statistical mechanics, according to Chandler [91], is to evaluate a configurational partition function of the form of Eq. (24).

Two important consequences arise from Eq. (23). First, all the thermodynamic properties can be expressed as a sum of an ideal gas part and an excess part. The chemical details which govern the interactions between the atoms of the system are included in the latter. In fact, in MC simulations the momentum part of the phase space is usually omitted, and all calculations are performed in configuration space. The second important consequence of Eq. (23) is that the total average kinetic energy is a universal quantity, independent of the interactions in the system. Indeed, computing the average of

27 $$\begin{aligned} \mathscr {K} = \sum _{i = 1}^N \frac{\mathbf {p}_i^2}{2 m} \end{aligned}$$

with respect to the probability distribution of Eq. (15) and using the factorization of Eq. (23) we obtain that [87]:

28 $$\begin{aligned} \left\langle \mathscr {K} \right\rangle = \frac{3}{2} N k_\text{B} T \end{aligned}$$

or, more generally $\langle \mathscr {K} \rangle = 1/2 N_\text{dof} k_\text{B} T$ for a system of $N_\text{dof}$ degrees of freedom.^1^

## Simulation Methods

### Molecular Dynamics

In Cartesian coordinates, and under the assumption that the potential energy $\mathscr {V}$ is independent of velocities and time, Hamilton’s equations of motion read:

29 $$\begin{aligned} \dot{\mathbf {r}}_i&\equiv \mathbf {v}_i = \frac{\mathbf {p}_i}{m_i} \end{aligned}$$

30 $$\begin{aligned} \dot{\mathbf {p}}_i&\equiv - \frac{\partial \mathscr {V}}{\partial \mathbf {r}_i} = \mathbf {F}_i \end{aligned}$$

hence

31 $$\begin{aligned} m_i \ddot{\mathbf {r}}_i = \mathbf {F}_i \end{aligned}$$

where $\mathbf {F}_i$ is the force acting on atom *i*:

32 $$\begin{aligned} \mathbf {F}_i = - \nabla _{\mathbf {r}_i} \mathscr {V} \end{aligned}$$

with the gradient being taken keeping all positions other than $\mathbf {r}_i$ constant. Solving the equations of motion then involves the integration of 3*N* second-order differential equations Eq. (31) which are Newton’s equations of motion.

The classical equations of motion possess some interesting properties, the most important one being the conservation law. If we assume that $\mathscr {K}$ and $\mathscr {V}$ do not depend explicitly on time, then it is straightforward to verify that $\dot{\mathscr {H}} = \text{d}\mathscr {H} / \text{d}t$ is zero, i.e., the Hamiltonian is a constant of the motion. In actual calculations this conservation law is satisfied if there exist no explicitly time- or velocity-dependent forces acting on the system. A second important property is that Hamilton’s equations of motion are reversible in time. This means that, if we change the signs of all velocities, we will cause the molecules to retrace their trajectories backwards. The computer-generated trajectories should also possess this property.

Concerning the solution of equations of motion, in the limit of very long times, it is clear that no algorithm provides an essentially exact solution. However, this turns out to be not a serious problem, because the main objective of an MD simulation is not to trace the exact configuration of a system after a long time, but rather to predict thermodynamic properties as time averages and calculate time correlation functions representative of the dynamics.

In the following we briefly describe the most popular family of algorithms used in MD simulations for the solution of classical equations of motion: the *Verlet algorithms*. Another family of algorithms comprises higher-order methods, whose basic idea is to use information about positions and their first, second, and higher order time derivatives at time *t* in order to estimate the positions and their derivatives at time $t + \Delta t$ [93].

In general, higher-order methods are characterized by a much better accuracy than the Verlet algorithms, particularly at small times. However, their main drawback is that they are not reversible in time, which results in insufficient energy conservation, especially in very long-time MD simulations. On the contrary, the Verlet methods are not essentially exact for small times but their inherent time reversibility guarantees that the energy conservation law is satisfied even for very long times. This feature renders the Verlet methods, and particularly the velocity-Verlet algorithm, the most appropriate ones to use in long atomistic MD simulations.

#### Verlet Algorithm

The initial Verlet algorithm [94] ends up calculating the positions at time $t + \Delta t$ by using two Taylor expansions around times $t - \Delta t$ and $t + \Delta t$, respectively:

33 $$\begin{aligned} \mathbf {r}_i \left( t - \Delta t\right)&= \mathbf {r}_i\left( t\right) - \mathbf {v}_i \left( t\right) \Delta t + \frac{\mathbf {F}_i \left( t\right) }{2 m_i} \Delta t^2 \nonumber \\& \quad - \dddot{\mathbf {r}}_i \left( t\right) \frac{\Delta t^3}{3!} + \mathscr {O}\left( \Delta t^4\right) \end{aligned}$$

34 $$\begin{aligned} \mathbf {r}_i \left( t + \Delta t \right)&= \mathbf {r}_i\left( t\right) + \mathbf {v}_i \left( t\right) \Delta t + \frac{\mathbf {F}_i \left( t\right) }{2 m_i} \Delta t^2 \nonumber \\& \quad + \dddot{\mathbf {r}}_i \left( t\right) \frac{\Delta t^3}{3!} + \mathscr {O}\left( \Delta t^4\right) \end{aligned}$$

Summing these two equations we obtain:

35 $$\begin{aligned} \mathbf {r}_i \left( t+ \Delta t \right) \approx 2 \mathbf {r}_i \left( t\right) - \mathbf {r}_i \left( t - \Delta t\right) + \frac{\mathbf {F}_i \left( t\right) }{m_i} \Delta t^2 \end{aligned}$$

The estimate of the new positions contains an error that is in the order of $\Delta t^4$, where $\Delta t$ is the time step employed in our MD scheme. It should be noted that the Verlet algorithm does not use the velocities to compute the new positions. One can, however, derive the velocities from knowledge of the trajectory, using

36 $$\begin{aligned} \mathbf {v}_i \left( t\right) = \frac{\mathbf {r}_i \left( t+\Delta t\right) - \mathbf {r}_i \left( t - \Delta t\right) }{2 \Delta t} + \mathscr {O}\left( \Delta t^2\right) \end{aligned}$$

which is only accurate to order $\Delta t^2$.

#### Velocity-Verlet Algorithm

The problem of defining the positions and velocities at the same time can be overcome by casting the Verlet algorithm in a different way. This is the Velocity-Verlet algorithm [95], according to which positions are obtained through the usual Taylor expansion

37 $$\begin{aligned} \mathbf {r}_i\left( t + \Delta t\right) = \mathbf {r}_i\left( t\right) + \mathbf {v}_i \left( t\right) \Delta t + \ddot{\mathbf {r}}_i \left( t\right) \frac{\Delta t^2}{2} \end{aligned}$$

whereas velocities are calculated through

38 $$\begin{aligned} \mathbf {v}_i\left( t + \Delta t\right) = \mathbf {v}_i\left( t\right) + \frac{\Delta t}{2} \left[ \ddot{\mathbf {r}}_i\left( t\right) + \ddot{\mathbf {r}}_i\left( t+\Delta t\right) \right] \end{aligned}$$

with all accelerations computed from the forces at the configuration corresponding to the considered time.

### Langevin Dynamics

When a large system is simulated, it is generally desired to keep the number of degrees of freedom as low as possible. If a certain subset of particles can be distinguished, of which details of the motion are not relevant, these particles can be omitted from a detailed MD simulation. However, the forces they exert on the remaining particles must be represented as faithfully as possible. This means that correlations of such forces with positions and velocities of particle *i* must be incorporated in the equations of motion of particle *i*, while uncorrelated contributions can be represented by random forces. This brings us to the field of Langevin Dynamics (LD) [96, 97]. In LD a frictional force, proportional to the velocity, is added to the conservative force, in order to mimic an implicitly treated background (e.g. solvent). The friction removes kinetic energy from the system. In order to compensate for the friction, a random force adds kinetic energy to the system.

In the simplest case of LD, the random force is taken to have white-noise character, and no correlations between the various degrees of freedom are assumed to exist. Under these conditions, the velocity dependent frictional forces become proportional to the instantaneous velocity of the particle considered. Thus, the equation of motion of a particle *i* is transformed into the stochastic equation:

39 $$\begin{aligned} m_i \dot{\mathbf {v}_i}\left( t\right) = \mathbf {F}_i \left( \left\{ \mathbf {r}_i \left( t\right) \right\} \right) - \zeta _i \mathbf {v}_i \left( t\right) + \varvec{\mathscr {F}}_i\left( t\right) \end{aligned}$$

where the friction coefficient of a particle is denoted by $\zeta _i$ and the random force by $\varvec{\mathscr {F}}_i$. The systematic force $\mathbf {F}_i$ is the explicit mutual force between the *N* particles of the system, which is to be derived from the potential (or free) energy of the system, which depends on the positions of all particles, denoted by $\left\{ \mathbf {r}_i \right\}$.

The stochastic force, $\varvec{\mathscr {F}}_i\left( t\right)$, is assumed to be a stationary Gaussian random variable with zero mean and to have no correlations with prior velocities or with the systematic force:

40 $$\begin{aligned}&\left\langle \varvec{\mathscr {F}}_{i,\alpha } \left( 0\right) \varvec{\mathscr {F}}_{j,\beta } \left( t\right) \right\rangle _\text{ens} = 2 \zeta _i k_\text{B} T_\text{ref} \delta _{ij} \delta _{\alpha \beta } \delta \left( t\right) \end{aligned}$$

41 $$\begin{aligned}&W\left( \varvec{\mathscr {F}}_{i,\alpha}\right) = \left( 2 \pi \left\langle \varvec{\mathscr {F}}_{i,\alpha}^2 \right\rangle _\text{ens}\right) ^{-1/2} \exp {\left( -\frac{\varvec{\mathscr {F}}_{i,\alpha}^2}{2 \left\langle \varvec{\mathscr {F}}_{i,\alpha}^2 \right\rangle _\text{ens}}\right) } \end{aligned}$$

42 $$\begin{aligned}&\left\langle \varvec{\mathscr {F}}_i \right\rangle _\text{ens} = 0 \end{aligned}$$

43 $$\begin{aligned}&\left\langle v_{i,\alpha } \left( 0\right) \varvec{\mathscr {F}}_{j,\beta } \left( t\right) \right\rangle _\text{ens} = 0, \;\;\;\; t \ge 0 \end{aligned}$$

44 $$\begin{aligned}&\left\langle F_{i,\alpha } \left( 0\right) \varvec{\mathscr {F}}_{j,\beta } \left( t\right) \right\rangle _\text{ens} = 0, \;\;\;\; t \ge 0 \end{aligned}$$

where $\left\langle \dots \right\rangle _\text{ens}$ denotes averaging over an equilibrium ensemble, indices $\alpha$, $\beta$ run over the Cartesian components (*x*, *y* and *z*), $k_\text{B}$ is Boltzmann’s constant, $T_\text{ref}$ is the reference temperature of the LD simulation and $W\left( \varvec{\mathscr {F}}\right)$ is the (Gaussian) probability distribution of the stochastic force. According to van Gunsteren et al. [98], the solution of the linear, inhomogeneous, first order differential equation, Eq. (39), is:

45 $$\begin{aligned} \mathbf {v}\left( t\right) = & {\mathbf{v}}_i \left( 0\right) \exp {\left( - \frac{\zeta _i}{m_i} t\right) } \nonumber \\ +&\frac{1}{m_i} \int _{0}^t \exp {\left[ - \frac{\zeta _i}{m_i} \left( t-t^\prime \right) \right] } \left[ \mathbf {F}_i \left( t^\prime \right) + \varvec{\mathscr {F}}_i \left( t^\prime \right) \right] \text{d}t^\prime \end{aligned}$$

#### Fluctuation-Dissipation Theorem

To generate a canonical ensemble, the friction and random force have to obey the *fluctuation - dissipation theorem* [99]. Einstein was the first to extract the diffusion coefficient and mobility in a special case of Brownian motion [100], and made allusions to the existence of a balance between random forces and friction. Then, Nyquist [101] formulated a limited version of the theorem, in his study of noise in resistors. Later, Callen and Welton [102] proved the theorem in a generalized form.

According to Kubo [103], two different kinds of the fluctuation-dissipation theorem can be distinguished. The fluctuation-dissipation theorem of the first kind relates the linear response of a system to an externally applied perturbation and a two-time correlation function of the system in the absence of external forces. The latter form is closely related to the famous Green-Kubo expressions for transport coefficients. The fluctuation-dissipation theorem of the second kind constitutes a relationship between the frictional and random forces in the system, relying on the assumption that the response of a system in thermodynamic equilibrium to a small applied perturbation is the same as its response to a spontaneous fluctuation [59].

#### Mori-Zwanzig Projection Operator Formalism

A formal way of deriving LD is the *projection operator formalism* of Zwanzig [104, 105] and Mori [106, 107]. The basis of the formulation is the assumption that we have partial knowledge of the evolving system, for example we can only track certain variables, while the effect of the other variables is modeled or approximated in a rigorous way. In this approach the phase space is divided into two parts, which we are called *interesting* and *uninteresting* degrees of freedom. For the approach to be useful, the uninteresting degrees of freedom should be rapidly varying in comparison to the interesting ones. Mori introduced two projection operators, which project the whole phase space onto the sets of interesting and uninteresting degrees of freedom. The full equations of motion are projected only onto the set of interesting degrees of freedom. The result is a differential equation with three force terms: a mean force between the interesting degrees of freedom, a dissipative or frictional force exerted by the uninteresting degrees of freedom onto the interesting ones and a third term containing forces not correlated with the interesting degrees. When the uncorrelated force is approximated by a random force the interesting degrees of freedom are considered independent of the uninteresting degrees of freedom.

### Brownian Dynamics

If the friction exerted by the background to the particles under consideration is high, correlations in the velocity will decay in a time period over which changes in the systematic force are negligible. Such a system can be called *overdamped*. In this case, the left-hand side of Eq. (39) can be neglected, after averaging over short times. The result is Brownian Dynamics (BD), which is described by the position Langevin equation:

46 $$\begin{aligned} \mathbf {v}_i \left( t\right) = \frac{1}{\zeta _i} \mathbf {F}_i \left( \left\{ \mathbf {r}_i\left( t\right) \right\} \right) + \frac{1}{\zeta _i} \varvec{\mathscr {F}}_i\left( t\right) \end{aligned}$$

The time scale separation makes possible the exchange of the second order stochastic differential equation Eq. (39) for a first order stochastic differential equation, Eq. (46), without affecting the dynamics on time scales longer than $m_i / \zeta _i$.

Van Gunsteren and Berendsen [108, 109] have proposed several algorithms for integrating Eq. (46). We will pay a closer look to the one which reduces to the Verlet algorithm for zero friction. If we assume a timestep of $\Delta t$, for large values of $\zeta _i / m_i \Delta t$ in the diffusive regime, when the friction is so strong that the velocities relax within $\Delta t$, the BD algorithm reduces to:

47 $$\begin{aligned} r_{i, \alpha } \left( t_n + \Delta t\right) =&r_{i,\alpha }\left( t_n\right) \nonumber \\ +&\frac{1}{\zeta _i}\left[ F_{i,\alpha } \left( t_n\right) \Delta t + \frac{1}{2} \dot{F}_{i,\alpha } \left( t_n\right) \left( \left( \Delta t\right) ^2\right) \right] \nonumber \\ +&\mathscr {R}_{i,\alpha } \left( \Delta t\right) \end{aligned}$$

with *i* enumerating the particles, $1 \le i \le N$, and $\alpha$ marking a Cartesian component of the vectors. The components of the random displacement $\mathscr {R}\left( \Delta t\right)$ are sampled from a Gaussian distribution with zero mean and width:

48 $$\begin{aligned} \left\langle \mathscr {R}_{i,\alpha }^2 \left( \Delta t\right) \right\rangle = \frac{2 k_\text{B} T_\text{ref}}{\zeta _i} \Delta t \end{aligned}$$

The reader is reminded that the integration timestep $\Delta t$ should be small enough, such that systematic forces do not change significantly over its duration. The integration scheme for BD Eq. (47) resembles a MC algorithm, except that there is no acceptance criterion. Rossky et al. [110] have derived the correct acceptance probability and introduced their method under the name “Smart Monte Carlo”.

### Dissipative Particle Dynamics

Molecular Dynamics (MD) is a powerful simulation technique capable of producing realistic results in a wide spectrum of applications. However, the computational cost of a detailed atomistic interaction model in that paradigm severely limits its applicability beyond extremely small spatial and short temporal scales. Within the family of simulation techniques designed to overcome the limitations of MD, we turn our attention to Dissipative Particle Dynamics (DPD), which allows the study of complex hydrodynamic phenomena in extensive scales. The DPD method was introduced in 1990s as a novel scheme for mesoscopic simulations of complex fluids [111, 112]. In DPD simulations, the particles represent clusters of molecules that interact via conservative (non-dissipative), dissipative and fluctuating stochastic forces. Because the effective interactions between clusters of molecules are much softer than the interactions between individual molecules, much longer time steps can be taken relative to MD simulations. This approach is ultimately based on the Langevin equation, the stochastic differential equation describing Brownian motion accounting for the omitted degrees of freedom by a viscous force and a noise term.

The original DPD model tracks the equation of motion of the particles:

49 $$\begin{aligned} m_i \frac{\partial \mathbf {v}_i}{\partial t} = \mathbf {F}_i \;\;, \end{aligned}$$

where $m_i$, $\mathbf {r}_i$ and $\mathbf {v}_i = \frac{\partial \mathbf {r}_i}{\partial t}$ are the mass, position and velocity of particle *i*, respectively. The total force, $\mathbf {F}_i$, acting on each particle consists of three parts:

50 $$\begin{aligned} \mathbf {F}_i = \sum _{j \ne i} \left( \mathbf {F}_{ij}^\text{C} + \mathbf {F}_{ij}^\text{D} + \mathbf {F}_{ij}^\text{S}\right) , \end{aligned}$$

where $\mathbf {F}_{ij}^\text{C}$, $\mathbf {F}_{ij}^\text{D}$ and $\mathbf {F}_{ij}^\text{S}$ represent the conservative, dissipative and stochastic forces between particles *i* and *j*, respectively. The conservative force depends on the distance between particles *i* and *j*, $r_{ij}$ and is directed along the unit vector of their separation, $\hat{\mathbf {\mathbf {r}}}_{ij}$:

51 $$\begin{aligned} \mathbf {F}_{ij}^\text{C} = f^\text{C} \left( r_{ij}\right) \hat{\mathbf {\mathbf {r}}}_{ij} \end{aligned}$$

where $f^\text{C} \left( r_{ij}\right)$ is a non-negative (i.e. neutral or repulsive) scalar function determining the form of the conservative interactions, depending on the particular system of interest. In literature it is frequently implemented as a soft repulsion of the form:

52 $$\begin{aligned} f^\text{C}\left( r_{ij}\right) = {\left\{ \begin{array}{ll} \alpha _{ij} \left( 1- \frac{r_{ij}}{r_\text{c}}\right) & \quad {} r_{ij} \le r_\text{c} \\ 0 & \quad {} r_{ij}> r_\text{c} \end{array}\right. } \end{aligned}$$

where $\alpha _{ij}$ is a parameter determining the maximum repulsion between the particles and $r_\text{c}$ is a cut-off distance.

The dissipative force, $\mathbf {F}_{ij}^\text{D}$, represents the effect of viscosity and depends on the relative positions and velocities of the particles. The form usually used for this interaction in DPD simulations is [113]:

53 $$\begin{aligned} \mathbf {F}_{ij}^\text{D} = - \gamma w^\text{D} \left( r_{ij}\right) \left( \hat{\mathbf {\mathbf {r}}}_{ij} \cdot \mathbf {v}_{ij}\right) \hat{\mathbf {\mathbf {r}}}_{ij} \end{aligned}$$

where $\gamma$ is a friction coefficient, $\mathbf {v}_{ij} = \mathbf {v}_i - \mathbf {v}_j$ and $w^\text{D}\left( r_{ij}\right)$ is a distance-dependent weighting function. The fluctuating random force depends on the relative positions of the particles, and is defined as:

54 $$\begin{aligned} \mathbf {F}_{ij}^\text{S} = \sigma w^\text{S}\left( r_{ij}\right) \xi _i \hat{\mathbf {\mathbf {r}}}_{ij}\;\;, \end{aligned}$$

with $\sigma$ being a coefficient, $w^\text{S}\left( r_{ij}\right)$ is a distance-dependent weighting function and $\xi _i$ is a random variable sampled from a Gaussian distribution with zero mean and unit variance. It should be noted that both the dissipative and the random force act along the particle separation vector and therefore conserve linear and angular momentum. Also, the resulting model fluids are Galilean invariant because the particle–particle interactions depend only on relative positions and velocities. The fluctuating stochastic force, $\mathbf {F}_{ij}^\text{S}$, heats up the system, whereas the dissipative force, $\mathbf {F}_{ij}^\text{D}$, reduces the relative velocity of the particles, thus removing kinetic energy and cooling down the system. Therefore, the stochastic and dissipative forces act together to maintain an essentially constant temperature which fluctuates around the nominal temperature of the simulation, *T*. Dissipative particle dynamics simulations can be thought of as thermostatted molecular dynamics simulations with soft particle–particle interactions.

Despite qualitative observations, there was no theoretical justification that DPD produces the correct hydrodynamic behavior until Español and Warren [114] formulated the Fokker-Planck equation for studying the equilibrium properties of the stochastic differential equation describing DPD. Later, Español [115] derived the macroscopic hydrodynamic variables starting from the microscopic description. In order to recover the proper thermodynamic equilibrium for a DPD fluid at a temperature *T*, the coefficients and the weighting functions for the dissipative and random forces should be related by:

55 $$\begin{aligned} w^\text{D} \left( r_{ij}\right) = \left[ w^\text{S} \left( r_{ij}\right) \right] ^2 \end{aligned}$$

and

56 $$\begin{aligned} \gamma = \frac{\sigma ^2}{2 k_\text{B}T} \end{aligned}$$

as required by the fluctuation-dissipation theorem. All interaction energies are expressed in units of $k_\text{B}T$, which is usually assigned a value of unity. One straightforward and commonly used choice is:

57 $$\begin{aligned} w^\text{D}\left( r_{ij}\right) = \left[ w^\text{S} \left( r_{ij}\right) \right] ^2 = {\left\{ \begin{array}{ll} \left( 1 - \frac{r_{ij}}{r_\text{c}}\right) ^s &{} r < r_\text{c} \\ 0 &{} r \ge r_\text{c} \end{array}\right. } \end{aligned}$$

where $r_\text{c}$ is the cut-off distance of the the dissipative and the random force. In conventional DPD formulation, it usually takes the same value as the cut-off distance of the conservative force but it can vary in order to modify the dynamic properties in DPD simulations. For conventional DPD simulations, the exponent of the weighting function, *s*, is set equal to 2 with $w^\text{D}$ and its gradient being continuous at $r_{ij}/r_\text{c}=1$.

Summarizing, Español and Warren [114] established a sound theoretical basis for DPD and Groot and Warren [116] obtained parameter ranges to achieve a satisfactory compromise between speed, stability, rate of temperature equilibration, and compressibility. Unlike traditional DPD methods using conservative pairwise forces between particles, the multi-body DPD model presented by Pagonabarraga and Frenkel [117] assumes that the conservative force depends on the instantaneous local particle density, which in turn depends on the positions of many neighboring particles. As far as the integration of the DPD equations of motion is concerned, Pagonabarraga et al. [118] proposed a leap-frog scheme which was self-consistent and satisfied a form of microscopic reversibility. Thus, the correct equilibrium properties could be recovered from trajectories generated with that algorithm.

### Monte Carlo

The Monte Carlo (MC) method is a statistical approach for finding approximate solutions to problems by means of random sampling. In addition to molecular simulations and physics, it is widely applied in other natural sciences, mathematics, engineering and social sciences [119]. The earliest treatments in the subject, such as this by Babier [120], were made in connection with the “Buffon’s needle problem”.^2^ According to Metropolis [121], the invention of the modern class of MC algorithms is due to Enrico Fermi, when he was studying the properties of the then newly-discovered neutron in 1930. It was further developed during the 1940s by physicists working in the nuclear weapons program of the United States, at the Los Alamos National Laboratory. The technique was given its name by Nicholas Metropolis, in reference to the famous casino in Monaco, considering the use of randomness and the repetitive nature of the sampling process.

In their simplest version, MC simulations of simple fluids are carried out by sampling trial moves for the molecules from a uniform distribution. For example, in a canonical (*NVT*) ensemble simulation, a molecule is chosen at random, and then displaced, also randomly to a new position. The trial move is accepted or rejected according to an importance sampling scheme [93, 122, 123]. A frequently used importance sampling algorithm is the Metropolis algorithm, originally derived for the specific case of the Boltzmann distribution [122] and later generalized to other distributions [124] which need not to be analytical (e.g. the force-bias method of Pangali et al. [123] provides a classical example of such algorithms).

The probability of accepting a move, $P_\text{accept}$, of the form:

58 $$\begin{aligned} P_\text{accept} = \min {\left[ 1, \frac{P\left( \mathscr {O} \vert \mathscr {N}\right) P\left( \mathscr {N}\right) }{P\left( \mathscr {N} \vert \mathscr {O}\right) P\left( \mathscr {O}\right) } \right] } \end{aligned}$$

will asymptotically sample the configuration space according to a probability *P*. In Eq. (58), $P_\text{accept}$ is the probability with which trial moves are accepted or rejected, $P\left( \mathscr {N} \vert \mathscr {O}\right)$ is the transition probability of making a trial move from state $\mathscr {O}$ to state $\mathscr {N}$, and $P\left( \mathscr {O}\right)$ is the probability of being at state $\mathscr {O}$. This means that, at equilibrium, the average number of accepted trial moves that result in the system leaving state $\mathscr {O}$ must be exactly equal to the number of accepted trial moves from all other states $\mathscr {N}$ to the state $\mathscr {O}$. This is a looser statement of the *detailed balance condition*, reflected in Eq. (58), that at equilibrium the average number of accepted moves from $\mathscr {O}$ to any other state $\mathscr {N}$ should be exactly canceled by the number of reverse moves.

In the original Metropolis scheme [122], the probabilities $P\left( \mathscr {N} \vert \mathscr {O}\right)$ form a symmetric matrix, constructing a Markov chain that has the Boltzmann distribution as its equilibrium distribution. In this case, there is no bias involved in making the move and Eq. (58) reduces to the standard Metropolis acceptance criterion:

59 $$\begin{aligned} P_\text{accept} = \min {\left\{ 1, \exp {\left[ -\frac{\mathscr {V}\left( \mathscr {N}\right) - \mathscr {V}\left( \mathscr {O}\right) }{k_\text{B}T}\right] } \right\} } \end{aligned}$$

The advanced MC methods are based on judicious choices of $P \left( \mathscr {N} \vert \mathscr {O}\right)$ [93]. It should be noted that the simulation steps in the MC technique are steps in configuration space and there is no notion of “time” in MC simulations. This is contrast to MD, where the simulation steps are explicit time steps. Moreover, a computational advantage of MC over MD is that only the energy needs to be calculated, not the forces, rendering the Central Processing Unit (CPU) time needed per step smaller than that of an MD simulation.

### Reduced Units

Molecular simulations can conveniently be performed in non-dimensional or reduced units, based on the characteristic physical dimensions of the system under study. Working with reduced units is preferred mainly because they are physically easier to interpret, and the results obtained become applicable to all materials modeled by the same potential. Reduced units are obtained by expressing all quantities of the simulation in terms of selected base units which are characterizing the system, in order to make equations dimensionless. Table 1 presents some reduced quantities. For example, in the case of the Lennard-Jones potential, the particle diameter, $\sigma$, the depth of the potential well, $\varepsilon$, together with the mass of the simulated particles, *m*, provide a meaningful and complete set of base units for simulations.

**Table 1:** Conversion to reduced units for some commonly used quantities with $\varepsilon$, $\sigma$ and *m* as the basis units for energy, length and mass, respectively

Quantity	In reduced units
Energy	$E^* = E / \varepsilon$
Length	$L^* = L / \sigma$
Mass	$M^* = M / m$
Density	$\rho ^* = \sigma ^3 \rho$
Temperature	$T^* = (k_\text{B}T)/\varepsilon$
Force	$F^* = (\sigma F)/\varepsilon$
Pressure	$p^* = (\sigma ^3 p) / \varepsilon$
Time	$t^* = t \sqrt{\varepsilon / (m \sigma ^3)}$

## Structural Predictions

### Chain Dimensions in the Bulk

One of the most important and probably the most fundamental question in the area of PNCs is how the size of the polymer chains is affected by the dispersion of nanoparticles. There has been considerable controversy in the experimental literature over whether nanoparticles cause chain expansion (swelling) [125, 126], contraction, [127] or neither [128–133]. The sign (attractive or repulsive) and strength of the nanoparticle/polymer interactions, the relative dimensions of the chains with respect to the size of the nanoparticle, $R_\text{g}/R_\text{n}$, and the exact state of dispersion, have been identified as the key factors that can account for the aforementioned differences in the structure of the matrix chains.

#### Experimental Findings

Chain conformations in PNCs have been primarily measured by small angle neutron scattering (SANS). These measurements are greatly facilitated by combining deuterated and hydrogenated chains such that the average scattering length density of the polymer blend matches that of the filler. This zero average contrast condition [134], which is hard to achieve, minimizes the scattering due to the nanoparticles. To date, studies which report increases in polymer dimensions, in the case of spherical nanoparticles, invoke the presence of attractive nanoparticle/polymer interactions, combined with $R_\text{g}> R_\text{n}$, and good nanoparticle dispersion [135], to conclude that the nanoparticles behave as a good solvent for the polymer chains. However, even though the existence of a shell containing polymer of reduced mobility is acceptable in nanocomposites composed of strongly interacting particles and polymer, e.g. composed by silica and PMMA, the size of the chains, e.g. in terms of their radius of gyration, $R_\text{g}$, is intrinsically independent of the the volume fraction, $\phi$, (up to $\phi \simeq 0.1$) and the polymer-to-particle size ratio [132]. All other studies on spherical nanoparticles showed little if any changes in polymer $R_\text{g}$, that is where the nanoparticle–polymer intractions are believed to be athermal, or significant nanoparticle aggregation was present, due to unfavorable nanoparticle/polymer interactions [136].

In an early study of a poly(dimethylosiloxane) / polysilicate ($R_\text{n} = 1 \;\text{nm}$) nanocomposite [125], a significant increase of the polymer chain dimensions (reaching 60% expansion at nanoparticle volume fraction, $\phi = 40$%) was observed for $R_\text{g}> R_\text{n}$ and a decrease in polymer dimensions for $R_\text{g} \le R_\text{n}$. Neutron scattering studies of an athermal polystyrene (PS) PNC, indicated swelling of the matrix chains, induced by dispersed tightly cross-linked PS nanoparticles [126]. PS chains around crosslinked PS particles ($R_\text{n} = 2 - 3.6 \;\text{nm}$) were found to be expanded by up to 20 % relative to their unperturbed size, when their unperturbed radius of gyration was comparable to or larger than the radius of the dispersed particles. More recent studies of PS/silica nanocomposite [128, 129, 131] for $R_\text{g}/R_\text{n} = 2 - 4,$ [131] and poly(ethylene-propylene)/silica nanocomposites, [127] found no perturbation of the matrix chain dimensions.

We may attribute the qualitatively different trends deduced by different experimental studies to several factors, including, but not limited to the following: (a) most of the polymers used exhibit significant polydispersity, (b) particle dispersion/agglomeration cannot be adequately quantified, (c) the molecular weight of the isotopic polymers blended with the filler was quite different in at least one case. The compound effect of these factors can result in significant uncertainty in the chain dimensions measured. Molecular simulations can shed some light on the role of nanoparticles on chain dimensions, especially in regimes where it is hard to conduct experiments.

#### Insight Obtained from Simulations

From the point of view of molecular simulations, there also exists controversy as to whether the incorporation of nanoparticles in a polymer melt causes polymer chains to expand, remain unaltered or reduce [137–139] their dimensions compared to their size in the bulk material. To date, all studies have indicated that, irrespectively of the absolute dimensions of the chains in the interparticle region, these retain their unperturbed Gaussian scaling. This is a striking feature, resembling the scaling behavior of chains in thin films [140, 141], where chain conformations parallel to the surface assume their unperturbed values even for film thicknesses $< R_\text{g}$. Most of the simulation works have addressed polymer dimensions in nanocomposites below the percolation threshold ($\phi _\text{c} = 31 \%$ [142]), except the early works of Vacatello [137–139, 143] that were implemented at constant density and spatially frozen nanoparticles.

**Fig. 1:**
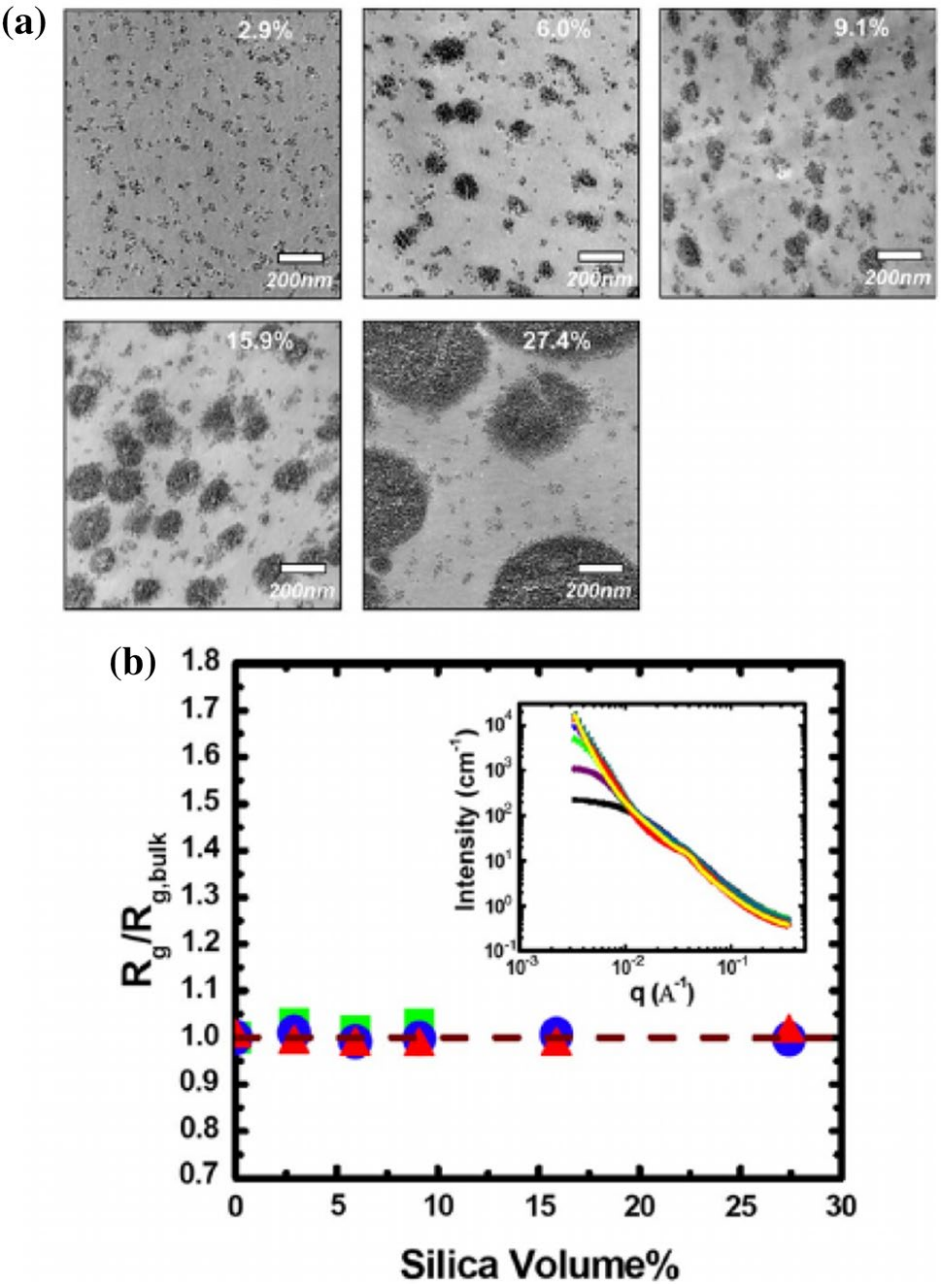
**a** Transmission electron microscopy (TEM) micrographs of nanocomposites formed from PS of 250 kg/mol molecular weight and indicating % vol loading of silica in each sample. **b** Ratio of the radius of gyration of the PS chains in the presence of particles to their corresponding value in the pure blend for 90 kg/mol PS (*green squares*), 250 kg/mol PS (*blue circles*) and 620 kg/mol PS (*red triangles*) as functions of the silica volume fraction. In the* inset* to the figure, a plot of small angle neutron scattering intensities in absolute units as a function of *q* for the 250 kg/mol PS nanocomposites. The interested reader can refer to [128] for more details. (Color figure online) (Reprinted figure with permission from [128]. Copyright 2007 by the American Physical Society)

Sen et al. [128] employed polymer reference interaction site model (PRISM) [144, 145] calculations in order to interpret small angle neutron scattering findings on polystyrene loaded with spherical silica nanoparticles under contrast-matched conditions. They considered blends with 66 wt% hPS and 34% dPS, which almost contrast match the silica. Nanocomposites with 0, 2.9, 6.1 and 9.1% vol silica were prepared for each molecular weight and 15.9 and 27.4 vol% for the higher molecular weights considered. However, in their experiments, as in earlier studies [146, 147], the particles were imperfectly mixed with the polymeric matrix, with particles being surrounded by “voids”, especially at large filler contents. In parallel, the PRISM theory was applied, by modeling the fillers as hard spheres and polymers as freely jointed chains with a realistic persistence length. Polymer–polymer and particle–particle interactions were taken to be hard-core, while monomers and filler interact via an exponentially decreasing attraction over a predefined spatial range. From the experimental point of view (Fig. 1), the low-*q* intensity increases dramatically with increasing silica content, especially for loadings ≤10 vol%, implying that the matrix is not totally contrast matched to the filler (unsurprising in light of voids surrounding particles [146]). However, the scaling and dimensions of the polymer chains can be obtained from the high-*q* intensity which is expected to be independent of the filler structure [146]. Their results (Fig. 1) showed that chain conformations follow unperturbed Gaussian statistics independent of chain molecular weight and filler composition. Liquid state theory calculations were consistent with this conclusion and also predicted filler-induced modification of interchain polymer correlations which had a distinctive scattering signature that was in nearly quantitative agreement with the experimental observations. The chain $R_\text{g}$ varied from ~8 nm (90 kg/mol) to 22 nm (620 kg/mol), bracketing the nanoparticle diameter (~14 nm), suggesting that the ratio of the particle size to $R_\text{g}$ was not an important variable in that context.

**Fig. 2:**
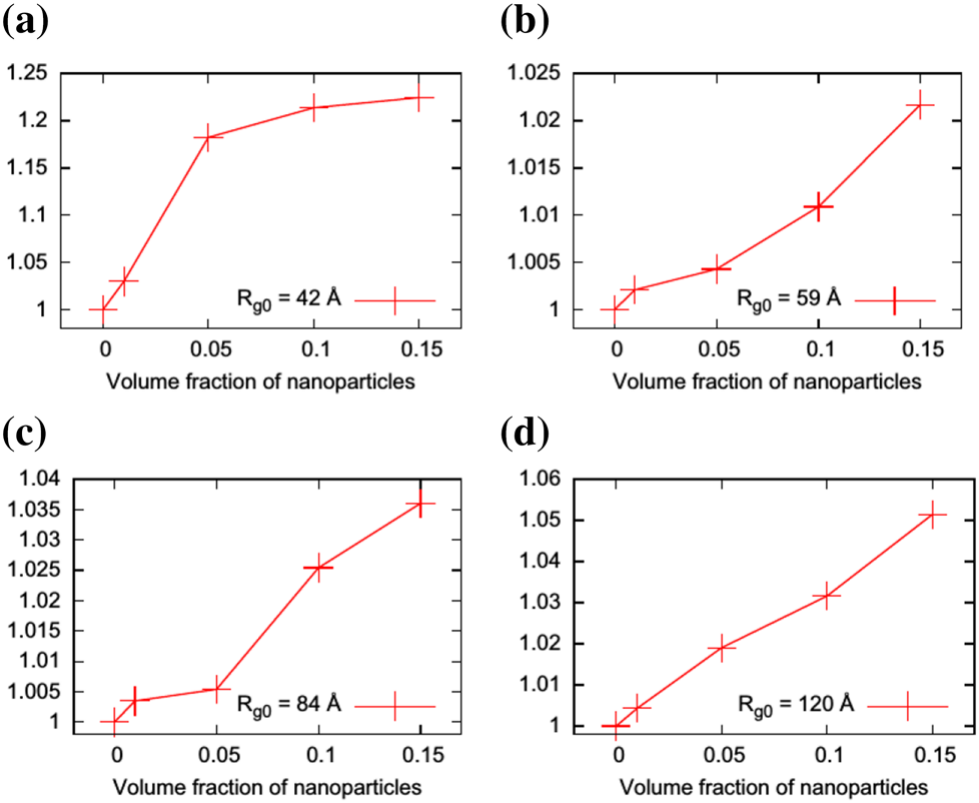
Radius of gyration of polystyrene chains in melts filled with tightly cross-linked PS nanoparticles of radius $R_\text{n} = 3.6\;\text{nm}$, normalized by its value in the bulk, $R_\text{g,0}$, as a function of the particle volume fraction. The corresponding molecular weights are 23 (**a**), 47 (**b**), 93 (**c**) and 187 (**d**) kg/mol, respectively. (Reprinted from [148] with permission from Elsevier.)

The structure of a polystyrene matrix filled with tightly cross-linked polystyrene nanoparticles, forming an athermal nanocomposite system, has been investigated by means of a Monte Carlo sampling formalism by Vogiatzis et al. [148]. Although the level of description is coarse-grained (e.g., employing freely jointed chains to represent the matrix), the approach developed aims at predicting the behavior of a nanocomposite with specific chemistry quantitatively, in contrast to previous coarse-grained simulations. A main characteristic of the method was that it treats polymer–polymer and polymer–particle interactions in a different manner: the former are accounted for through a suitable functional of the local polymer density, while the latter are described directly by an explicit interaction potential. The simulation methodology was parameterized in a bottom-up fashion in order to mimic the experimental studies. Many particle systems, with volume fractions up to 15 vol%, were simulated. The positions of the nanoparticles were held constant in the course of the simulation, while polymeric chains were allowed to equilibrate via a combination of MC moves. The generation of many independent initial configurations compensated for the immobility of the particles along the simulation. The values of the radius of gyration $R_\text{g}$, relative to the value for the pure polymer melt $R_{\text{g}0},$ are shown in Fig. 2 as a function of the nanoparticle volume fraction for the four different chain molecular weights used in that work (23, 47, 93 and 187 kg/mol). In general, an expansion of polymeric chains with increasing nanoparticle volume fraction can be observed for all chain lengths. This expansion is maximal for 23 kg/mol, where the unperturbed radius of gyration $R_{\text{g},0} \simeq 42$ Å is comparable to the radius of the nanoparticle, $R_\text{n} = 36$ Å. It seems that there is a tendency of chains to swell when their dimension is equal to or approaches the dimension of the nanoparticle. This observation is in very good quantitative agreement with experimental data reported for the same system [126]. In all other cases, the swelling due to the presence of the nanoparticles could hardly be discerned.

**Fig. 3:**
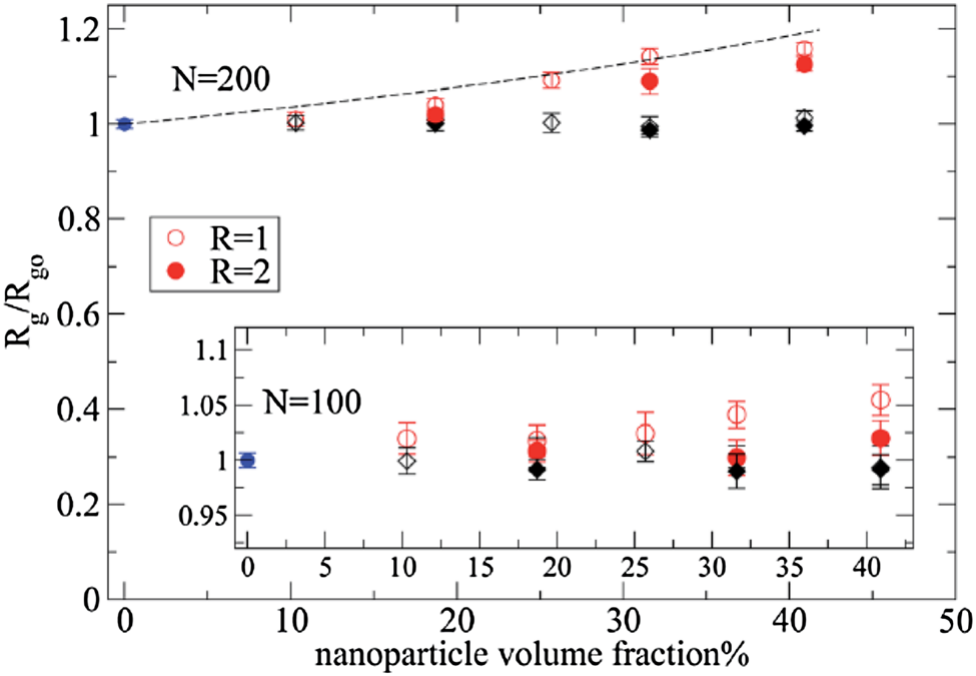
Radius of gyration of polymers in melt with nanoparticles of radius $R_\text{n} = 1,\;2$ normalized with its value in the bulk for polymer chains of $N = 200$ and $N=100$ (*inset*) repeat units (monomers): (i) polymer melt (*blue filled circles*), (ii) nanocomposite: attractive monomer-nanoparticle ($R_\text{n} = 2$) interactions (*red filled circles*), (iii) nanocomposite: repulsive monomer-nanoparticle ($R_\text{n}=2$) interactions (*black filled diamonds*), (iv) nanocomposite: attractive monomer-nanoparticle ($R_\text{n}=1$) interactions (*red open circles*), (v) nanocomposite: repulsive monomer-nanoparticle ($R_\text{n}=1$) interactions (*black open diamonds*). The* black dashed line* shows $R_\text{g}/R_\text{g,0} \propto \left( 1 - \phi \right) ^{-1/3}.$ (Color figure online) (Reprinted from [135]—Published by The Royal Society of Chemistry.)

Karatrantos et al. [135] have investigated the effect of various spherical nanoparticles on chain dimensions in polymer melts for high nanoparticle loading which was larger than the percolation threshold, using coarse-grained molecular dynamics simulations of the Kremer-Grest model [149]. Their results, presented in Fig. 3, revealed different behavior of the polymer chains in the presence of repulsive or attractive particles. In nanocomposites containing repulsive nanoparticles (black symbols), the polymer dimensions were not altered by the particle loading. These authors reported that the polymers were phase separated from the repulsive nanoparticles (of $R_\text{n} =2$) in the nanocomposites, thus, there were no changes in the radius of gyration values. On the contrary, in the nanocomposites containing attractive nanoparticles, the overall polymer dimensions increased dramatically at high particle loading. In particular, the magnitude of expansion of polymer dimensions was larger for polymers with $N = 200$ following qualitatively the experimental data [125, 150]. The relation $R_\text{g}/ R_{\text{g},0} = \left( 1-\phi \right) ^{-1/3}$, included in Fig. 3 was proposed by Frischknecht et al. [151] for predicting the polymer expansion due to the excluded volume introduced by the nanoparticles, assuming no change in density on mixing. Finally, Karatrantos et al. [135] reported that polymer chains, in all cases considered, did not depart from Gaussian statistics.

**Fig. 4:**
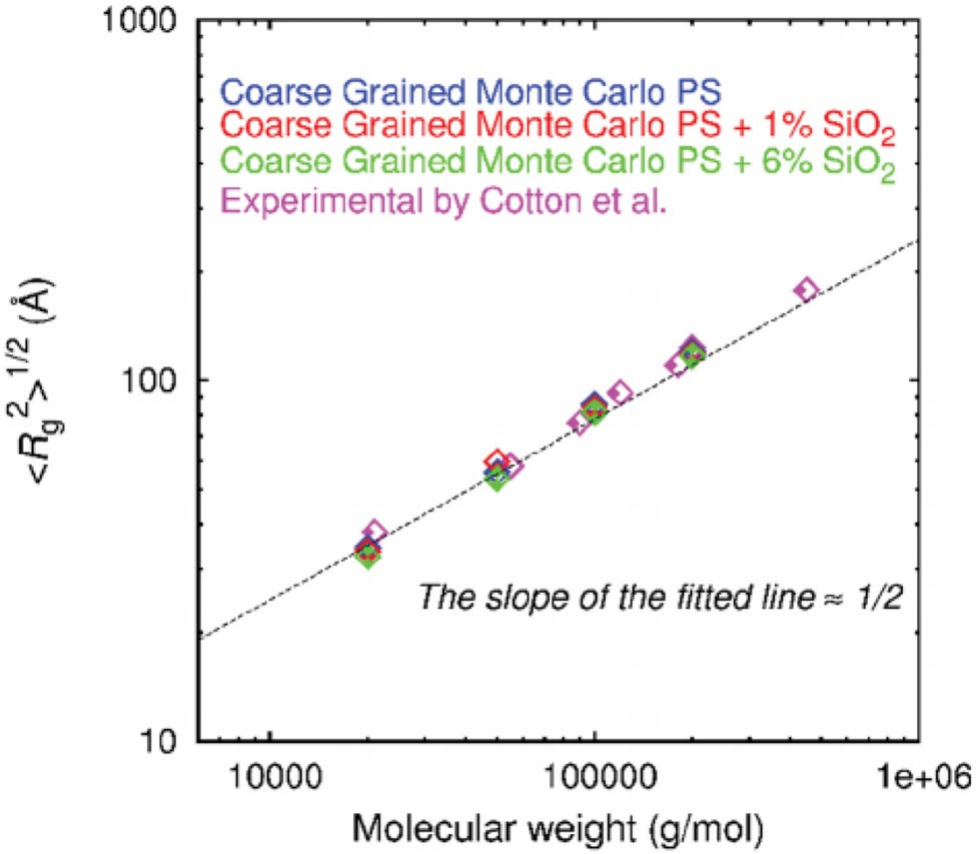
Root mean squared radius of gyration of the coarse-grained chains of neat and nanocomposite polystyrene systems as a function of molar mass, *M*, in the melt at 500 K (*red*,* green* and* magenta rhomboid symbols* ). The systems contain one nanoparticle of diameter 3 nm ($\phi \simeq$ 1%) and 6 nm ($\phi \simeq$ 6%). Neutron scattering measurements [152] for high molar mass PS are also included (*blue rhomboid symbols*). The* black dotted line* is a linear least-squares fit to a relation of the form $\left\langle R_\text{g}^2 \right\rangle ^{1/2} \propto M^{1/2}$ in the loglog coordinates of the plot. (Color figure online) (Reproduced from Ref. [153] with permission from The Royal Society of Chemistry.)

Mathioudakis et al. [153] applied a hierarchical simulation approach in order to study the behavior of PS–SiO_2_ nanocomposites. Two interconnected levels of representation were employed. (a) A coarse-grained one [154], wherein each polystyrene repeat unit was mapped into a single “superatom” and each silica nanoparticle into a sphere. The smoother effective potential energy hypersurface of the coarse-grained representation permitted its equilibration at all length scales by using powerful connectivity-altering Monte Carlo algorithms [155]. (b) A united atom representation, wherein polymer chains were represented by a united-atom model and a silica nanoparticle was represented in full atomistic detail. Coarse-graining and reverse-mapping between the two levels of representation was accomplished in a manner that preserved tacticity and respected the detailed conformational distribution of chains [156]. At the coarser level, these authors estimated the root-mean-square radius of gyration $\left\langle R_\text{g}^2 \right\rangle ^{1/2}$ as a function of the molecular weight for neat and nanocomposite polystyrene systems. Their results are presented in Fig. 4 along with neutron scattering results for bulk monodisperse PS [152] from 21 to 1100 kg/mol. As far as the nanocomposite polystyrene systems are concerned, the presence of the nanoparticles affected the root- mean-square radius of gyration only slightly.

### Polymer Structure in the Vicinity of the Filler Particles

#### Experimental Findings

SANS measurements show a clear scattering signature of a polymer bound layer around the particles, which arises due to a scattering length density different from the bulk polymer matrix material, either due to H or D enrichment or a modification of the polymer density in the bound layer compared to the surrounding polymer matrix [132, 133]. The measurements of Jouault et al. [133] revealed that the bound layer is independent of the particles’ volume fraction. Then, as observed by Jiang et al. [157], the bound layer volume fraction is larger at the surface (that region being mostly composed of loops) and decreases at larger distances as the bound layer becomes more diffuse due to the contribution from the tails. One can estimate the thickness of the bound polymer layer around 2 nm. However, this thickness value is a simplification because it does not completely describe the complex chain behavior, some aspects of which will be analyzed below.

#### Insight Obtained from Simulations

**Fig. 5:**
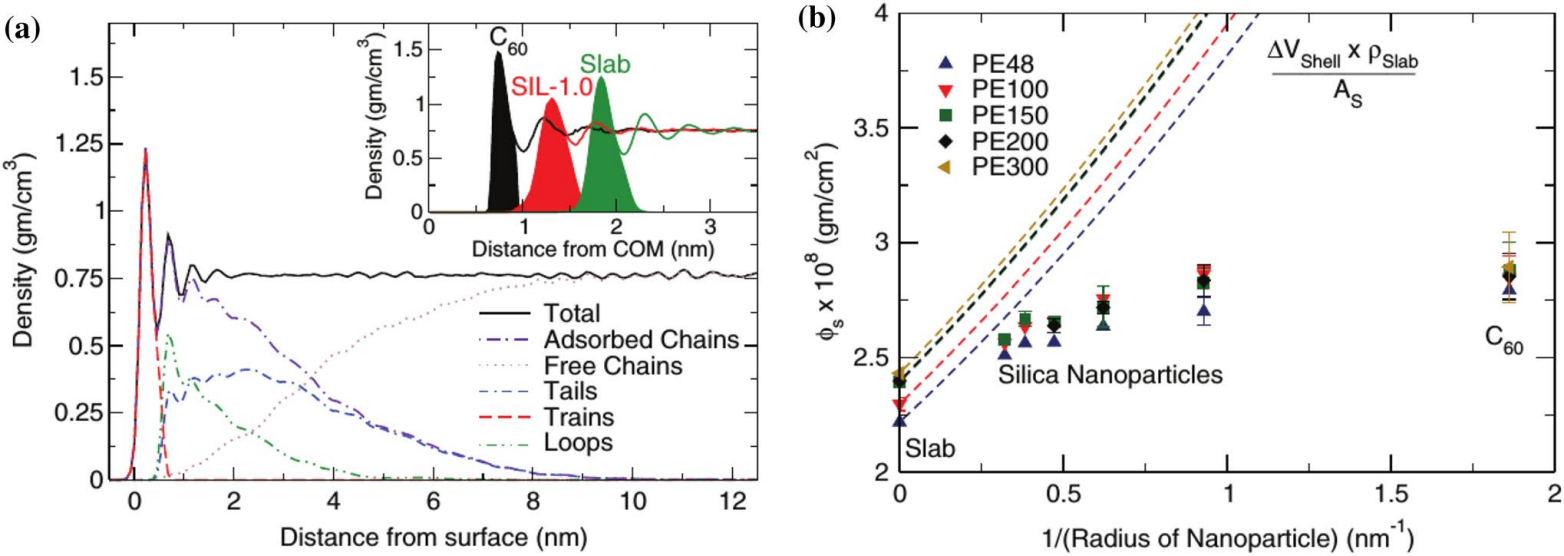
**a** Density distribution of a polyethylene melt as a function of distance from the surface of a filler (graphite slab, silica nanoparticle or fullerene C$_{60}$). The decomposition into tails, trains and loops is carried out following Scheutjens and Fleer [164]. The inset provides profiles for selected systems.** b** Surface concentration together with predictions based upon geometrical arguments for ideal spheres and surface monomer density in the proximity of silica slabs. (Reprinted from [159], with the permission of AIP Publishing.)

The local density of the polymer in the proximity of the surface of a filler is often employed as an indication of the strength of polymer–surface interactions and a decrease of the first peak of the radial density distribution is expected with curvature [158]. At this point we resort to the detailed analysis of Pandey and Doxastakis [159] concerning a polyethylene layer close to a filler surface (Fig. 5). These authors coupled the application of preferential sampling techniques [160] with connectivity-altering Monte Carlo algorithms [161, 162] in order to explore the configurational characteristics of a polyethylene melt in proximity to a silica surface or around a nanoparticles and the changes induced by high curvature when the particle radius is comparable to the polymer Kuhn segment length.

The inset to Fig. 5 shows that indeed as we move from a flat surface to a smaller nanoparticle a decrease is observed with the exception of the fullerene where a significantly higher density is found. To investigate further the concentration of adsorbed monomers, these authors followed the use of a simple distance criterion (adsorbed polymer chains have an atom within 0.6 nm of an atom of the surface; introduced by Daoulas et al. [163]) to decompose polymer segments according to the Scheutjens-Fleer theory [164] into trains, tails and loops. *Tails* are the segments which are hinged to the surface at one end while the other end is dangling freely into the bulk polymer. *Train* segments consist of monomers consecutively adsorbed on the surface. The *loop* segments are constituted by the monomers in-between two train segments, which are not adsorbed on the surface. Figure 5 exhibits three regimes for adsorbed chains: a first layer of adsorbed monomers constituting train segments, a second layer where a decay is dominated by a decrease of loop segments while tail density is constant and a third layer where tail segments extend in the bulk melt. As shown in the inset to Fig. 5(a) the area under the first peak broadens as we move to smaller particles.

An interesting feature of the interfacial systems to study is the number of monomers that are in contact with the surface. To this end, Pandey and Doxastakis [159], defined a surface concentration by integrating the density profile of train segments:

60 $$\begin{aligned} \Phi _\text{s} = \frac{\int _{R_\text{n}}^\infty \rho _\text{train}\left( r\right) 4 \pi r^2 \text{d}r}{A}\;\;, \end{aligned}$$

where $R_\text{n}$ is the radius of the nanoparticle, $\rho _\text{train}$ is the density profile of train segments, and *A* is the accessible surface area to the polymer. If we assume that nanoparticles are spheres surrounded by a constant density of polymer, $\rho _0$, in a layer of $\Delta r$ thickness, the surface concentration is given by:

61 $$\begin{aligned} \Phi _\text{s} = \frac{\left( r+ \Delta r\right) ^3 - r^3}{3 r^2} \rho _0\;\;, \end{aligned}$$

where a constant density is multiplied with the ratio of the volume of a spherical shell representing the first adsorbed monolayer to the surface of the sphere. The geometric predictions following the above line of reasoning, are shown for different chain lengths by the dashed lines in Fig. 5(b). It is apparent that a modest increase and ultimate leveling off of the surface concentration with decreasing nanoparticle radius is observed in sharp contrast to the estimations based on the geometric arguments, which predict a continuous increase. The lower than anticipated increase of surface concentration with curvature suggests that collective properties beyond the enthalpic interactions appear to play a crucial role on surface concentration. At the extreme limit where particles are comparable to the polymer Kuhn segment length, curvature penalizes the formation of long train segments. As a result, an increased number of shorter contacts belonging to different chains were made, competing with the anticipated decrease of the bound layer thickness with particle size if polymer adsorbed per unit area remained constant.

**Fig. 6:**
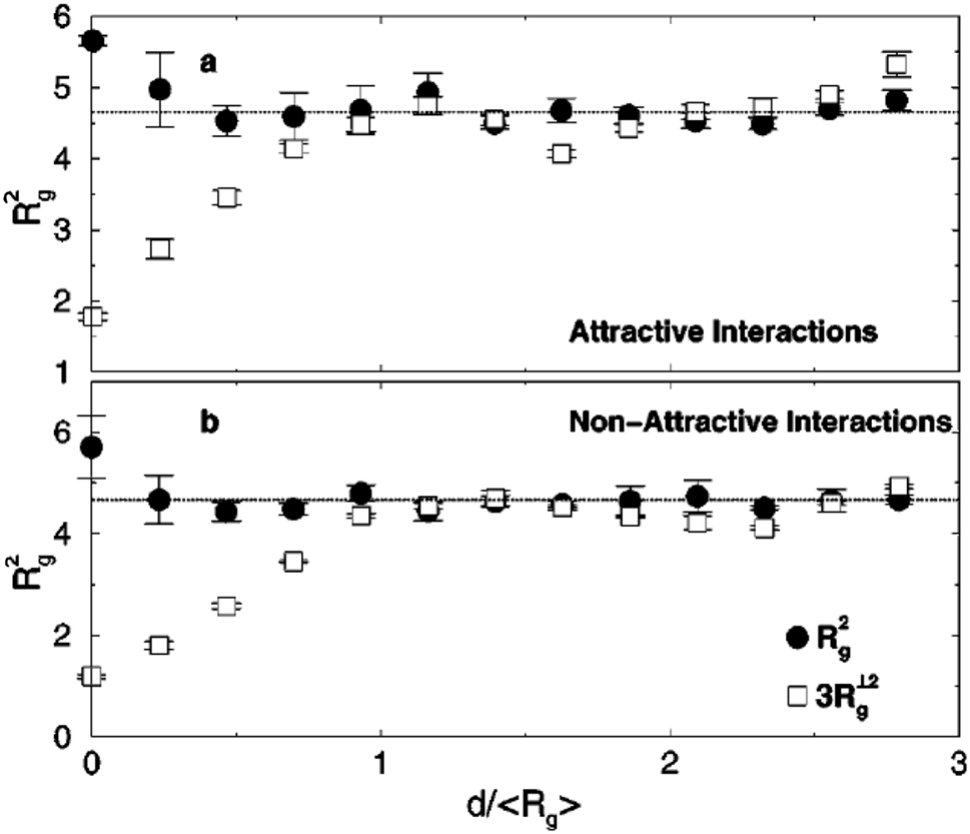
Radius of gyration, $R_\text{g}$, in reduced Lennard-Jones units, of the polymer chains as a function of the distance $d/\left\langle R_\text{g} \right\rangle$ of the center of mass of a chain from the filler surface (*d* is normalized by the average $\left\langle R_\text{g} \right\rangle$ of all chains). Results are presented for **a** attractive and **b** nonattractive interactions. The component of $R_\text{g}$ perpendicular to the surface, $R_\text{g}^{\perp }$ is resolved. The* dotted line* shows $\left\langle R_\text{g}^2 \right\rangle$ for the pure system. The increase of $R_\text{g}$, coupled with the decrease of $R_\text{g}^\perp$, indicates that the chains become increasingly elongated and flattened as the surface of the particle is approached. (Reprinted figure with permission from [165]. Copyright 2001 by the American Physical Society)

Starr et al. [165] carried out extensive molecular dynamics simulations of a single nanoscopic filler particle surrounded by a dense polymer melt. The polymers were modeled as chains of monomers connected via a finitely extensible nonlinear elastic (FENE) anharmonic potential and interacting via a Lennard-Jones potential. That type of “coarse-grained” model is frequently used to study general trends of polymer systems but does not provide information for a specific polymer. The filler particle shape was icosahedral with interaction sites assigned at the vertices, at four equidistant sites along each edge, and at six symmetric sites on the interior of each face of the icosahedron. These authors considered a filler particle with an excluded volume interaction only, as well as one with excluded volume plus attractive interactions in the dilute nanoparticle regime (where bulk chain dimensions are unlikely to be affected by the confinement between nanoparticles). By focusing on the dependence of $R_\text{g}$ on the distance *d* from the filler surface, these authors were among the first to report a change in the overall polymer structure near the surface. In Fig. 6, $R_\text{g}^2$, as well as the radial component of from the filler center $R_\text{g}^{\perp 2}$ (approximately the component perpendicular to the filler surface) for both attractive and nonattractive polymer–filler interactions at one temperature. A striking feature of Fig. 6 is that $R_\text{g}^2$ increases by about 30% on approaching the filler surface, while at the same time $R_\text{g}^{\perp 2}$ decreases by more than a factor of 2 for both (attractive and neutral) systems. The combination of these results indicates that the chains become slightly elongated near the surface and flatten significantly. The range of the flattening effect roughly spans a distance of an unperturbed radius of gyration, $R_\text{g,0}$, from the surface and depends only weakly on the simulation temperature, *T*. Moreover, the chains retain a Gaussian structure near the surface.

**Fig. 7:**
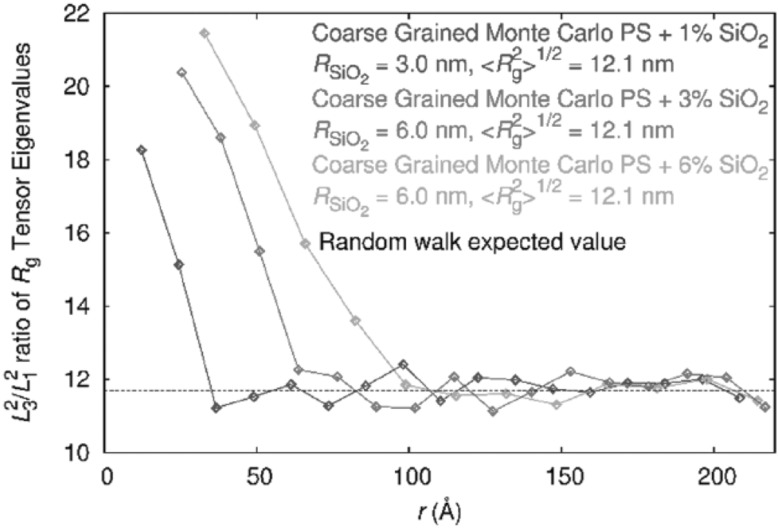
Ratio $L_3^2/L_1^2$ of the largest to the smallest eigenvalue of the radius of gyration tensor of the chain as a function of the distance of the center of mass of the chain from the center of mass of a silica nanoparticle. The systems consist of chains of molar mass $M = 208$ kg/mol and one nanoparticle of radius either 3 nm (silica volume fraction $\phi _{\text {SiO}_{2}}$ = 1%) or 6 nm ($\phi _{\text {SiO}_{2}}$= 3 and $\phi _{\text {SiO}_{2}}$ = 6%). The expected value from the random walk model for bulk PS is also included (*black dotted line*) [148]. (Reproduced from Ref. [153] with permission from The Royal Society of Chemistry.)

Mathioudakis et al. [153] studied the shape of chains in the presence of a silica nanoparticle by employing coarse-grained MC simulations. These authors analyzed the eigenvalues of the the radius of gyration tensor, which serve as a measure for characterizing the shape of the polymer chains. In the polymer melt, the intrinsic shape of chains is that of a flattened ellipsoid or soap bar [166]. Following ref. [166], Mathioudakis et al. diagonalized the instantaneous radius of gyration tensor of every chain to determine the eigenvalues $L_3^2 \ge L_2^2 \ge L_1^2$ (squared lengths of the principal semiaxes of the ellipsoid representing the segment cloud of the chain) and the corresponding eigenvectors (directions of the principal semiaxes). The three semiaxes are generally unequal. The sum $L_1^2 + L_2^2 + L_3^2$ equals the squared radius of gyration of the chain. These authors observed that, when the distance of the center of mass of the chain from the center of the nanoparticle was shorter than the mean size of the chain, the chains expanded along their principal semiaxis, $L_3$. That led to an increase of the radius of gyration, $R_\text{g}^2 = L_1^2 + L_2^2 + L_3^2$ near the nanoparticle. The deformation of the molecules was smaller for chains whose dimensions exceed by far the radius of the nanoparticle. Far from the surface of the nanoparticle, the sum of the squares of the principal semiaxes (sum of the eigenvalues of the radius of gyration tensor) reaches the bulk average value of the squared radius of gyration of PS, since the molecules were not affected by the presence of the nanoparticle. These results are shown in Fig. 7. Changes in the intrinsic shape of chains were quantified as a function of distance of the center of mass of the chain from the center of mass of the silica particle by calculating the ratio of largest to smallest eigenvalue of the radius of gyration tensor. This local anisotropy of the chains as a function of distance from the centre of mass of the nanoparticle is also shown in Fig. 7.

**Fig. 8:**
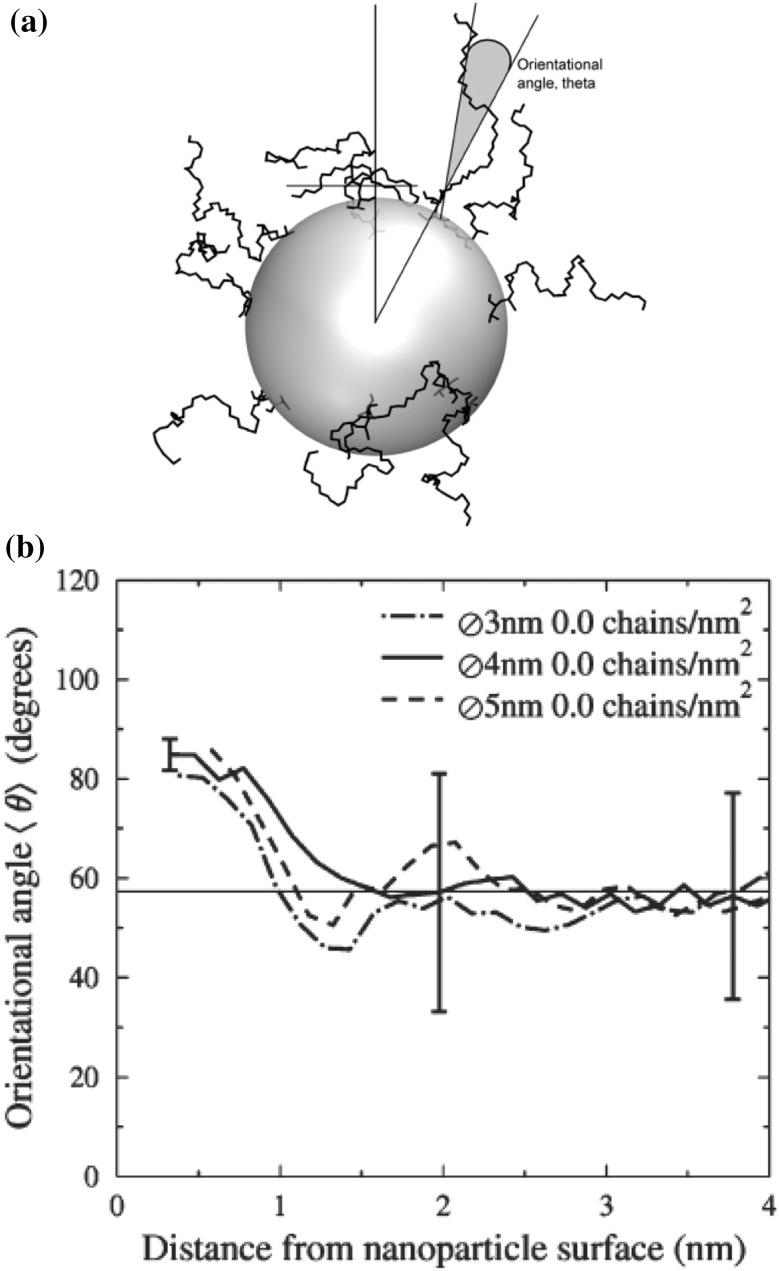
**a** Schematic representation of the orientational angle $\theta$ between the longest axis of the radius of gyration tensor and the surface normal (simulation snapshot). (Reprinted with permission from Ref. [158]. Copyright (2011) American Chemical Society.)** b** PS Chain orientation as a function of the chain (center-of-mass) distance from the silica nanoparticle surface. The considered nanoparticle diameters were 3, 4, and 5 nm. The orientation angle is calculated between the longest axis of the squared radius of gyration tensor (eigenvector corresponding to its largest eigenvalue, disregarding the sign) and the surface normal (c.f. (**a**)). The* horizontal line* at 57.3 marks the average orientational angle for a random distribution. (Reprinted with permission from Ref. [158]. Copyright (2011) American Chemical Society.)

The presence of the filler surface also influences the orientation of the chains. Ndoro et al. [158] studied the distance dependence of the angle between the longest axis of the radius of gyration tensor and the surface normal of bare silica nanoparticle. Their results are presented in Fig. 8. The observation that the free polymer chains generally prefer to align tangentially to the ungrafted surface is in agreement with conclusions from other researchers [148, 167]. In their coarse-grained model using Monte Carlo simulations, Vogiatzis et al. [148] studied the orientational angles of local chain segments. They also concluded that chain segments in the vicinity of the nanoparticle surface were structured and oriented tangentially to the interface.

**Fig. 9:**
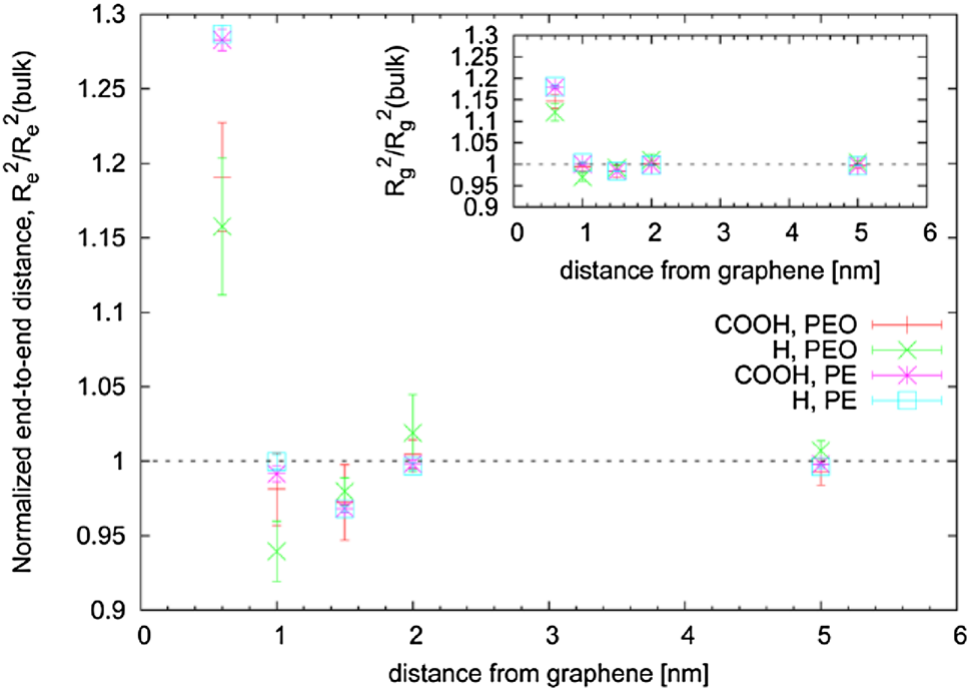
Mean square end-to-end distance of polymer chains located at different layers parallel to graphene, normalized by the same quantity measured in bulk. In the* inset* to the figure, the normalized radius of gyration is plotted for the same set of data. The first layer extends within distances 0.0 and 0.6 nm from the graphene sheet, the second between 0.6 and 1.0 nm, the third between 1.0 and 1.5 nm, the fourth between 1.5 and 2.0 nm and the last one, fifth, between 2.0 and approximately half the edge length of the simulation box, 5.0 nm. (Reprinted with permission from Ref. [168]. Copyright (2011) American Chemical Society.)

Bačová et al. [168] conducted atomistic molecular dynamics simulations of graphene-based polymer nanocomposites composed of hydrogenated and carboxylated graphene sheets dispersed in polar and nonpolar short polymer matrices, in order to gain insight into the effects of the edge group functionalization of graphene sheets on the properties of hybrid graphene-based materials. Poly(ethylene oxide) and polyethylene serve as the polar and nonpolar matrix, respectively. In Fig. 9 the structural properties of the short polymer chains, i.e. their mean square end-to-end distance $\left\langle R_\text{e}^2 \right\rangle$, and the radius of gyration, $\left\langle R_\text{g}^2 \right\rangle$, for the chains, whose center of mass is placed within a given layer. The five layers employed are set up in accordance with the positions of the minima in the density profiles (cf. Fig. 9). The results for all five parallel layers and both polymer matrices are plotted in Fig. 9. The data are normalized by the bulk values. The error bars correspond to the standard deviation and were obtained through typical block averaging techniques. In both matrices (PEO and PE), chains in the first layer appear to be slightly swollen with $\left\langle R_\text{e}^2 \right\rangle$ and $\left\langle R_\text{g}^2 \right\rangle$ higher than the bulk values. In the case of PE, the difference is larger, which can be caused by its tendency to lie flat on the surface [169]. Small deviations from the bulk values are observed also for the second and the third layer, while beyond the fourth layer the vales of $\left\langle R_\text{e}^2 \right\rangle$ and $\left\langle R_\text{g}^2 \right\rangle$ are consistent with those in the bulk within error bars.

**Fig. 10:**
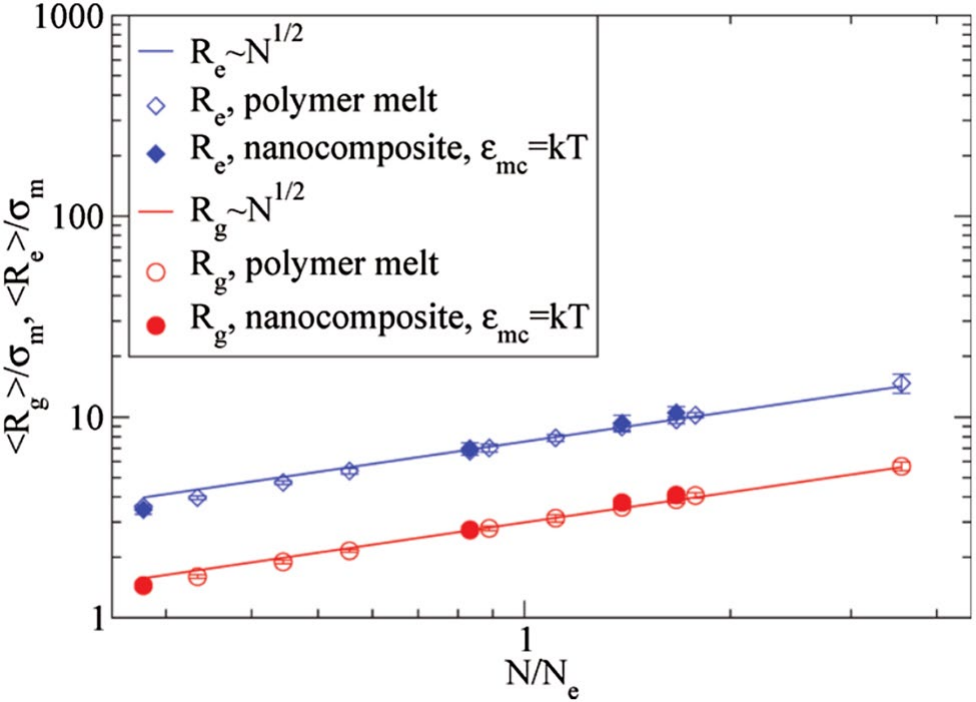
End-to-end distance and radius of gyration of polymer chains of different molecular weight of a polymer/SWCNT ($r_\text{SWCNT} = 0.66$) nanocomposite system from molecular dynamics simulations: (i) end-to-end distance of a polymer melt (*blue open diamonds*), (ii) fitting of the scaling law $R_\text{e} \sim N^{1/2}$ on the simulation data (*blue line*), (iii) end-to-end distance of polymer chains in contact with the SWCNT, interacting with $k_\text{B}T$ energy with the SWCNT (*blue filled diamonds*), (iv) radius of gyration of a polymer melt (*red open circles*), (v) fitting of the scaling law $R_\text{g} \sim N^{1/2}$ on the simulation data (*red line*), and (vi) radius of gyration of polymer chains in contact with the SWCNT, interacting with $k_\text{B}T$ energy with the SWCNT (*red filled circles*). (Color figure online) (Reprinted with permission from Ref. [170]. Copyright (2011) American Chemical Society.)

Karatrantos et al. [170] investigated the static properties of monodisperse polymer/single wall carbon nanotube (SWCNT) nanocomposites by molecular dynamics simulations of a polymer coarse grained model [171, 172]. The SWCNT studied had a large aspect ratio and radius smaller than the polymer radius of gyration (e.g. in a well dispersed PS/SWCNT nanocomposite the radius of the nanotube is of the order of the Kuhn length of PS). Polymer chains are composed of bead-spring chains of Lennard-Jones monomers $\text{m}$, of diameter $\sigma _\text{m} = 1$ and mass $m_\text{m}$ = 1. Three different SWCNTs ((12,0), (17,0), (22,0) of radius $r_\text{SWCNT} = 0.46 \sigma _\text{m}$, $0.66 \sigma _\text{m}$, $0.85\sigma _\text{m}$, respectively) are considered and span the simulation cell with their atoms held fixed in a centered position in the simulation cell along the *z*-axis. In Fig. 10, root mean squared average $\left\langle R_\text{e} \right\rangle$ and $\left\langle R_\text{g} \right\rangle$ of the polymer chains that remained in contact with the SWCNT (so polymers in the polymer/SWCNT simulations that do not always contact the SWCNT were omitted from those values) are shown. As can be clearly seen, the dimensions of polymer chains in contact with the SCWCNT almost overlap with those in the polymer melt for all the polymer molecular weight when interacting with the SWCNT with energy in the $k_\text{B}T$ range.

### End Grafted Polymers onto Nanoparticles

Controlling the spatial dispersion of nanoparticles is critical to the ultimate goal of producing polymer nanocomposites with desired macroscale properties. Experimental studies [14, 73, 150] have shown that, often, nanoparticles tend to aggregate into clusters, with the property improvements connected to their nanoscale dimension being lost. One commonly used technique for controllably dispersing them is end grafting polymer chains to the nanoparticle surface, so that nanoparticles become “brush coated” [14]. When the coverage is high enough, the nanoparticles are sterically stabilized, which results in good spatial dispersion [173, 174]. Moreover, spherical nanoparticles uniformly grafted with macromolecules robustly self-assemble into a variety of anisotropic superstructures when they are dispersed in the corresponding homopolymer matrix [14].

A simpler system that is useful for understanding polymer brushes grafted on spherical nanoparticles immersed in melts is that of a brush grafted to a planar surface in contact with a melt of chemically identical chains. Important molecular parameters for this system are the Kuhn segment length of the chains, *b*, the lengths (in Kuhn segments) of the grafted, $N_\text{g}$ and free, $N_\text{f}$, chains, and the surface grafting density (chains per unit area), $\sigma$. The case of planar polymer brushes exposed to low molecular weight solvent was studied theoretically by de Gennes [175] and Alexander [176]. These authors used a scaling approach in which a constant density was assumed throughout the brush: all the brush chains were assumed to be equally stretched to a distance from the substrate equal to the thickness of the brush. Aubouy et al. [177] extracted the phase diagram of a planar brush exposed to a high molecular weight chemically identical matrix. Their scaling analysis is based on the assumption of a steplike concentration profile and on imposing the condition that all chain ends lie at the same distance from the planar surface. Five regions with different scaling laws for the height, *h*, of the brush were identified. For low enough grafting densities, $\sigma < N_\text {g}^{-1}a^{-2}$ (with *a* being the monomer size) and short free chains, $N_\text {f} < N_\text {g}^{1/2}$, the brush behaves as a swollen mushroom, with $h \sim N_\text{g}^{3/5}$. By keeping the grafting density below $N_\text{g}^{-1}a^{-2}$ and increasing $N_\text{f}$, so that $N_\text{f}>N_\text{g}^{1/2}$, the brush becomes ideal with $h \sim N_\text{g}^{1/2}$. For intermediate grafting densities, $N_\text{g}^{-1}< \sigma < N_\text{g}^{-1/2}$, high molecular weight free chains, $N_\text{f}>N_\text{g}^{1/2}$, can penetrate the brush, thus ideally wetting it and leading to $h \sim N_\text{g}^{1/2}$. Increasing the grafting density while keeping $N_\text{f}<N_\text{g}^{1/2}$ forces the chains to stretch, thus leading to a brush height scaling as $h \sim N_\text{g}$.

Wijmans and Zhulina [178] employed similar ideas in order to understand the configurations of polymer brushes grafted to spherical nanoparticles dispersed in a polymer melt. Here the radius of the nanoparticle, $R_\text{n}$, enters as an additional parameter. Long polymers grafted to a surface at fixed grafting density, $\sigma$, are strongly perturbed from their ideal random-walk conformation [179]. Planar geometry scaling (infinite radius of curvature) is inadequate to explain the case when the particle size is reduced to a level comparable with the size of the brushes. The SCF theory has been applied to convex (cylindrical and spherical) surfaces by Ball et al. [179]. For the cylindrical case, under melt conditions, it was found that the free ends of grafted chains are excluded from a zone near the grafting surface. The thickness of this dead zone varies between zero for a flat surface to a finite fraction of the brush height, *h*, in the limit of strong curvature, when $R_\text {n}/h$ is of order unity, with $R_\text {n}$ being the radius of curvature of the surface.

Borukhov and Leibler [180] presented a phase diagram for brushes grafted to spherical particles, in which the five regions of the work of Aubouy et al. can still be located, but they also provide the scaling of the exclusion zone, where matrix chains are not present. Such exclusion zones have been observed in a special limiting case of grafted polymers, namely, star shaped polymers. Daoud and Cotton [181] showed that, in the poor-solvent limit, the free ends of the chains are pushed outward, because of high densities near the center of the star. The Daoud-Cotton model assumes that all chain ends are a uniform distance away, while the Wijmans-Zhulina model [178] has a well-defined exclusion zone. For $\Theta$ solvents, in the limit of large curvature (small particle radius, $R_\text {n}$), the segment density profile, $\phi (r)$, decreases with the radius as [178] $\phi (r) \propto \sigma ^{1/2} \left( R_\text {n}/r\right)$. It must be noted that $\phi$ is not linear in $\sigma$ because the brush height depends on $\sigma$. In the limit of small curvature (large $R_\text {n}$), a distribution of chain ends must be accounted for [182], leading the segment density profile to a parabolic form: [178] $\phi (r') = \frac{3\sigma N_\text {g} b^3}{h_0}\left( \frac{h}{h_0}\right) ^2\left( 1-\left( \frac{r'}{h}\right) ^2\right)$ where *b* is the statistical segment length, $r'= r-R_n$ is the radial distance from the particle surface, $h_0$ is the effective brush height for a flat surface and *h* is the brush thickness. For large nanoparticles, the above form asymptotically recovers the planar result ($h \rightarrow h_0$). In the case of intermediate particle radii, a combination of large and small curvature behaviors is anticipated: [178] the segment density profile exhibits large curvature behavior near the surface of the particle, followed by a small curvature behavior away from it. Finally, following Daoud and Cotton, the brush height is expected to scale as $h \propto \sigma ^{1/4} N_\text {g}^{1/2}$. Recently, Chen et al. [183] revisited the scaling laws for spherical polymer brushes and identified significant assumptions overlooked by Daoud and Cotton.

#### Experimental Findings

Hasegawa et al. [173] used rheological measurements and SCF calculations to show that particles are dispersed optimally when chains from the melt interpenetrate, or wet, a grafted polymer brush (“complete wetting”). This occurs at a critical grafting density, which coincides with the formation of a stretched polymer brush on the particle surface [175, 176]. Grafting just below this critical density produces aggregates of particles due to attractive van der Waals forces between them. The results of these authors suggest that mushrooms of nonoverlapping grafted polymer chains have no ability to stabilize the particles against aggregation (“allophobic dewetting”). Grafting just above this critical density also results in suboptimal dispersions, the aggregation of the particles now being induced by an attraction between the grafted brushes. For high curvature (small radius) nanoparticles, the polymer brush chains can explore more space, resulting in less entropic loss to penetrate the brush, reducing the tendency for autophobic dewetting.

Until recently, the experimental verification of theoretical and simulation predictions was mostly limited to global information concerning the brush, such as its average height, but not its profile [184]. Recently Chevigny et al. [185] used a combination of X-ray and Small Angle Neutron Scattering (SANS) techniques to measure the conformation of chains in polystyrene polymer brushes grafted to silica nanoparticles with an average radius of $13\,\text {nm}$ dispersed in polystyrene matrix. They found that, if the molecular weight of the melt chains becomes large enough, the polymer brushes are compressed by a factor of two in thickness compared to their stretched conformation in solution. Also, polymer brushes exposed to a high molecular weight matrix are slightly compressed in comparison to brushes exposed to a low molecular weight matrix environment. This observation implies a wet to dry conformational transition. The low molar mass free chains can penetrate into the grafted brush and swell it (“wet” brush). Conversely, when grafted and free chain molar masses are comparable, free entities are expelled from the corona (“dry” brush). Later, they examined the dispersion of these grafted particles in melts of different molar masses, $M_\text {f}$ [186]. They showed that for $M_\text {g}/M_\text {f}<0.24$, the nanoparticles formed a series of compact aggregates, whereas for $M_\text {g}/M_\text {f}>0.24$, the nanoparticles were dispersed within the polymer host.

#### Insight Obtained from Simulations

**Fig. 11:**
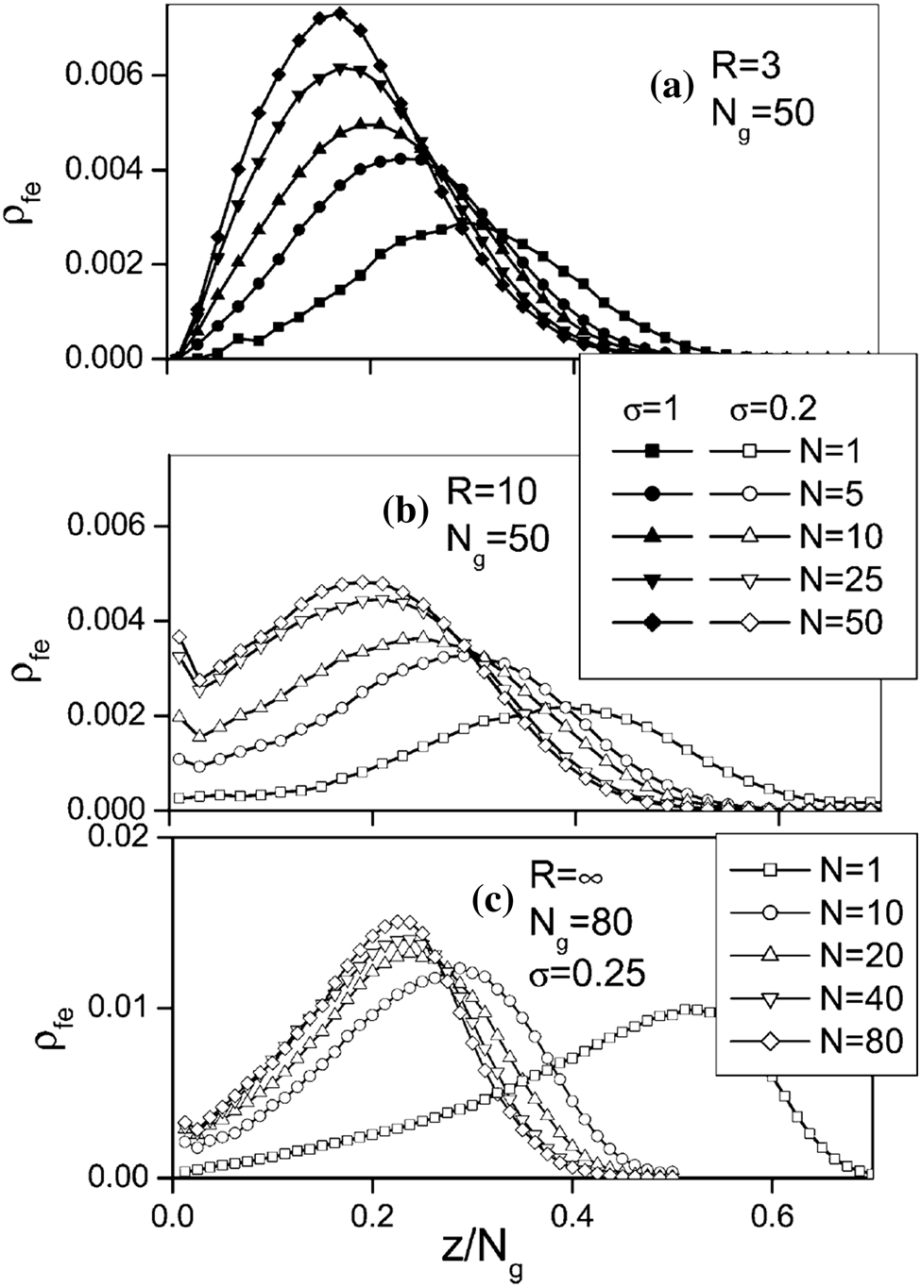
**a** Concentration of the free ends of grafted chains, $\rho _\text{fe}\left( z\right)$ as a function of the distance *z* from the sphere surface for chains with $N_\text{g} = 50$ grafted to the a sphere of radius $R = 3$ for various lengths of free chains, *N*, and grafting densities. **b** Same as in (**a**) except $R = 10$.** c** Same as in (**a**) except $N_\text{g}=80$, $R \rightarrow \infty$. (Reprinted with permission from Ref. [187]. Copyright (2004) American Chemical Society.)

Klos and Pakula [187] simulated linear flexible polymers of $N_\text{g}$ repeat units, end-grafted at density $\sigma$ onto a spherical surface of radius *R* (“hairy nanoparticle”), including the case of flat impenetrable wall ($R \rightarrow \infty$) using their cooperative motion algorithm [188, 189]. The simulations were carried out for a wide range of parameters characterizing the hairy surfaces ($N_\text{g}$, $\sigma$, and *R*) and concerned in detail the influence of length of matrix chains on the anchored ones. That was achieved by gradually varying the polymerization degree *N* of the matrix chains between the two extremes of a dense melt of identical chains ($N = N_\text{g}$) and a simple solvent consisting of single beads ($N =1$). Their analysis of free grafted chain-ends concentrations, $\rho _\text{fe}\left( z\right)$, is shown in Fig. 11 (a, b) for $R=$3, 10 and for $\sigma =$1, 0.2, respectively. The length unit used in their work was *c* / 2 with *c* being the lattice constant of the employed lattice Monte Carlo simulations. The curves indicate how the medium in which the hairy sphere is immersed influences the profiles of free ends of the grafted chains. For both sizes of the spheres, the observed tendency is such that the longer the matrix chains become, the closer to the surface the free ends concentrate. In particular, this is also the case for chains grafted to a flat surface, as presented in Fig. 11(c). Furthermore, for $N = N_\text{g}, R=10,$ and $R\rightarrow \infty$, the concentration of the free ends is finite even at the surface, which means that a small fraction of the ends concentrate in that region, creating grafted chain loops, in agreement with earlier Molecular Dynamics simulations of brushes on flat surfaces by Grest [190].

Spatial integration of the radial mass density profiles around the nanoparticle allows for estimating the height of the grafted polymer brush, which is usually defined as the second moment of the segment density distribution, $\rho (r)$, as [178, 191]:

62 $$\begin{aligned} \left\langle h^2 \right\rangle ^{\frac{1}{2}} = \left[ \frac{\int _{R_n}^{\infty } 4 \pi r^2 dr (r-R_\text{n})^2 \rho (r)}{\int _{R_n}^{\infty } 4 \pi r^2 dr \rho (r)} \right] ^{\frac{1}{2}} \end{aligned}$$

with respect to the height $h \equiv r - R_\text {n}$. However, comparison with experimental brush heights requires a measurement of where the major part of the grafted material is found. To this effect, the brush height can also be arbitrarily defined as the radius marking the location of a spherical Gibbs dividing surface, in which 99% of the grafted material is included. The theory of spherical polymer brushes was pioneered by Daoud and Cotton [181]. In analogy to the scaling model developed by Alexander and de Gennes for planar interfaces, Daoud and Cotton developed a model for spherical surfaces through geometric considerations based on starlike polymers. The spherical brush is divided into three regions, an inner meltlike core region, an intermediate concentrated region (dense brush), and an outer semidilute region (swollen brush). Daoud and Cotton predicted for star shaped polymers in the matrix a change in the scaling behavior as the blobs of the chains become non-ideal. The density profile is directly related to the average brush height, *h*, or the extension of the corona chains. Neglecting the contribution of the core to the radius of the star, they found that $h \propto N_\text {g}^{1/2} \sigma ^{1/4} b$. Although the former relation exhibits “ideal” scaling with respect to the chain length dependence, the presence of the factor $\sigma _\text{r}^{1/4}$ shows that the radius is in fact larger than it would be for a single linear chain. Thus, although we are in a regime where the chain seems to be ideal, the structure is actually stretched.

**Fig. 12:**
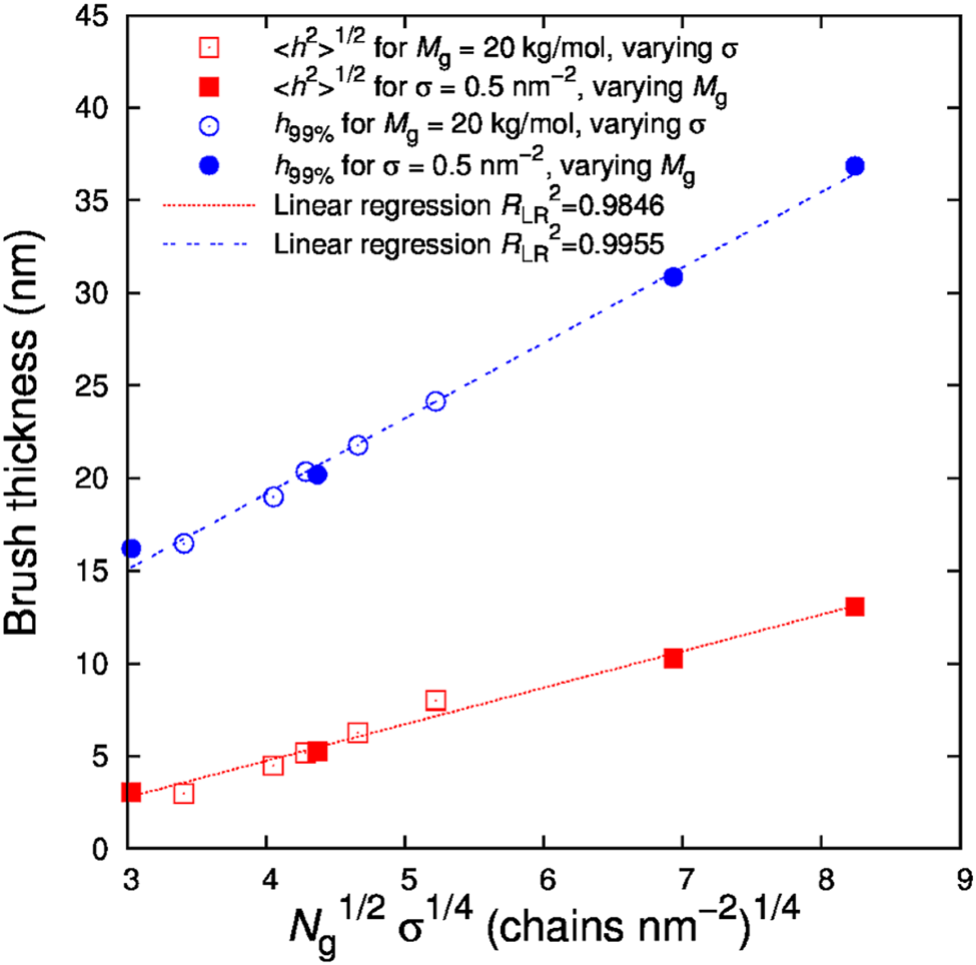
The calculated brush thickness (either $\left\langle h^2 \right\rangle ^{\frac{1}{2}}$ or $h_{99\%}$) is plotted versus the degree of polymerization of grafted chains, $N_\text {g}$, times the grafting density, $\sigma ^{1/4}$. Points correspond to systems containing an 8-nm-radius silica particle grafted with PS chains and dispersed in PS matrix. Linear behavior is predicted by the model proposed by Daoud and Cotton for star shaped polymers [181]. (Reprinted with permission from Ref. [192]. Copyright (2011) American Chemical Society.)

Vogiatzis and Theodorou [192] investigated the structural features of polystyrene brushes grafted on spherical silica nanoparticles immersed in polystyrene by means of a Monte Carlo methodology based on polymer mean field theory. The nanoparticle radii (either 8 or 13 nm) were held constant, while the grafting density and the lengths of grafted and matrix chains were varied systematically in a series of simulations. The primary objective of that work was to simulate realistic nanocomposite systems of specific chemistry at experimentally accessible length scales and study the structure and scaling of the grafted brush. In Fig. 12 the average thickness is plotted versus $N_\text {g}^{1/2}\sigma ^{1/4}$. $N_\text {g}$ is measured in Kuhn segments per chain and $\sigma$ in chains per $\text {nm}^{2}$. The scaling prediction of Daoud and Cotton seems to be fullfilled for both the rms height $\left\langle h^2 \right\rangle ^{\frac{1}{2}}$ and the height containing 99% of the brush material, $h_{99\%}$. The dashed lines are linear fits, confirming the good agreement of the simulation data with the theoretical scaling behavior. The agreement seems to be better for the $h_{99\%}$ data points. This was expected, since the average brush thickness, as defined in Eq. (62), is more sensitive to the discretization of the model and to the post processing of the data, than the straightforward definition of the shell in which the 99% of the brush material can be found. Moreover the results for the $h_{99\%}$ estimate and the scattering pattern of the whole grafted corona were in favorable agreement with SANS measurements.

**Fig. 13:**
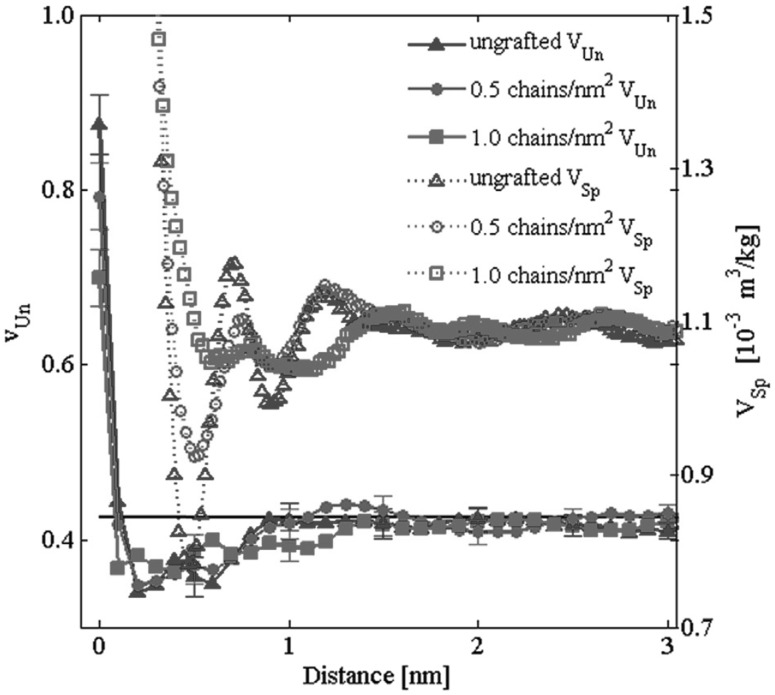
Distribution of unoccupied volume fraction, $v_\text{Un}$, (*left* *y*-axis) and the specific volume, $v_\text{Sp}$, (*right* *y*-axis) in the vicinity of a nanoparticle with a diameter of 3 nm at a termperature of 590 K. The*horizontal line* corresponds to the PS bulk value of $v_\text{Un}$. The grafting density is varying from 0.0 (ungrafted) to 0.5 and 1.0 grafted chains / nm$^2$. (Reprinted from [193] with permission from Elsevier.)

Voyiatzis et al. [193] studied the confinement induced effects on the accessible volume and the cavity size distribution in polystyrene-silica nanocomposites by atomistic Molecular Dynamics simulations. The composite systems contained a single $\alpha$-quartz silica nanoparticle, either bare or grafted with atactic PS chains, which was embedded into an unentangled atactic PS matrix [158]. Both free and grafted chains consisted of 20 monomers. The considered nanoparticle diameters were 3.0, 4.0 and 5.0 nm and three different grafting densities were studied: 0.0, 0.5 and 1.0 chains / nm$^2$. Those authors investigated the cavity distribution size by employing four spherical probe particles. Apart from the limiting case of a dot-like probe particle (zero radius), the considered probe particles had radii, $r_\text{p}$, of 0.128, 0.209 and 0.250 nm, corresponding to hard-sphere representations of helium, methane and ethane. The “unoccupied” volume was defined as the volume accessible to a probe particle of $r_\text{p} = 0$.

The influence of the grafting density on the spatial distribution of the unoccupied volume fraction, $v_\text{Un}$, and the specific volume, $v_\text{Sp}$, for a nanoparticle with a diameter of 3 nm is presented in Fig. 13. The black horizontal line corresponds to the bulk value of $v_\text{Un}$. The greatest reductions of $v_\text{Un}$ relative to the bulk value occur very close to the surface, at distances smaller than 1 nm. The variation in the $v_\text{Un}$ distribution of a grafted and an ungrafted nanoparticle is different. The separation from the particle for which $v_\text{Un}$ is below the bulk value for the grafted 1.0 chains/nm$^2$ system exceeds by approximately 50% the distance for the ungrafted system. The $v_\text{Un}$ profile for a grafting density of 0.5 chains / nm$^2$ lies in between the two extremes. Unlike $v_\text{Un}$, the $v_\text{Sp}$ spatial distributions exhibit a strong dependence on the grafting density. An increase of the grafting density leads to increased $v_\text{Un}$ values close to the particle’s surface. This behavior was attributed to the (i) the chemistry of the employed linker molecule and (ii) the expulsion of the free chains from adsorbing on the nanoparticle surface. Contrary to intuitive expectations, variations of the accessible volume were not directly related to changes of the specific volume.

## Dynamics

A complete understanding of PNC dynamics requires confronting the difficult many-body problem associated with non-dilute particle concentrations and coupled nanoparticle and polymer motions over many time- and length- scales [194–196]. Simulations are a valuable option, but are computationally very intensive, resulting in only a limited parameter range being tractable to study, typically involving rather small particles and weakly entangled polymers. The transport properties of nanoparticle-polymer mixtures have been the focus of much recent attention [197–207]. Central problems in the area are the diffusion of nanoparticles and polymers through the nanocomposite melts, as well as the local polymer dynamics in the proximity of the filler particles. For example, the incorporation of nanoparticles can strongly modify the viscosity of polymer melts [208], and the center-of-mass mobility of polymer chains can be strongly retarded depending on nanoparticle size and concentration [209, 210].

### Nanoparticle Diffusion in Polymers

#### Experimental Findings

There is good understanding of the motion of very large or very small colloidal particles of radius $R_\text{n}$ in a polymer melt. The nanoparticle diffusion coefficient, *D*, in the large particle limit follows the classic continuum Stokes-Einstein relation [211]. For a large and massive solute molecule of radius $R_\text{n}$ in a solvent consisting of much smaller and lighter molecules, the diffusion coefficient, *D*, of the solute is given by [212]:

63 $$\begin{aligned} D = \frac{k_\text{B}T}{f \pi \eta R_\text{n}} \end{aligned}$$

where $k_\text{B}$ is Boltzmann’s constant, *T* is the absolute temperature, $\eta$ is the solvent viscosity and *f* takes the values of 4 or 6 for slip or stick boundary conditions at the solute surface, respectively [203]. The corresponding behavior of small nanoparticles, comparable to the size of a monomer, is also described by the Stokes-Einstein relationship but with a length-scale dependent viscosity that is smaller than the macroscopic bulk value [213]. The relevant apparent viscosity is controlled by the relaxation of subsections of chains with an end-to-end distance comparable to the nanoparticle size, as has been verified by Molecular Dynamics simulations [214]. Understanding nanoparticle diffusion in the polymer matrix is of fundamental importance.

Despite the rather good understanding of the diffusion of the particles in the two limits (very large and very small), the dynamical behavior of nanoparticles of size comparable to the entanglement mesh size of the polymer is contentious [205, 215–217]. Brochard-Wyart and de Gennes [213] argued that the particle diffusion constant follows normal Stokes-Einstein behavior essentially when its size becomes larger than the entanglement mesh size. Such a sharp size-dependent crossover to Stokes-Einstein scaling has been observed by Szymanski et al. [218]. On the contrary, Cai et al. [201] speculated that the motion of these intermediate sized nanoparticles should be faster than Stokes-Einstein behavior, since diffusion can be facilitated by hoplike motions trough the polymer’s entanglement mesh. The latter is also supported by a microscopic force-level theory, wherein chain relaxation and local entanglement mesh fluctuations, i.e. “constraint release”, dominate over hopping [219].

Somoza et al. [220] studied experimentally the anthracene rotation in poly(dimethylosiloxane) and poly(isobutylene) by gradually increasing the chain length. These authors reported that the diffusivity of the particles exhibited a sharp transition with the increase of the polymer radius of gyration, $R_\text{g}$, becoming dependent on the “nanoviscosity” (rotation time of dissolved anthracene was used as a measure of the viscosity on a nanometer-sized object) rather than the macroscopic viscosity for small $R_\text{n}/R_\text{g}$ ratios (with $R_\text{n}$ being the particle size). Narayaman et al. [221] used X-ray photon correlation spectroscopy in conjunction with resonance-enhanced grazing-incidence small-angle X-ray scattering to probe the particle dynamics in thin films, and also found that the particle dynamics differ from the Stokes-Einstein Brownian motion, the difference being caused by the viscoelastic effects and interparticle interactions. Meanwhile, Tuteja et al. [198] reported that the nanoparticles diffuse two orders of magnitude faster in a polymer liquid than the prediction of the Stokes-Einstein relation, an observation possibly attributable to the nanoparticles being smaller than the entanglement mesh. Later, Grabowski et al. [199] also observed strong enhancement (250 times) of the diffusion of gold nanoparticles in poly(butyl methacrylate), under conditions where the nanoparticle dimensions were smaller than the entanglement mesh length of the polymer.

Cai et al. [201] have carried out an extensive study of nanoparticle diffusion by employing scaling theory to predict the motion of a probe nanoparticle of size $R_\text{n}$ experiencing thermal motion in polymer solutions and melts. Particles with size smaller than the solution correlation length, $\xi$, undergo ordinary diffusion with a diffusion coefficient similar to that in pure solvent. The motion of particles of intermediate size ($\xi< d < \alpha _\text{pp}$), where $\alpha _\text{pp}$ is the tube diameter for entangled polymer liquids, is subdiffusive at short time scales, since their motion is affected by subsections of polymer chains. At long time scales the motion of these particles is diffusive, and their diffusion coefficient is determined by the effective viscosity of a polymer liquid with chains of size comparable to the particle diameter $R_\text{n}$. The motion of particles larger than the tube diameter $\alpha _\text{pp}$ at time scales shorter than the relaxation time $\tau _\text{e}$ of an entanglement strand is similar to the motion of particles of intermediate size. At longer time scales ($t> \tau _\text{e}$) large particles ($d> \alpha _\text{pp}$) are trapped by the entanglement mesh, and to move further they have to wait for the surrounding polymer chains to relax at the reptation time scale $\tau _\text{rep}$. At longer times $t> \tau _\text{rep}$, the motion of such large particles $(d> \alpha _\text{pp})$ is diffusive with diffusion coefficient determined by the bulk viscosity of the entangled polymer liquids. Finally, for nanoparticles with diameters larger than the entanglement mesh size it appears that the competition of full chain relaxation versus the nanoparticle hopping through entanglement gates controls nanoparticle diffusion [222].

#### Insight Obtained from Simulations

**Fig. 14:**
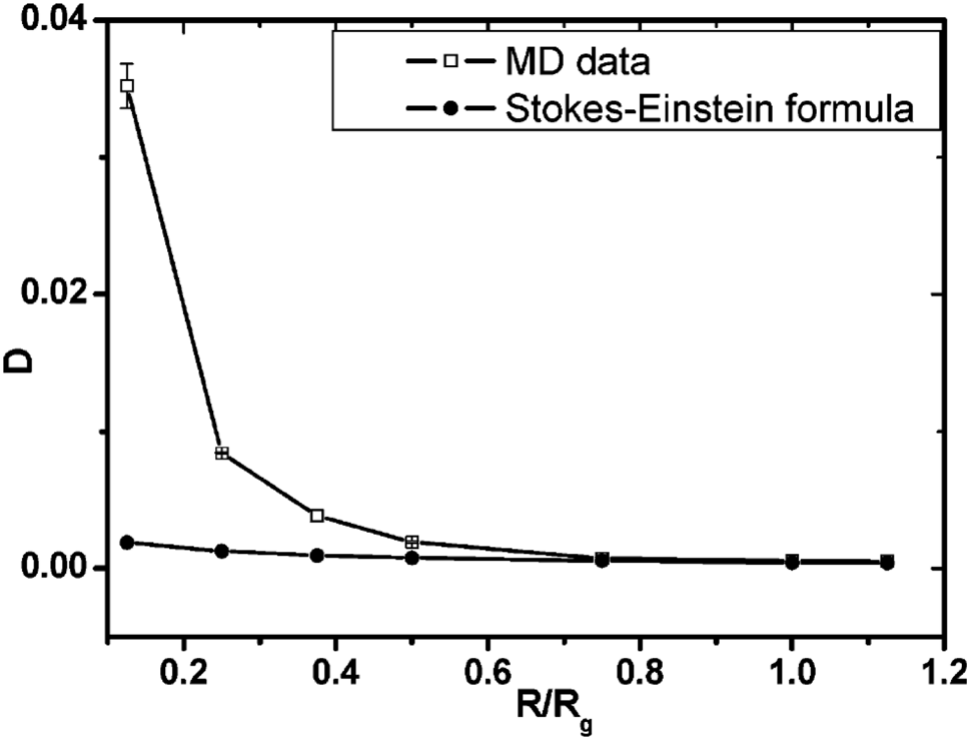
The diffusion coefficient, *D*, of a spherical particle as a function of the ratio $R_\text{n}/R_\text{g}$. The particle mass is proportional to its volume. Open squares represent Molecular Dynamics data, while full* dots* represent the Stokes–Einstein relation predictions with slip boundary condition. (Reprinted with permission from Ref. [214]. Copyright (2008) American Chemical Society.)

Liu et al. [214] employed Molecular Dynamics simulations of the Kremer-Grest model [149] in order to investigate the diffusion process of spherical nanoparticles in polymer melts. Their results indicated that the radius of gyration of the polymer chains was the key factor determining the validity of the Stokes-Einstein relation in describing the particle diffusion at infinite dilution. In Fig. 14 the diffusion coefficient estimated by the MD simulations is presented alongside the predictions of the Stokes-Einstein formula. It was found that, with the increase of the size ratio of $R_\text{n}/R_\text{g}$, the Stokes-Einstein diffusion coefficient gradually approximates the MD data under the slip (dotted curve) boundary condition. The use of purely repulsive non-bonded interactions fully justifies the use of the slip ($f=4$), instead of the stick ($f=6$), boundary condition. As the size ratio, $R_\text{n}/R_\text{g}$ increases to 1, the predicted diffusion coefficients agree reasonably well with those extracted from the simulations. However, in the small size ratio, large deviation is observed which qualitatively agrees with the experimental results [198]. It seems like the local viscosity experienced by nanoparticles is much smaller than the macroscopic viscosity, as speculated by Wyart and de Gennes [213] and other researchers [223–226]. Finally, it should be noted that in the experiments chains are strongly entangled, in the reptation regime, while the polymer length used by Liu et al. is smaller than the entanglement length of the polymer chain [227].

**Fig. 15:**
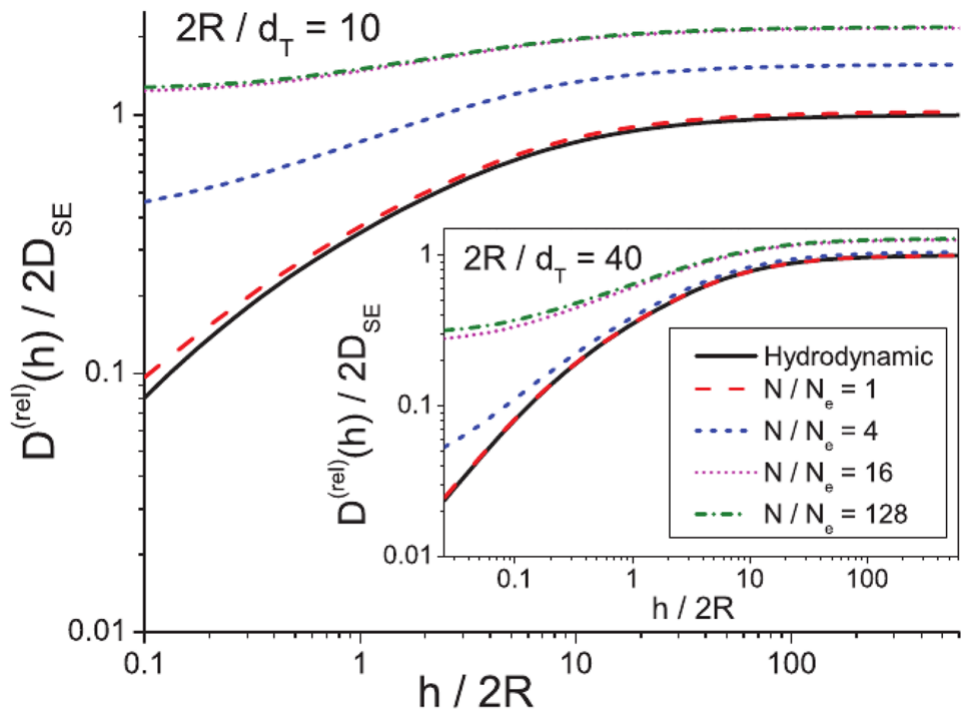
Relative diffusivity normalized by the single-particle Stokes-Einstein self-diffusion coefficient as a function of $h/2 R_\text{n}$ (with *h* being the interparticle surface-to-surface separation distance), based on the structural continuum model of Yamamoto and Schweizer [219, 228]. Calculations are presented for $2R_\text{n}/d_\text{T} = 10$ (with $d_\text{T}$ representing the tube diameter) and $N/N_\text{e} =1$ (*dashed line*), 4 (*short-dashed line*), 16 (*short-dotted line*), and 128 (*dashed-dotted line*), with $N_\text{e}$ being the number of chain segments per entangled strand. The hydrodynamic result (*solid curve*) is also included as a reference. In the* inset* to the figure, the same results as the main frame are presented, for larger particle size, $2R_\text{n}/d_\text{T} = 40$. (Reprinted from [228], with the permission of AIP Publishing.)

Yamamoto and Schweizer [219, 228] have formulated and applied a microscopic statistical-mechanical theory, based on the Polymer Reference Interaction Site Model (PRISM) integral equation theory [229], for the non-hydrodynamic relative diffusion coefficient of a pair of spherical nanoparticles in entangled polymer melts. Their work was based on a combination of Brownian motion, mode-coupling, and polymer physics ideas. They focused on the mesoscopic regime, where particles are larger than the entanglement spacing. The overall magnitude of the relative diffusivity was controlled by the ratio of the particle to tube diameter and the number of entanglements per chain. Figure 15 presents model calculations of the total relative diffusivity, $D^\text{(rel)}\left( h\right)$ as a function of $h/2R_\text{n}$ (with *h* being the interparticle surface-to-surface separation distance) for two reduced particle diameters. The ordinate of the figure is normalized by the single particle Stokes-Einstein result, $D_\text{SE}$, while the abscissa extends up to the point where the theory is argued to be reasonable. That theory is based on the mode-coupling idea that the relevant slow dynamical variable is the bilinear coupling of the nanoparticle and the collective polymer density fluctuations. The original approach [219] was not self-consistent since it assumed that the constraining forces on a particle relax entirely due to the length-scale dependent motions of the polymer melt (constraint release regime), which is an accurate simplification when particles are larger than $d_\text{T}$.

Figure 15 exhibits several interesting trends. First, the relative diffusivity approaches the asymptotic value $D^\text{(self)}/D_\text{SE}$ at $h/2R_\text{n}>> 1$, verifying the “isolated” limit (two particles at infinite dilution). Note that it does not necessarily approach unity if a Stokes-Einstein violation is present at the single-particle level. The overall deviation of $D^\text{(rel)}$ from the hydrodynamic result is enhanced as $2R_\text{n}/d_\text{T}$ decreases or $N/N_\text{e}$ increases, in analogy with a single-particle Stokes-Einstein violation effect. The underlying physical mechanism can be understood as small nanoparticles acquiring high mobility due to a weaker coupling to the slow relaxation of entangled melts compared to the continuum theory. As a consequence, the overestimate of friction by a hydrodynamic approach grows as particle size decreases and/or chain length increases. The second general feature in Fig. 15 concerns the role of the number of entanglements, $N/N_\text{e}$. Deviations cannot be discerned from the hydrodynamic behavior for weakly entangled cases ($N/N_\text{e} \sim 1$) for either particle size. One may physically rationalize this result by recalling that the Rouse- like collective relaxation is diffusive above the segmental length-scale. Finally, the most important feature of Fig. 15 is the predicted non-trivial mobility enhancement compared to the hydrodynamic result over a wide range of $h/2R_\text{n}$. Hydrodynamics predicts zero relative diffusivity as $h \rightarrow 0$, whereas the results of Yamamoto and Schweizer remain non-zero down to small separations.

**Fig. 16:**
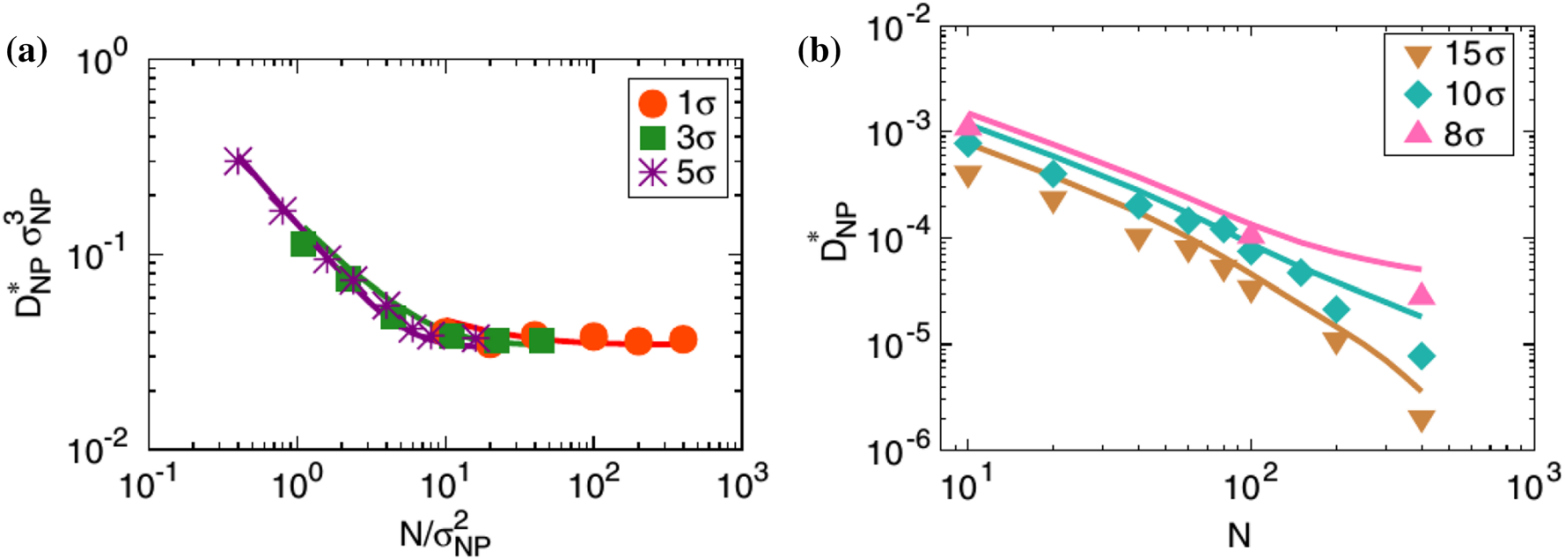
Terminal diffusion coefficient of nanoparticles of different size, $D^*_\text{NP}$, in melts of different *N* plotted in scaled form. The combination of the Stokes-Einstein equation with the Rouse model viscosity for a melt of chain length $N_\text{NP}$, $\eta = \eta _1 N_\text{NP}$ (where $\eta _1$ is the viscosity of a monomer fluid at the same density) yields that the quantity $D^*_\text{NP} \sigma _\text{NP}^3$ should be a constant, independent of chain length [201, 214].** a** Nanoparticles that are smaller than the entanglement mesh size, $\sigma _\text{NP} < d_\text{T}$.** b** Nanoparticles that are larger than the entanglement mesh size, $\sigma _\text{NP} \ge d_\text{T}$. In all cases, a slip boundary condition was assumed. Predictions of the theory of Yamamoto and Schweizer [228] are presented in* solid lines*. Moreover, if we estimate the Rouse viscosity based on the actual chain length *N*, we get $D^*_\text{NP} \sigma _\text{NP}^3 \sim (N/\sigma _\text{NP}^2)^{-1}$, which corresponds to the decaying curves on the* left-hand side* of (**a**). (Reprinted figure with permission from [230]. Copyright 2014 by the American Physical Society)

Kalathi et al. [230] have employed large-scale molecular dynamics simulations in order to examine the role of entanglements on nanoparticle dynamics in the crossover regime, where the diameter of the particles, $\sigma _\text{NP}$, is larger than $2 - 10 d_\text{T}$ with $d_\text{T}$ being the entanglement tube diameter. The transport behavior of nanoparticles in the crossover size limit appears to be complicated by hopping effects, length-scale dependent entanglement forces and dynamics, and the interactions of polymers and nanoparticles. These authors simulated weakly interacting mixtures of nanoparticles and bead-spring polymer melts. For the polymer melts considered in that work, the entanglement chain length is approximately 45, *N*_e_~45, and $d_\text{T}$ in units of monomer diameter, $\sigma$, is around 7 (the nanoparticle diameters were $\sigma _\text{NP} = 1-15$, in units of polymer $\sigma )$. The diffusion coefficients of nanoparticles smaller than $d_\text{T}{\sim}7 - 10$ (i.e. $\sigma _\text{NP} = 1$, 3, and 5, respectively, Fig. 16(a)) in long chain melts show that the relevant viscosity corresponds to a section of the chain with $N_\text{NP}$ monomers that satisfies $\sigma ^2_\text{NP} = N_\text{NP}\sigma ^2$. For shorter chains the data can be described by the Stokes-Einstein relation with the macroscopic viscosity of the chain fluid, i.e.,

64 $$\begin{aligned} D_\text{NP}^* = \frac{k_\text{B}T}{f \pi \eta \sigma _\text{NP}} = \frac{k_\text{B}T}{f \pi \eta _1 N \sigma _\text{NP}} \end{aligned}$$

where $\eta = \eta _1 N$ (where $\eta _1$ is the viscosity of a monomer fluid at the same density) and $f{\sim}4$ (asymptote in Fig. 16(a)). The results of Kalathi et al. [230] for smaller nanoparticles are in good agreement with the predictions of the theory of Yamamoto and Schweizer [228]. Figure 16(b) presents the results for nanoparticles with sizes larger than the entanglement mesh length, $d_\text{T}{\sim}7 -10$. The diffusion of these particles in the longer chain melts does not follow the “universal” plateau seen for small nanoparticles. These results suggest that the chain-scale dynamics does not control nanoparticle diffusion (no Stokes-Einstein scaling). However, the fact that the diffusivity at high *N* of these intermediate-sized particles does not reach the same plateau as the small nanoparticles (Fig. 16) suggests that another effect, probably entanglements, plays an important role. Despite the fact that no conclusive evidence of hopping-controlled transport was found, the spontaneous fluctuations of the entanglement mesh (constraint release) in the moderately long chain melts, may be the most important mode of nanoparticle transport through the bulk of the material, in agreement with theoretical predictions [228].

### Polymer Diffusion and Dynamics

Early theoretical studies [231–233] have shown that polymer diffusion through heterogeneous media is slowed down due to entropy losses associated with impenetrable obstacles. A natural choice of a parameter for quantifying the ability of a nanocomposite to slow down polymer diffusion is the spacing between the surfaces of neighboring nanoparticles [234]. For monodisperse nanoparticles, this spacing can be defined as the interparticle distance, $d_\text{inter}$, given by [235]:

65 $$\begin{aligned} d_\text{inter} = d \left[ \left( \frac{\phi _\text{max}}{\phi }\right) ^{\frac{1}{3}} -1\right] \end{aligned}$$

where *d* and $\phi$ are the nanoparticle diameter and nanoparticle volume fraction, respectively.The maximum packing density of the nanoparticles, $\phi _\text{max}$, depends on the packing type, such as simple cubic ($\phi _\text{max} = 0.524$), face-centered cubic ($\phi _\text{max} = 0.740$), body-centered cubic ($\phi _\text{max} = 0.680$), and random dense packing ($\phi _\text{max} = 0.637$). Independently of $\phi _\text{max}$, $d_\text{inter}$ decreases as nanoparticle size decreases at fixed $\phi$, suggesting that smaller nanoparticles slow down polymer diffusion more effectively than larger ones [231, 232].

#### Experimental Findings

**Fig. 17:**
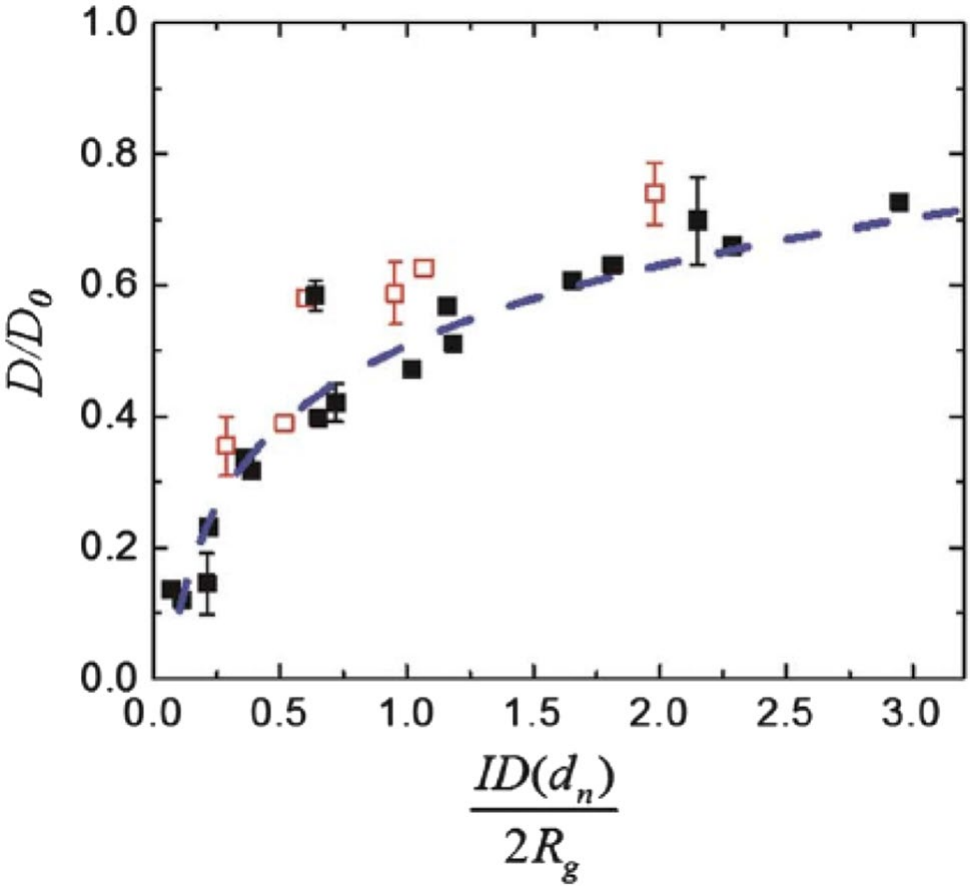
Reduced diffusion coefficient of a dPS polymer tracer $(D/D_0)$ in a silica-polystyrene nanocomposite, plotted against the confinement parameter, namely the interparticle distance, $ID=d_\text{inter},$ relative to the tracer size, $R_\text{g}$.* Open* and* closed squares* represent experimental data for nanoparticles with number average diameters of 12.8 and 28.8 nm. Employing the interparticle distance estimated from the average number nanoparticle diameter for monodisperse particles, the scaling of $D/D_0$ seems reasonable, although the values for the smaller particles are higher than those for the larger particles. (Reprinted from [210]—Published by The Royal Society of Chemistry.)

Gam et al. [210] have measured the tracer diffusion of deuterated polystyrene (dPS) in a polystyrene nanocomposite containing silica nanoparticles, with number average diameters, $d_\text{n}$, of 28.8 and 12.8 nm, using elastic recoil detection. The corresponding volume fractions of the large and small nanoparticles, $\phi$, ranged from 0 to 0.5, and 0 to 0.1, respectively. At the same volume fraction of nanoparticles, the tracer diffusion of dPS is reduced as nanoparticle size decreases because the interparticle distance between nanoparticles, $d_\text{inter}$, decreases. The reduced diffusion coefficient, defined as the tracer diffusion coefficient in the nanocomposite relative to pure PS ($D/D_0$) is plotted against the confinement parameter divided by the tracer size in Fig. 17. All measurements nearly collapse onto a master curve [234], although $D/D_0$ is slightly higher for the smaller particles. For $d_\text{inter} = ID < 2R_\text{g}$, $D/D_0$ decreases rapidly as $ID/2R_\text{g}$ decreases. For $ID> 2 R_\text{g}$, $D/D_0$ remains less than 1 indicating that entropy loss reduces diffusion even when nanoparticles are far apart relative to the tracer size. The dashed line is an empirical fit, because a theory relating *D* to the fundamental system parameters is lacking.

Schneider et al. [236] studied experimentally the relaxation of entangled poly(ethylene-alt-propylene) (PEP) chains (tube diameter ~5 nm) filled with silica nanoparticles (average diameter ~17 nm). The silica volume fraction was varied between 0.0 and 0.6 (as that was estimated from the measured weight fraction of silica in the nanocomposite). Neutron spin echo spectroscopy (NSE) was empoloyed in order to explore chain dynamics in these nanocomposites, characterized by non-attractive interactions. The resulting collective dynamic scattering function data were analyzed by employing the idea of a tube-like confinement for chain relaxation below the reptation time. The following conclusions were drawn from their study: (i) the monomeric relaxation rates were not unaffected by the addition of nanoparticles, even at high particle loadings; (ii) chain conformations remain Gaussian for all loadings considered; and (iii) the tube diameter determined from analysis of neutron spin echo data decreases monotonically upon adding nanoparticles. Two contributions to overall chain dynamics were speculated. On the one hand, the number of topological chain-chain entanglements decreases with increased nanoparticle loading, i.e., the chains disentangle from each other since a part of the system volume is occupied by the NPs. On the other hand, the chain acceleration caused by the reduction of entanglements is (more than) compensated by the geometric constraints that nanoparticles present to chain dynamics. Since that second factor dominates at large loadings, the neutron scattering experiments suggested an increase in chain relaxation time, while at the same time a reduction of chain-chain entanglements and an increase of particle-chain entanglements take place.

#### Insight Obtained from Simulations

**Fig. 18:**
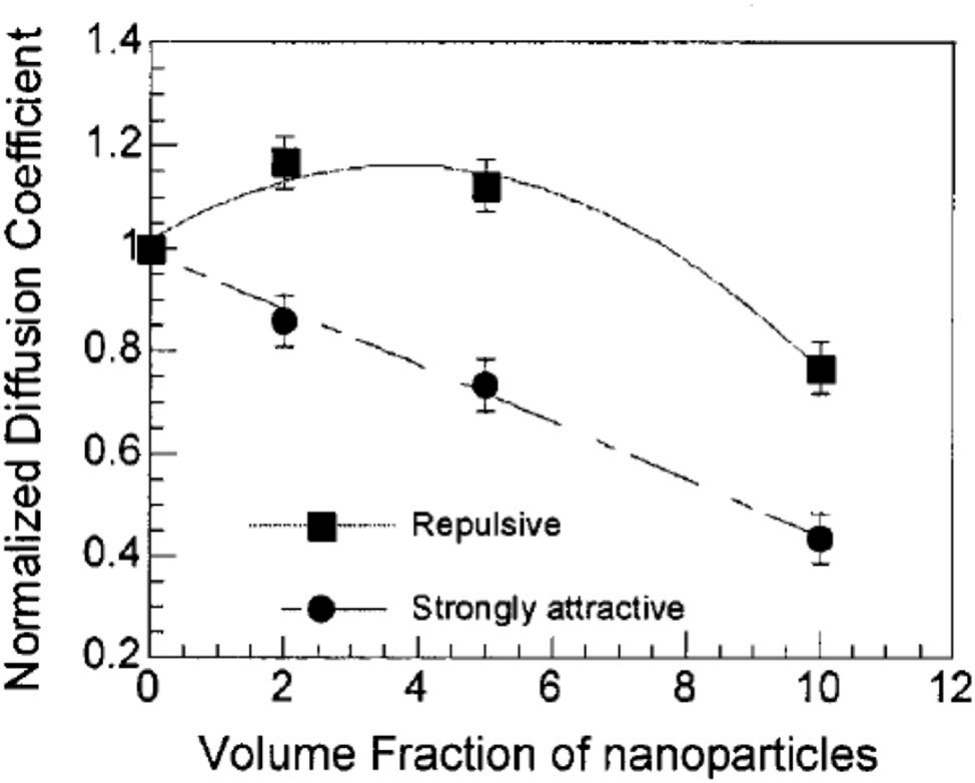
The normalized (by its bulk value) overall diffusion coefficient of bead-spring polymer chains as a function of % volume fraction of nanoparticles for repulsive and strongly attractive systems. (Reprinted from [237], with the permission of AIP Publishing.)

Desai et al. [237] investigated the chain dynamics of Kremer-Grest polymer melts, composed of chains with a relatively high degree of polymerization ($N=80$) filled with solid nanoparticles using molecular dynamics simulations. These authors found that chain diffusivity is enhanced relative to its bulk value when polymer–particle interactions are repulsive and is reduced when polymer–particle interactions are strongly attractive (Fig. 18). In both cases chain diffusivity assumes its bulk value when the chain center of mass is about one radius of gyration, $R_\text{g}$, away from the particle surface. As shown in Fig. 18 for the particle volume fraction of 10%, the average chain diffusion coefficient is reduced by a factor of 2 in the presence of strongly attractive particles. The case of repulsive particles appears to be even more interesting, where the diffusion coefficient initially increases with increasing particle concentration, but then reaches a maximum before decreasing with further increase of the particle concentration. While the initial particle concentration dependence of the diffusion coefficient reflects the polymer–particle interactions, higher concentrations always lead to a reduction of the diffusion coefficient, which may be attributed to geometrical reasons (i.e. the presence of tortuous paths in systems with high particle loadings).

**Fig. 19:**
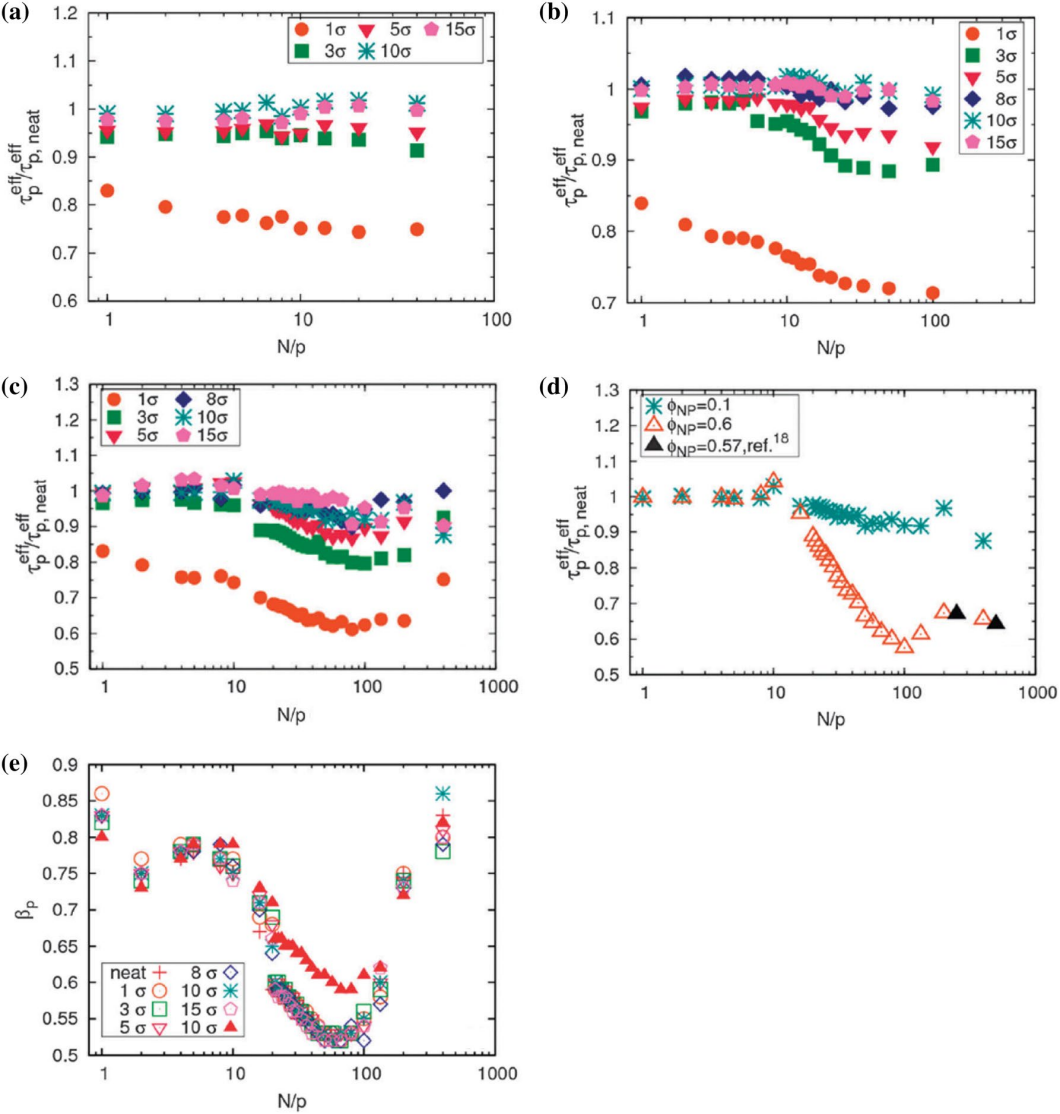
Normalized effective relaxation times of the *p*-th Rouse mode for chains in nanocomposites for different nanoparticle sizes at $\phi = 0.1$:** a** $N=40$;** b** $N=100$;** c** $N=400$.** d** Effect of nanoparticle loading for $N= 400$, $\sigma _\text{NP}=10 \sigma$ (*closed triangles *correspond to $\sigma _\text{NP} = 10 \sigma$ in $N=500$ at similar nanoparticle loading as in ref. [238]). **e** Corresponding plot for the stretching exponent $\beta$. (Reprinted from [239]—Published by The Royal Society of Chemistry.)

Kalathi et al. [239] employed large-scale molecular dynamics simulations in order to study the internal relaxations of chains in nanoparticle/polymer composites. They examined the Rouse modes of the chains, which resemble the observables of the self-intermediate scattering function, typically determined in an (incoherent) inelastic neutron scattering experiment. The Rouse modes, $p =0,1,2,...,N-1$, of a chain of length *N* are defined as [240]:

66 $$\begin{aligned} \mathbf {X}_{p} = \sqrt{\frac{2}{N}} \sum _{i=1}^{N} \mathbf {r}_i \cos {\left[ \frac{p \pi }{N}\left( i - \frac{1}{2}\right) \right] } \;\;. \end{aligned}$$

The time autocorrelation of the Rouse modes is predicted to decay exponentially and independently for each node *p* for an ideal chain, with relaxation time, $\tau _p$. The $p=0$ mode describes the motion of the chain center-of-mass, while the modes with $p \ge 1$ describe internal relaxations with a mode number *p* corresponding to a sub-chain of $\left( N-1\right) /p$ segments. The comparison of the relaxation times of the different modes for chains in the PNCs for three different degrees of polymerization, *N*, filled with nanoparticles of different sizes for $\phi = 0.1$ to neat melt is presented in Fig. 19(a)–(c). Their results (Fig. 19) showed that, for weakly interacting mixtures of nanoparticles and polymers, the effective monomeric relaxation rates are faster than in neat melt when the nanoparticles are smaller that the entanglement mesh size. In this case, the nanoparticles serve to reduce both the monomeric friction and the entanglements in the polymer melt, as in the case of polymer-solvent mixtures. On the contrary, for nanoparticles larger than half the entanglement mesh size, the effective monomer relaxation remains unaffected for low nanoparticle concentrations. Even in this case, strong reduction of chain entanglements was observed. These authors concluded that the role of nanoparticles is to always reduce the number of entanglements. By assuming that the relaxation time for a chain follows the crossover bridging Rouse to reptation dynamics, the large *p* modes directly yield information on the monomer friction and in the limit of $p=1$, the plateau of $\tau _\text{p}^\text{eff}/\tau _\text{p,neat}^\text{eff}$ is directly proportional to the ratio of $\tau _0/N_\text{e}$ in the PNC compared to that in the pure melt. For small nanoparticles, which act as a diluent, there was an additional speedup, which was attributed to a reduction in entanglements, quantified by $N_\text{e, melt}/N_\text{e, PNC}{\sim}0.9$ for long chains, which can also be extracted by the stretching exponents (Fig. 19(e)).

### Local Polymer Dynamics

#### Insight Obtained from Simulations

**Fig. 20:**
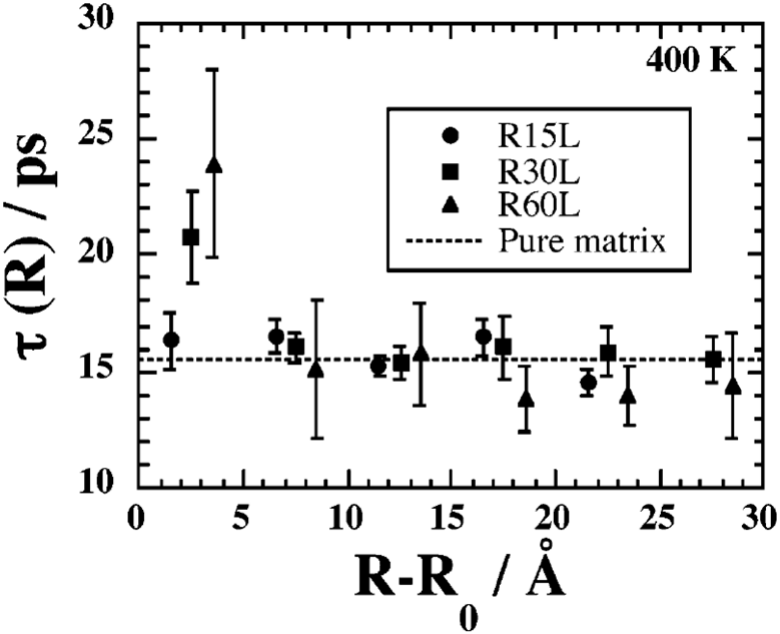
The radial dependence of the relaxation time of the torsional autocorrelation function of polyethylene around a silica nanoparticle. All values are averages taken in 5 Åshells around the nanoparticle center of mass. The points have been offset slightly for the three systems along the *x* axis for clarity. The* dotted line* simply indicates the value obtained for the 30-chain pure polymer system at the same temperature ($400 \; \text{K}$). (Reprinted with permission from Ref. [167]. Copyright (2008) American Chemical Society.)

Brown et al. [167, 241] were among the first to study the local dynamics of a model nanocomposite system. They examined the structure and dynamics of a system containing an inorganic (silica) nanoparticle embedded in a polymer (polyethylene-like) matrix. They thoroughly discussed the variation of structure and dynamics with increasing distance from the polymer–particle interface and as a function of pressure. A clear structuring of the linear polymer chains around the silica nanoparticle was observed, with prominent first and second peaks in the radial density function and concurrent development of preferred chain orientation. Evidence of chain immobilization was less obvious overall, although dynamic properties were more sensitive to changes in the pressure. Long simulations were carried out to determine the variation in the glass transition of the filled polymers as compared to the pure systems. In Fig. 20 the average relaxation times of the torsional autocorrelation function are presented, resolved into concentric shells of 5 Å thickness around the nanoparticle center of mass. This assignment was based on the position of the center of mass of the four atoms involved in the torsion at the time origin of the observation. Although this means that at some later time an angle may belong to a different shell, it avoids the bias that would result from selecting only angles that remain in a particular shell (in any case diffusion of chains is relatively slow so that should not be a problem). Based on Fig. 20, there is some indication that the decreased translational mobility near the interface increases the relaxation time associated with torsional equilibration (establishment of the *trans*-*gauche* equilibrium in the systems containing the nanoparticles with diameters of 3 and 6 nm (R30L and R60L, respectively), but otherwise most of the characteristic times are very close to those obtained for the neat system, in agreement with previous studies [241, 242] of the same authors. It was concluded that, within errors, the interphase thickness was independent of the size of the nanoparticle for the range of particle systems analyzed.

**Fig. 21:**
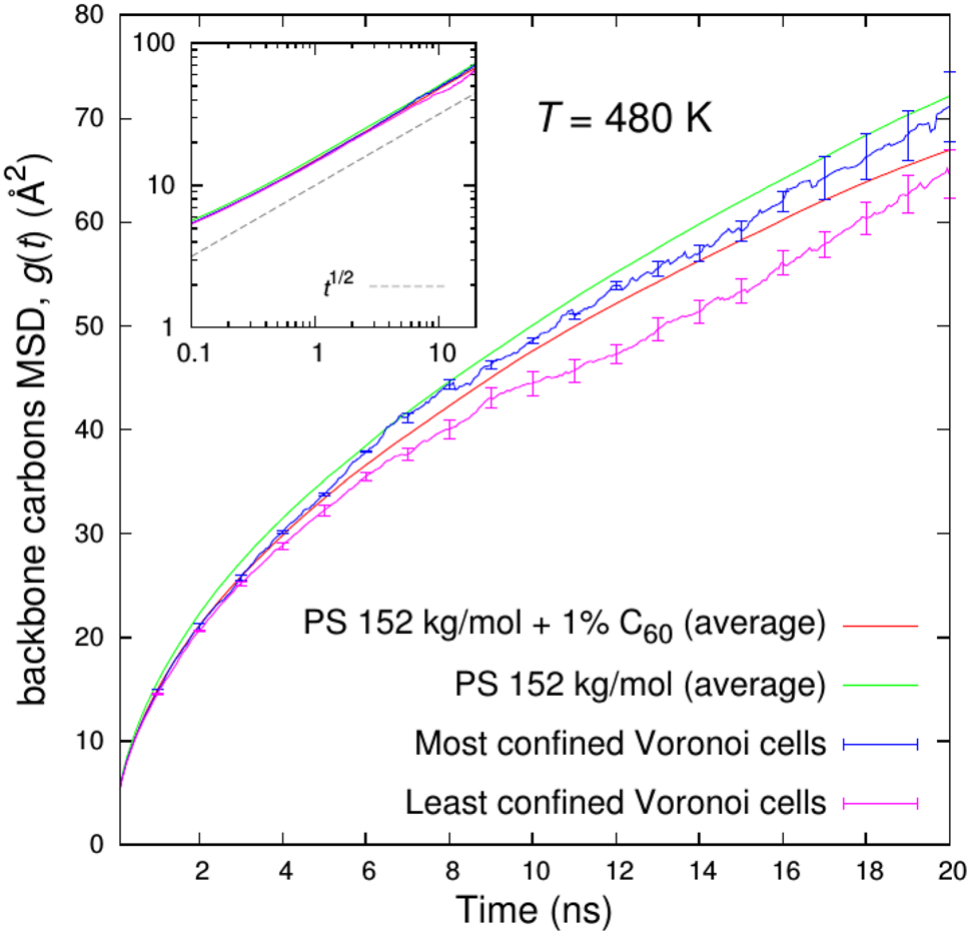
Mean-squared atomic displacements (MSD) of backbone carbon atoms as a function of time for filled and unfilled polystyrene systems at T = 480 K. In the case of fullerene nanocomposites, an analysis of the dependence of backbone MSD on confinement is also presented for most and least confined Voronoi cells (indicative error bars also included). In the* inset* to the figure, the same data are presented in logarithmic axes. (Reprinted with permission from Ref. [156]. Copyright (2014) American Chemical Society.)

Vogiatzis and Theodorou [156] produced atomistic configurations of fullerene-filled polystyrene melts by reverse mapping well-equilibrated coarse-grained melt configurations, sampled by connectivity altering Monte Carlo, to the atomistic level via a rigorous quasi Metropolis reconstruction. The main goal of their work, i.e., the study of PS-C$_{60}$ dynamics at the segmental and local levels, has been accomplished by analyzing long MD trajectories of their well-equilibrated reverse-mapped structures. Their simulation results generally indicate that the addition of C$_{60}$ to PS leads to slower segmental dynamics (as estimated by characteristic times extracted from the decay of orientational time-autocorrelation functions of suitably chosen vectors), suggesting an increase of the bulk glass transition temperature, $T_\text{g}$, by about 1 K, upon the addition of C$_{60}$ at a concentration of 1% by weight (in favorable agreement with differential scanning calorimetry measurements [243]). They then employed a space discretization in order to study the effect of C$_{60}$ on segmental dynamics at a local level. Each fullerene served as the center of a Voronoi cell, whose volume was determined by the neighboring fullerenes. Backbone carbons lying in every cell were tracked throughout the atomistic Molecular Dynamics trajectory and their mean-square displacement (MSD) was measured for the time they resided in the same cell. Overall mean-square displacement of backbone atoms was found to be smaller in the presence of fullerenes, than in bulk PS. However, atoms moving in smaller (more confined) Voronoi cells exhibited faster motion than the atoms moving inside larger Voronoi cells. Figure 21 presents the MSD of backbone carbon atoms as a function of time at a temperature of 480 K for both the filled and unfilled systems. As can be seen, nanocomposite systems exhibit lower mobility when compared to their neat counterparts. The MSD of backbone carbons is depressed upon the addition of fullerenes, in good agreement with the neutron scattering observations of Kropka et al. [243]. In the inset to Fig. 21, a logarithmic plot of the MSD is presented. The scaling of $t^{1/2}$ is expected for the very short time behavior studied, as Likhtman and McLeish [244] have estimated that the time marking the onset of the effect of topological constraints on segmental motion, $\tau _\text{e}$, is $3.36\times 10^{4} \;\text{s}$ for polystyrene.

Moreover, Vogiatzis and Theodorou [156] estimated the local mean-square displacement (MSD) of backbone carbon atoms of PS, for the timespan an atom spends inside a particular cell of the Voronoi tessellation. In their analysis they used the average MSD from the three most confined and three least confined cells. They observed that the volume of the Voronoi cells did not change significantly as a function of time. Based on that analysis for the nanocomposite system, the degree of depression was found to be a function of the confinement induced by the fullerenes. The diffusion of chains was spatially inhomogeneous, as observed by Desai et al. [237] earlier. Small Voronoi cells lead to higher mobility of the polymer segments within them. Despite the fact that the addition of fullerenes limited the diffusion of polymeric chains, there existed regions in space, where the polymer could recover part of its dynamics due to the high level of confinement. This finding was then correlated with the increased rotational diffusion of fullerenes, as the volume of the Voronoi cells became smaller. These authors envisioned fullerenes as nanoscopic millstones, forcing the polymeric chains to diffuse faster, when close to them, while the geometrical constraints imposed by their presence force the chains to diffuse more slowly.

Pandey et al. [245] have extensively studied the local dynamics and the conformational properties of polyisoprene next to a smooth graphite surface constructed by graphene layers, via a multiscale simulation methodology. These authors first performed fully atomistic molecular dynamics simulations of isoprene oligomers, next to the surface. Subsequently, Monte Carlo simulations of a systematically derived coarse-grained model were employed in order to create several uncorrelated structures for polymer systems. A reverse mapping strategy was developed in order to reintroduce atomistic detail into the coarse-grained configurations. Finally, multiple extensive fully atomistic simulations with large systems of long macromolecules were conducted to examine local dynamics in proximity to graphite. Their findings supported the presence of increased dynamic heterogeneity emerging from both intermolecular interactions with the flat surface and intramolecular cooperativity. For each system, Pandey et al. extracted bond orientation autocorrelation functions and sorted them in intervals of $0.03 \; \text{nm}$ based on the position of the midpoint of the c–CH bond throughout the simulation trajectory. For each interval, the autocorrelation curves were averaged weighting by the population of c–CH vectors found in a specific interval from each run. The mean correlation times increased substantially in the proximity of the surface, with dynamics at the surface almost 20 times slower (independently of the molecular weight of the chains) than in bulk PI.

**Fig. 22:**
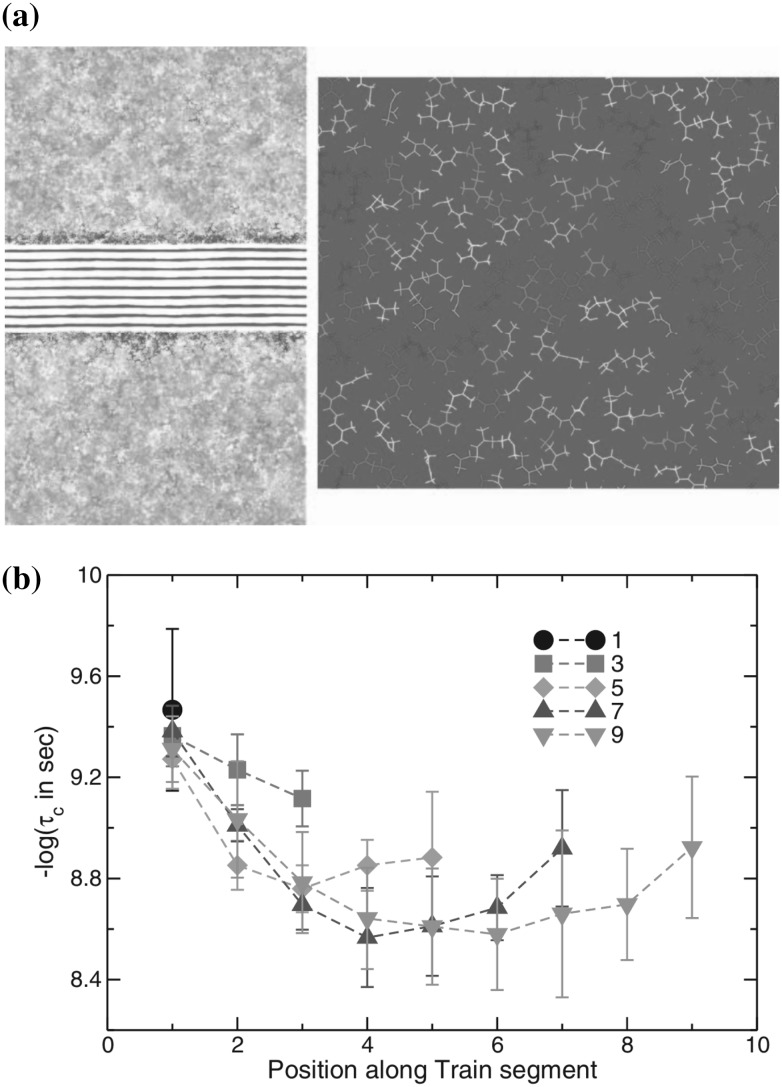
**a** Visual representation of the distribution of $- \log {\tau _\text{c}}$ along the normal to the surface and on the surface of graphene planes ($\tau _\text{c}$ is average per slab relaxation time of the c–CH bonds). Each repeat unit is colored with a scheme where* red* corresponds to the faster segments and blue to the slowest.** b **Average dynamics of repeat units on graphite along a train segment as a function of the length of it. (Color figure online) (Reprinted from [245], with the permission of AIP Publishing.)

Figure 22 presents a qualitative visual inspection of the distribution of times for a specific configuration. Pandey et al. [245] evaluated an autocorrelation function individually for every c-CH vector and colored each segment from blue to red signifying lower relaxation times and higher mobility. As shown, segments in proximity to the surface were found to be slower. They then separated all train segments based on their length and calculated correlation times for each position along the length of the train segment (Fig 22(b)), which was an extremely challenging task. Despite a significant statistical error, several features are evident in Fig.22(b). Specifically, when a chain makes a single contact, dynamics is only decelerated to a small extent. The second important finding was that, as train segments grow in length along the surface, the dynamics of the repeat units becomes progressively slower, with the findings implying that, similar to chain-ends in bulk dynamics, [246, 247] the ends of train segments contribute to increased dynamic heterogeneities on the surface. However, the former are only significant for short chains, the latter were present for any chain length studied. In addition, short train segments can be more pronounced around surfaces with higher curvature [16, 159]. Finally, a PI specific result was the asymmetry present along a train segment originating from the methyl group, much alike findings on bulk dynamics along the chain backbone [247].

**Fig. 23:**
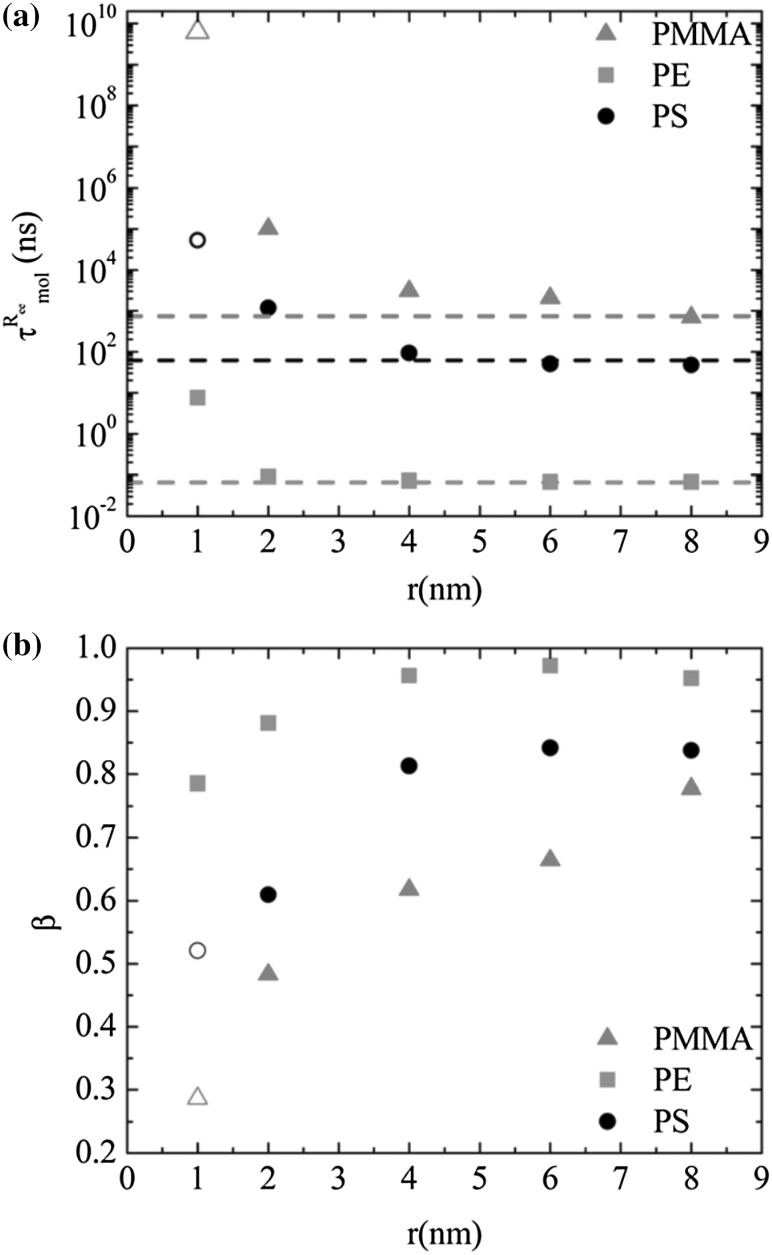
**a** Molecular relaxation time of the end-to-end orientational decorrelation function for PS, PMMA and PE hybrid polymer-graphene systems as a function of the distance from graphene.* Dashed lines* represent the values of the molecular relaxation times of the corresponding bulk systems.** b **The stretching exponent, $\beta$, as extracted from the fit with KWW functions for the three systems. (Reprinted from [248]—Published by The Royal Society of Chemistry.)

Rissanou and Harmandaris [248] presented a detailed analysis of the dynamics of three different polymer-graphene systems, through atomistic Molecular Dynamics simulations. In more detail they studied (a) PS-graphene, (b) PMMA-graphene and (c) PE-graphene interfacial systems, as well as the corresponding bulk polymer systems. For PS and PMMA polymer chains were 10-mers while PE chains were 20-mer, in order to ensure that the backbone consisted of almost the same number of CH$_{2}$, and/or CH groups for all systems (i.e. approximately 20 in all cases). A characteristic quantity of the molecular level is the end-to-end vector $\mathbf {R}_\text{ee}\left( t\right)$, whose autocorrelation function provides information for the orientational dynamics at the entire chain level. Rissanou and Harmandaris performed an analysis of end-to-end autocorrelation function at different adsorption layers and fit the corresponding curves for all chains to the Kohlrausch-Williams-Watts (KWW) function [249–251]. At the entire chain level, the integral below the KWW curves defines the molecular chain end-to-end relaxation time, $\tau _\text{mol}^{\mathbf {R}_\text{ee}}$. The molecular relaxation times together with the stretching exponent, $\beta,$ of the KWW fits are presented in Fig. 23 (a) and (b) as functions of the distance from the surface. Data in Fig. 23(a) reveal the dramatic increase of $\tau _\text{mol}^{\mathbf {R}_\text{ee}}$ close to the graphene layer, compared to the corresponding bulk values, shown with dashed lines. Furthermore, a slight difference in the distance at which the $\tau _\text{mol}^{\mathbf {R}_\text{ee}}$ reaches the plateau distance-independent bulk value was observed: for PE it is about ${\sim}2 \;\text{nm}$, whereas for PMMA and PS is about $3 -4 \; \text{nm}$. The extreme difference in relaxation times between PE and the other two systems is obvious. The $\beta$ exponent of the KWW relation for PE and PS reaches a constant value in the bulk region, while the same does not apply for PMMA. These values show that in the bulk region PMMA has the wider distribution of relaxation times, PS follows and PE has the narrowest one.

## Phase Behavior

Polymer-nanoparticle blends exhibit a rich phase behavior which is directly tied to the thermal, mechanical, and optical properties of the composite system, with the achievement of uniform dispersion being a long-standing challenge [1, 14, 15, 33, 150, 252, 253].

Significant progress towards the development of microscopic predictive theories of the equilibrium structure and phase behavior of polymer nanocomposites has been made recently based on liquid state integral equation formulations, density functional calculations and self-consistent mean field approaches. All these methods can complement or surpass the explicit atom methods like Monte Carlo and Molecular Dynamics, which have the potential to quantitatively predict structural correlations, thermodynamics and phase behavior.

Chatterjee and Schweizer were the first to develop an analytical integral equation theory for treating polymer-induced effects on the structure and thermodynamics of dilute suspensions of hard spheres [254]. Results were presented for the potential of mean force, free energy of insertion per particle into a polymer solution, and the second virial coefficient between spheres. Later, Hoopper et al. [255] employed the Polymer Reference Interaction Site Model (PRISM) theory to investigate structure, effective forces, and thermodynamics in dense polymer-particle mixtures in the one and two particle limit [144, 145].

### Bare Nanoparticles

**Fig. 24:**
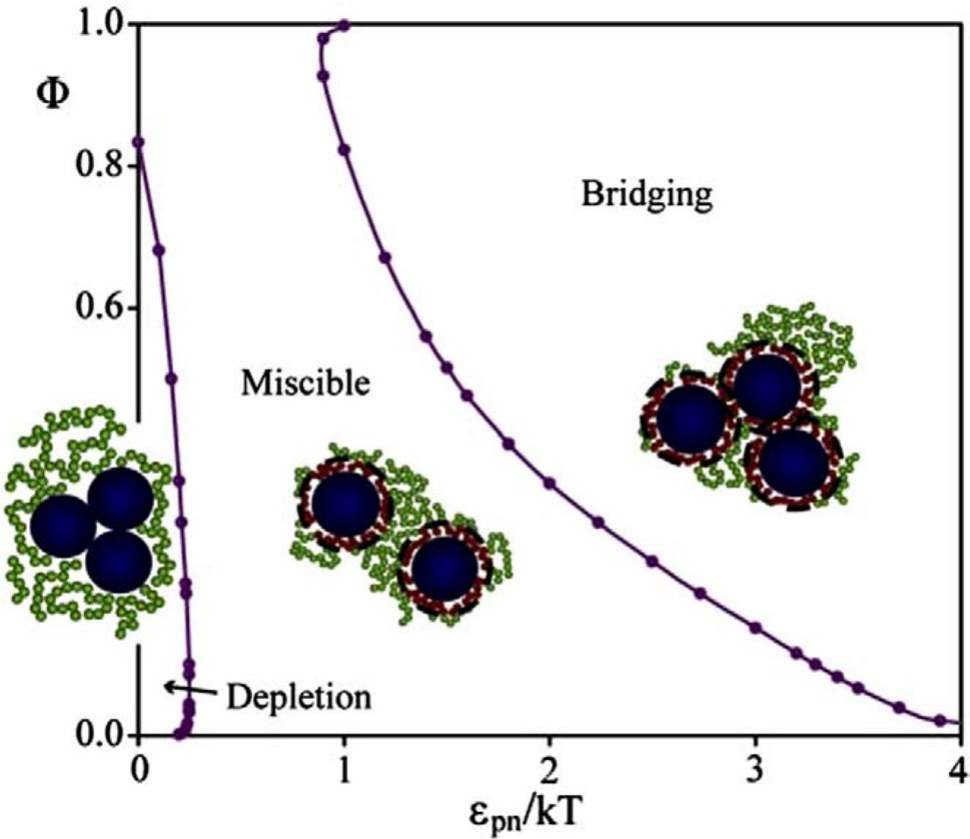
Nanoparticle volume fraction at spinodal phase separation predicted by the Polymer Reference Interaction Site Model (PRISM) theory for hard spheres of $D/d =10$ (with *D* and *d* being the diameters of the nanoparticle and the polymeric beads, respectively) in a freely jointed chain polymer system of length $N=100$, as a function of the strength of exponential interfacial attraction at fixed spatial range. Total mixture packing is 0.4. The depletion and bridging induced phase separated regime bracket a window of miscibility at intermediate interfacial cohesion strength. The type of polymer-mediated nanoparticle organization is schematically indicated. (Reprinted from [256] with permission from Elsevier.)

Hall et al. [256, 257] employed Polymer Reference Interaction Site Model (PRISM) liquid state theory to study phase transitions and structure of dense mixtures of hard nanoparticles and flexible polymer coils. Their calculations were performed over the entire compositional range from the polymer melt to the hard sphere fluid, with the focus being on polymers that adsorb on nanoparticles. Many body correlation effects were fully accounted for in the determination of the spinodal phase separation instabilities. An example phase diagram is presented in Fig. 24. It can be discerned that depletion and bridging phase separation occur at low and high attraction strengths, respectively. Quantitatively, many particle effects are found to always reduce miscibility. Depletion phase separation was similar for different attraction ranges, with a critical point at rather low filler volume fractions. This is in contrast to the bridging induced demixing transition where the critical point is located at very high nanoparticle volume fractions. Moreover, increasing the attraction range increases the thickness of the bound layer and the importance of many body effects, which further decreases miscibility in the high filler volume fraction regime relative to what was predicted by a two particle virial analysis [145]. However, when bridging effects are very strong and phase separation occurs at low volume fractions, decreasing the attraction range can lead to a stronger, shorter range bridging attraction that reduces miscibility. Increasing particle size generally disfavors miscibility on both the depletion and bridging sides of the spinodal phase diagram, though the effect on depletion is more significant.

**Fig. 25:**
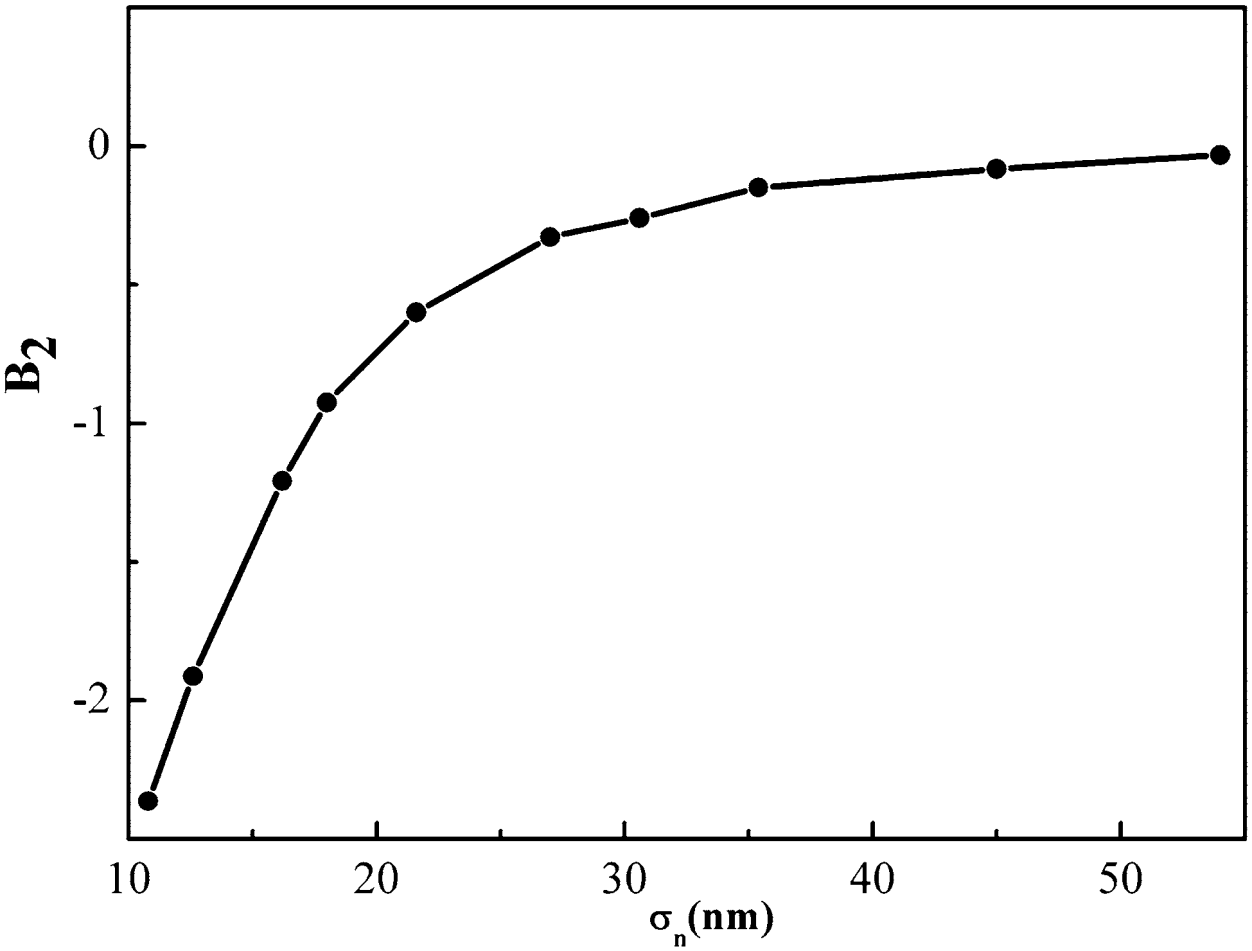
Second virial coefficient as a function of particle size for silica particles dispersed in PS matrix at volume fraction 5 %. (Reprinted with permission from Ref. [258]. Copyright (2015) American Chemical Society.)

Wei et al. [258] have investigated silica nanoparticle dispersions in polystyrene, poly(methyl methacrylate), and poly(ethylene oxide) melts by means of a density functional approach. The polymer chains were regarded as coarse-grained semi-flexible coils whose segment size matched the Kuhn length of the polymer under investigation. The particle-particle and particle–polymer interactions were calculated in the grounds of the Hamaker theory, following Vogiatzis and Theodorou [148, 192]. In order to characterize nanoparticle dispersion, Wei et al. employed the second virial coefficient, $B_2$, defined as:

67 $$\begin{aligned} B_2 = \frac{2}{3}\pi \sigma _\text{n}^3 + 2 \pi \int _{\sigma _\text{n}}^\infty \left[ 1 - \frac{\rho _\text{n}\left( \mathbf {r}\right) }{\rho _\text{n}}\right] r^2 \text{d}r \end{aligned}$$

where the first term accounts for the particle contribution, and the second one is the polymer mediated contribution. The local density of particles is denoted as $\rho _\text{n}\left( \mathbf {r}\right)$, while the average particle density as $\rho _\text{n}$. $\rho _\text{n}\left( \mathbf {r}\right)$ varies as a function of distance between particles, making it a critical link to particle microstructure. The pair correlation function approaches asymptotically $\rho _\text{n}\left( \mathbf {r}\right) /\rho _\text{n} = 0$ when $r < 2 R_\text{n}$ ($R_\text{n}$ being the radius of the particles) as particles cannot interpenetrate and $\rho _\text{n}\left( \mathbf {r}\right) /\rho _\text{n} \simeq 1$ as $r \rightarrow \infty$ as the likelihood of finding a particle becomes proportional to the average particle density. Figure 25 shows the second virial coefficients for different particle sizes at constant particle volume fraction $\phi = 5$%. Positive values of $B_2$ indicate stable particle dispersion (effective particle-particle repulsion), while a negative value signifies unstable dispersion. The results are in agreement with previous theoretical studies: the tendency to dispersion increases as the particle size increases [259]. It can be seen that $B_2$ becomes independent of the particle size after a threshold value, that meaning the effect of particle size on the pair correlation function becomes insignificant. Before that critical value, $B_2$ increases but its increasing amplitude declines as the particle size increases. Density functional theory confirms that large particles are more likely to achieve stable dispersion than small particles.

### Polymer Grafted Nanoparticles

One approach for controlling the particle dispersion in the polymer matrix is to alter the particle surface chemistry through the attachment of polymer chains. The composition, architecture, and distribution of the grafted chains can be carefully designed to tailor interparticle interactions, thereby controlling the dispersion state [33]. In the special case where the chemical composition of the graft and matrix chains are identical, the entropic contributions dominate the thermodynamics [175, 260], and uniform dispersion can be achieved both for the case of spherical nanoparticles [174, 261], and the case of nanorods [262].

#### Experimental Findings

**Fig. 26:**
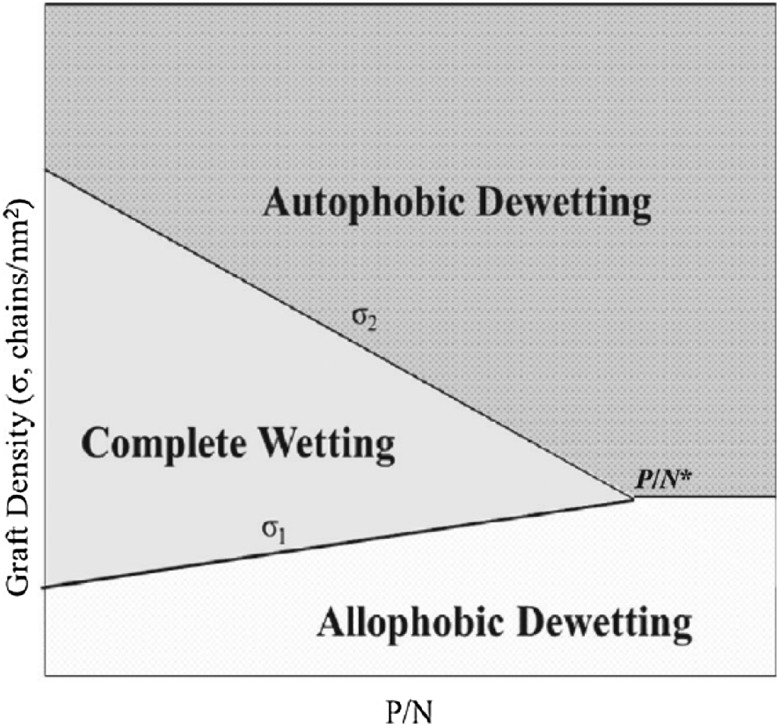
Illustration of the phase diagram for nanoparticle stability as a function of grafting density ($\sigma$) and the ratio of the lengths of the free over the grafted chains (*P*/*N*). Particles at low grafting densities encounter the allophobic dewetting transition at $\sigma _1$. Increasing $\sigma$ leads to complete wetting of the brush by the melt, stabilizing the nanoparticle dispersion. Increasing the graft density further leads to the autophobic dewetting transition at $\sigma _2$ and unstable dispersion. Particle dispersion is unstable at all grafting densities when $P/N> (P/N)^*$. (Reprinted with permission from Ref. [174]. Copyright (2012) American Chemical Society.)

In a number of studies, empirical phase diagrams have been developed for this special case, where the particle miscibility is a function of grafting density, $\sigma$, the ratio between the molecular weight of the grafted chains, *N*, and matrix chains, *P*, i.e. *P* / *N*, as well as the particle radius, $R_\text{n}$ [14, 71, 72, 173, 174, 261, 263–266]. Sunday et al. [174, 267] quantified the stability of polystyrene-grafted silica nanoparticles in PS matrices with ultrasmall angle X-ray scattering (USAXS) and transmission electron microscopy (TEM). They developed the phase diagram presented in Fig. 26 to predict nanoparticle dispersion based on the graft polymer density, $\sigma$, and the graft and free polymer molecular weights, or *N* and *P*, respectively.

The phase diagram of Fig. 26 shows three distinct regions. When $\sigma$ is below the allophobic limit ($\sigma _1$), the surface coverage of grafted chains is low enough that the interactions between the particle and the matrix chains are not screened out sufficiently and the grafted particles behave similarly to block copolymers, aggregating into distinct morphologies [14, 268, 269]. As $\sigma$ increases above $\sigma _1,$ the matrix and grafted chains interpenetrate, resulting in a “wet” brush and repulsive interactions between nanoparticles, thus stabilizing their dispersion. The autophobic dewetting line corresponds to a continuous, second-order transition, resulting from the expulsion of the melt from the brush for densely grafted chains ($\sigma N^{1/2}> 1$) which should lead to nanoparticle aggregation through the attraction between graft layers [270]. Larger values of $R_\text{n}$, $\sigma$, or *P*/*N* result in a larger entropic penalty for intermixing due to crowding of the grafted layer. As the entropic penalty grows, the interpenetration width between the matrix and grafted chains decreases until the matrix chains are completely expelled, resulting in attractive interactions and particle aggregation. This occurs above the autophobic phase transition at $\sigma _2$, a discontinuous, first-order transition at low grafting densities.

Bansal et al. [27] have experimentally observed and modeled the anisotropic self-assembly of small PS-g-silica nanoparticles with cores of $R_\text{n} {\sim}10 - 13 \text{nm}$ in the allophobic dewetting region at lower grafting densities ($\sigma = 0.01 - 0.10 \;\text{chains}/\text{nm}^2$) [14]. Using slightly higher grafting densities ($\sigma {\sim}0.2 - 0.7 \;\text{chains}/\text{nm}^2$), Chevigny et al. [186] used similar-sized PS-g-silica NPs with $N = 5- 50 \;\text{kg/mol}$ in $P = 140 \; \text{kg/mol}$ where particles with the longest grafts ($P/N = 2.8$) dispersed uniformly, whereas those with the shortest grafts ($P/N = 28$) phase separated from the bulk, forming spherical aggregates. The dispersion of silica NPs with higher graft densities has been investigated in which two sets of PS-g-silica NPs, the first with $N = 110$ kg/mol and $\sigma = 0.27 \;\text{chains}/\text{nm}^2$ and the second with $N = 160$ kg/mol and $\sigma = 0.57 \;\text{chains}/\text{nm}^2$ have been shown to disperse at least up to $P/N = 2.3$ [271] and 1.6 [27], respectively.

Another way of tuning the mechanical properties of composite materials is by dispersing hydrophilic nanofillers in highly hydrophobic polymer matrices [272]. Martin et al. [273] have performed simulations and experiments on mixtures containing polymer grafted nanoparticles in a chemically distinct polymer matrix, where the graft and matrix polymers exhibit attractive enthalpic interactions at low temperatures that become progressively repulsive as temperature is increased.

#### Insight Obtained from Simulations

Trombly and Ganesan [264] have calculated the potential of mean force (PMF) between grafted nanoparticles immersed in a chemically identical polymer melt using a numerical implementation of polymer mean-field theory. These authors focused on the interpenetration width between the grafted and free chains and its relationship to the polymer-mediated interparticle interactions. To this end, they quantified the interpenetration width as a function of particle curvature, grafting density, and the relative molecular weights of the grafted and free chains.

**Fig. 27:**
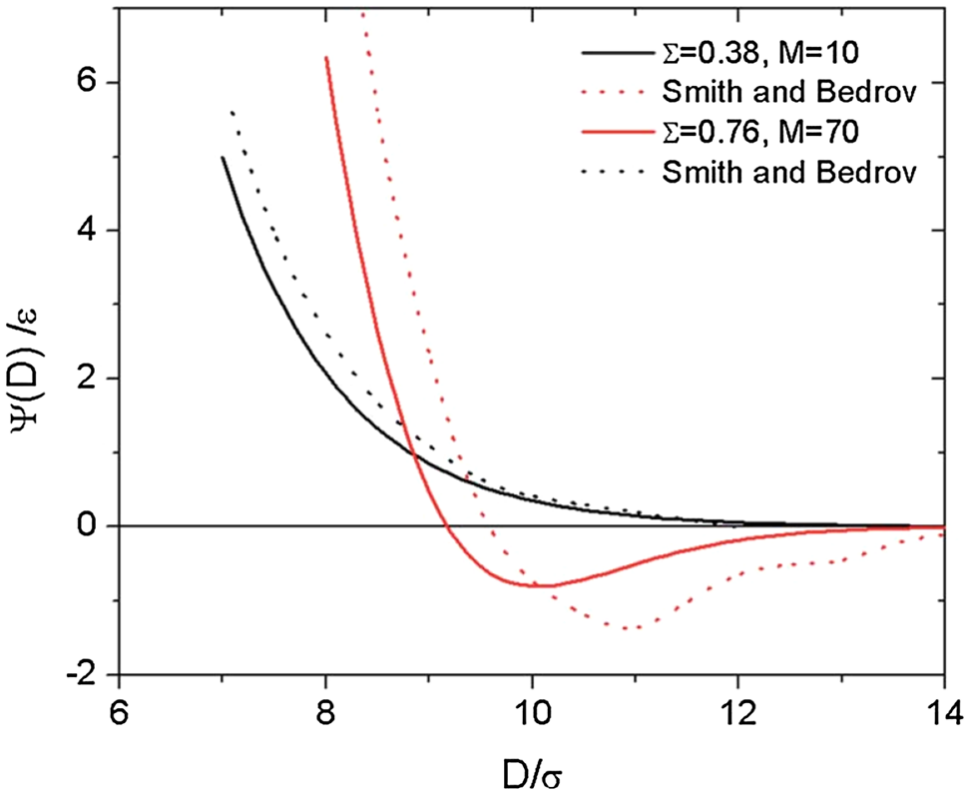
Potential of mean force between two grafted nanoparticles in two cases, $\Sigma = 0.38$ chains/$\sigma ^2$ and matrix chain length $M = 10$ shown in* solid black lines*; $\Sigma = 0.76$ chains$/\sigma ^2$ and $M=70$ shown in* solid red lines*. The* dotted lines* show the potential of mean force obtained from the corresponding cases of the work of Smith and Bedrov [274]. (Color figure online) (Reprinted from [266]—Published by The Royal Society of Chemistry.)

Meng et al. [266] used Molecular Dynamics simulations to delineate the separation dependent forces between two polymer-grafted nanoparticles in a homopolymer melt and the associated potential of mean force (PMF). The nanoparticle radius (=5 in units of the chain monomers) and grafted brush length (=10) were held constant, while the grafting density and the polymer matrix length were varied systematically in a series of simulations. At first, it was shown that simulations of a single nanoparticle did not reveal any signatures of the expected autophobic dewetting of the brush with increasing polymer matrix length (in agreement with Monte Carlo simulations of Vogiatzis and Theodorou [192]). In fact, density distributions of the matrix and grafted chains around a single nanoparticle appeared to only depend on the grafting density, but not on the matrix chain length. However, the calculated forces between two nanoparticles in a melt, presented in Fig. 27, showed that increasing the matrix chain length, *M*, from 10 to 70 causes the interparticle PMF to go from purely repulsive to attractive with a well depth of the order of $k_\text{B}T$ (with $k_\text{B}$ being the Boltzmann constant). It was speculated that these results were purely entropic in origin and arise from a competition between brush-brush repulsion and an attractive inter-particle interaction caused by matrix depletion from the inter-nanoparticle zone (i.e. an Asakura-Oosawa type inter-particle attraction). Figure 27 compares the PMF with the results from Smith and Bedrov’ simulations [274] of a similar coarse-grained system using the umbrella sampling method for an apparently identical chain length and coverage. The two studies are in qualitative agreement to each other, with the PMF of Meng et al. consistently shifted toward smaller separations.

**Fig. 28:**
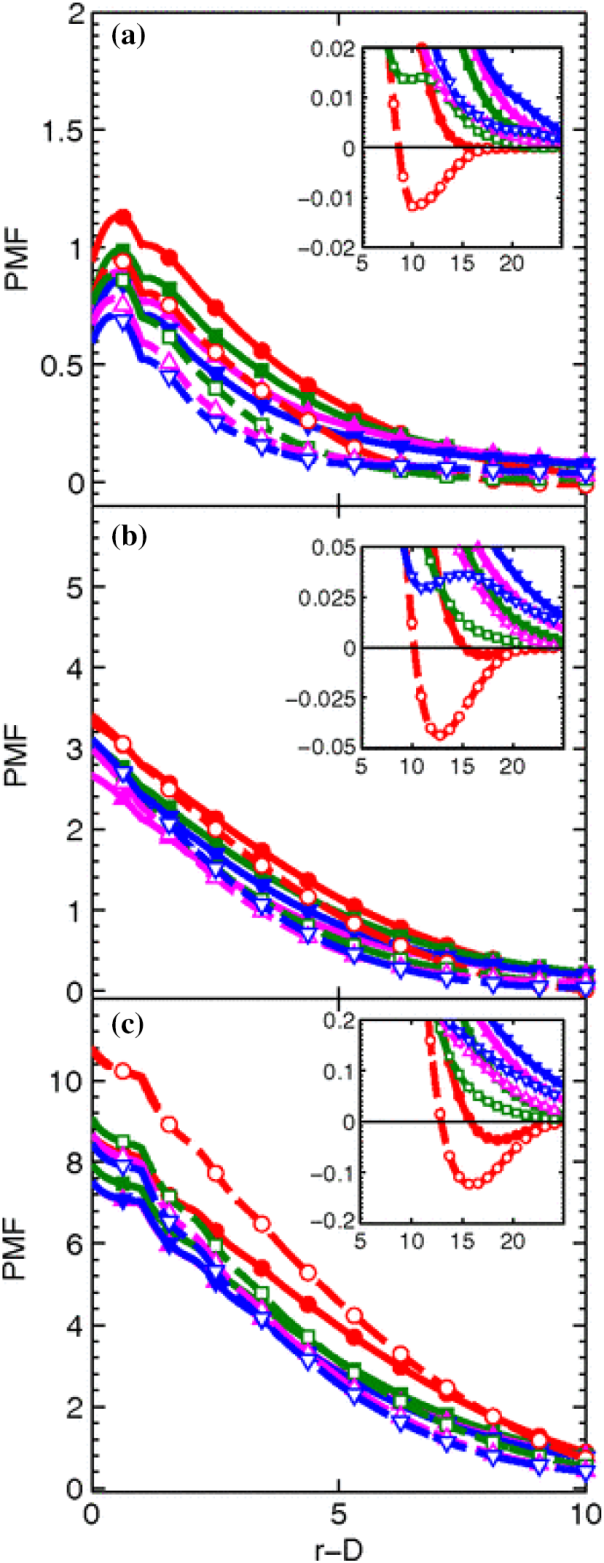
Potential of Mean Force (PMF) in units of $k_\text{B}T$ versus interparticle distance, $r-D$ (in units of monomer diameter, *d*), between grafted nanoparticles ($D= 5d$) at grafting denisties of** a** 0.1,** b** 0.25 and** c** 0.65 chains$/d^2$ and polydispersity indices 1.0 (*circles*), 1.5 (*squares*), 2.0 (*upward facing triangles*), and 2.5 (*downward facing triangles*) with average grafted chain length of 20 in a dense solution of monodisperse homopolymer matrix chains of lenght 10 (*solid symbols*) and 40 (*open symbols*). The* insets* have the same axis labels as the main plots. (Reprinted figure with permission from [275]. Copyright 2013 by the American Physical Society)

Martin et al. [275] presented an integrated theory and simulation study of polydisperse polymer grafted nanoparticles in a polymer matrix to demonstrate the effect of polydisperisty in graft length on the potential of mean force between the grafted particles. It is evident from Fig. 28 that increasing polydispersity in graft length reduces the strength of repulsion at contact and weakens the attractive well at intermediate interparticle distances, completely eliminating the latter at high polydispersity index. The reduction in contact repulsion was attributed to polydispersity relieving monomer crowding near the particle surface, especially at high grafting densities. The elimination of the midrange attractive well could be attributed to the longer grafts in the polydisperse graft length distribution that in turn introduced longer range steric repulsion and altered the wetting of the grafted layer by matrix chains. That work demonstrated that at high grafting densities, polydispersity in graft length can be used to stabilize dispersions of grafted nanoparticles in a polymer matrix at conditions where monodisperse brushes would cause aggregation.

## Rheology

### Polymer Entanglements

One of the fundamental concepts in the molecular description of structure—property relations of polymer melts is chain entanglement. As the molecular weight of the molecules in a polymer melt is increased, the spatial domain spanned by any given chain increasingly overlaps with those occupied by its neighbors. When macromolecules interpentrate, the term entanglements intends to describe the topological interactions resulting from the uncrossability of chains. The fact that two polymer chains cannot go though each other in the course of their motion changes their dynamical behavior dramatically, without altering their equilibrium properties. Entanglements play a key role in the viscoelastic properties of polymers, as evidenced, for example, by the emergence of a plateau region in measurements of the storage modulus as a function of frequency.

Molecular simulations have confirmed that the overall motion of the chains in a polymer melt is restricted to diffusion along their “primitive paths”, which represent the diffusive paths that linear chain molecules follow between their two ends as a result of topological constraints [149]. The advent of computational algorithms enabled direct observation of entanglements that arise in polymeric melts [276–278]. Anogiannakis et al. [38] have examined microscopically at what level topological constraints can be described as a collective entanglement effect, as in tube model theories, or as certain pairwise uncrossability interactions, as in slip-link models. They employed a novel methodology, which analyzes entanglement constraints into a complete set of pairwise interactions (links), characterized by a spectrum of confinement strengths. As a measure of the entanglement strength, these authors used the fraction of time for which the links are active. The confinement was found to be mainly imposed by the strongest links. The weak, trapped, uncrossability interactions cannot contribute to the low frequency modulus of an elastomer, or the plateau modulus of a melt.

#### Insight Obtained from Simulations

**Fig. 29:**
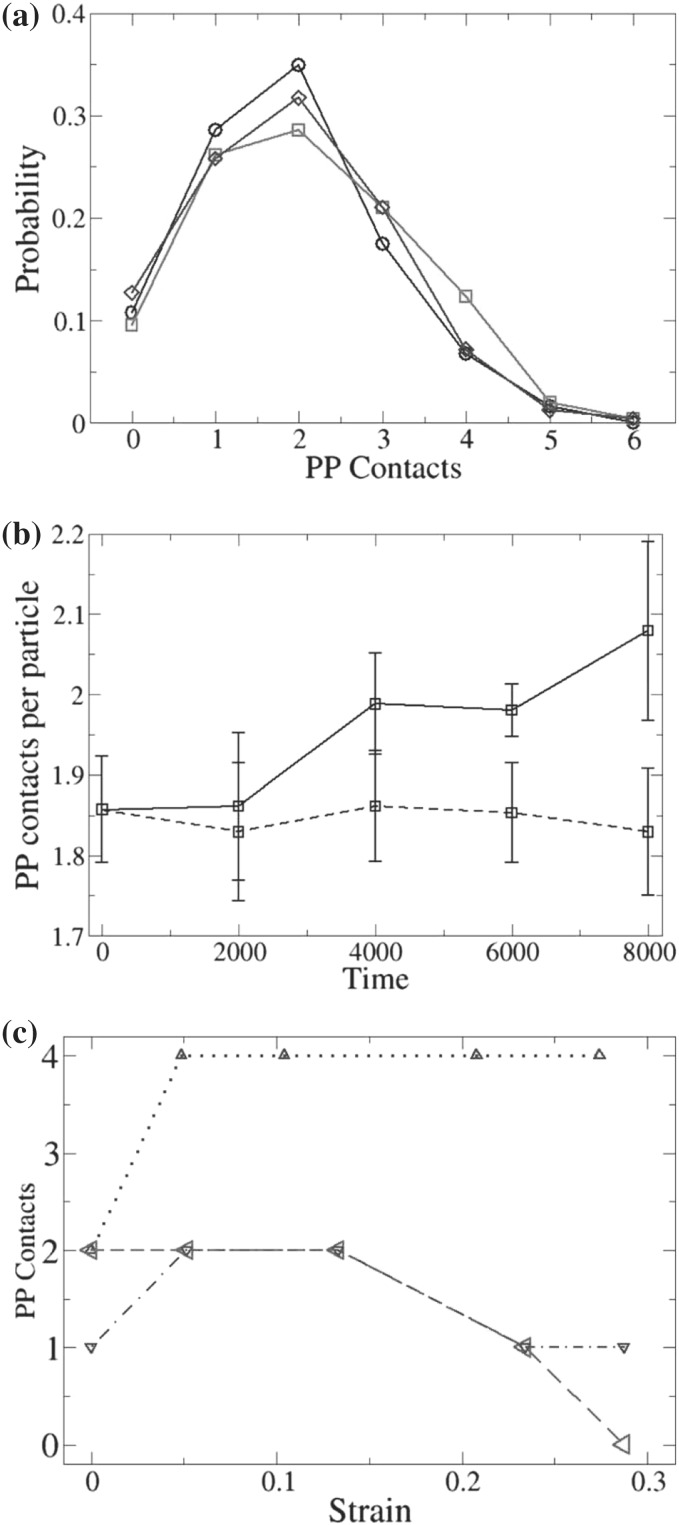
**a** Probability that a nanoparticle has a given number of contacts in the initial state (*open circles*), after compressive deformation (*open diamonds*), and after tensile deformation (*open squares*). The errors are approximately the size of the symbols.** b** The total number of primitive path contacts per nanoparticle as the system deforms in tension (*solid line*) or compression (*dashed line*).** c** Number of primitive path contacts plotted against the instantaneous strain for three chosen particles as the nanocomposite system deforms. The particle that exhibited the largest nonaffine displacements is represented by* left triangles* while the one with the smallest nonaffine displacements is plotted using up triangles. (Reprinted from [279], with the permission of AIP Publishing.)

Riggleman et al. [279] have carried out a detailed examination of entanglements in a nanocomposite glass. They have conducted Molecular Dynamics simulations of an ideal bead-spring polymer [149] nanocomposite model in which the nanoparticles were dispersed throughout the polymeric matrix. After equilibration in the melt state, all configurations were cooled below their glass transition temperature, where they were subsequently aged using MD for a short period. Finally, a simulation of the creep response of each sample was performed, where tensile and compressive stresses were applied to the glassy specimens. In order to reduce the chains to their primitive paths, these authors employed the CReTA algorithm of Tzoumanekas and Theodorou [278]. During the reduction process, the diameter of the particles is reduced to facilitate slippage of entangled chains past each other, up to the point that further decrease in the diameter of the particles no longer has an appreciable effect on primitive path statistics. The nanoparticles are necessarily frozen in space as the algorithm proceeds.

By examining all particles, one can calculate the distribution of the number of primitive path contacts per particle in the system, shown in Fig. 29(a). The majority of the particles trap at least one primitive path. The entanglements due to polymer chains crossing each other are expected to exhibit little (if any) change during deformation. The only mechanism for the entanglements to disappear is through chain ends slipping past an entanglement junction; such effects are anticipated to be minimal in the glassy state. However, Fig. 29(a) reveals appreciable changes in the distribution of the number of primitive path contacts per particle; the number of contacts per particle increases significantly upon deformation. Figure 29(b) shows how the average number of contacts per particle increases with time for both tensile and compressive deformations. An intriguing, overall physical picture emerges from the heterogenous nonaffine displacements and the particle-induced nucleation of entanglements (Fig. 29(b)). Figure 29(c) provides the number of entanglements for three particles. The particle that exhibits the largest nonaffine displacements begins with two primitive path contacts, and as the deformation proceeds loses its primitive path contacts. Since that particle was not hindered by any primitive paths, it was able to move throughout the system more easily. In contrast, the particle with the smallest nonaffine displacements (plotted using up triangles) experienced two or more primitive path contacts during the entire deformation. Those entanglements served to trap the particle and forced it to move along them, in an affine manner. Nanoparticles were found to serve as entanglement attractors, particularly at large deformations, altering the topological constraint network that arises in the composite material.

**Fig. 30:**
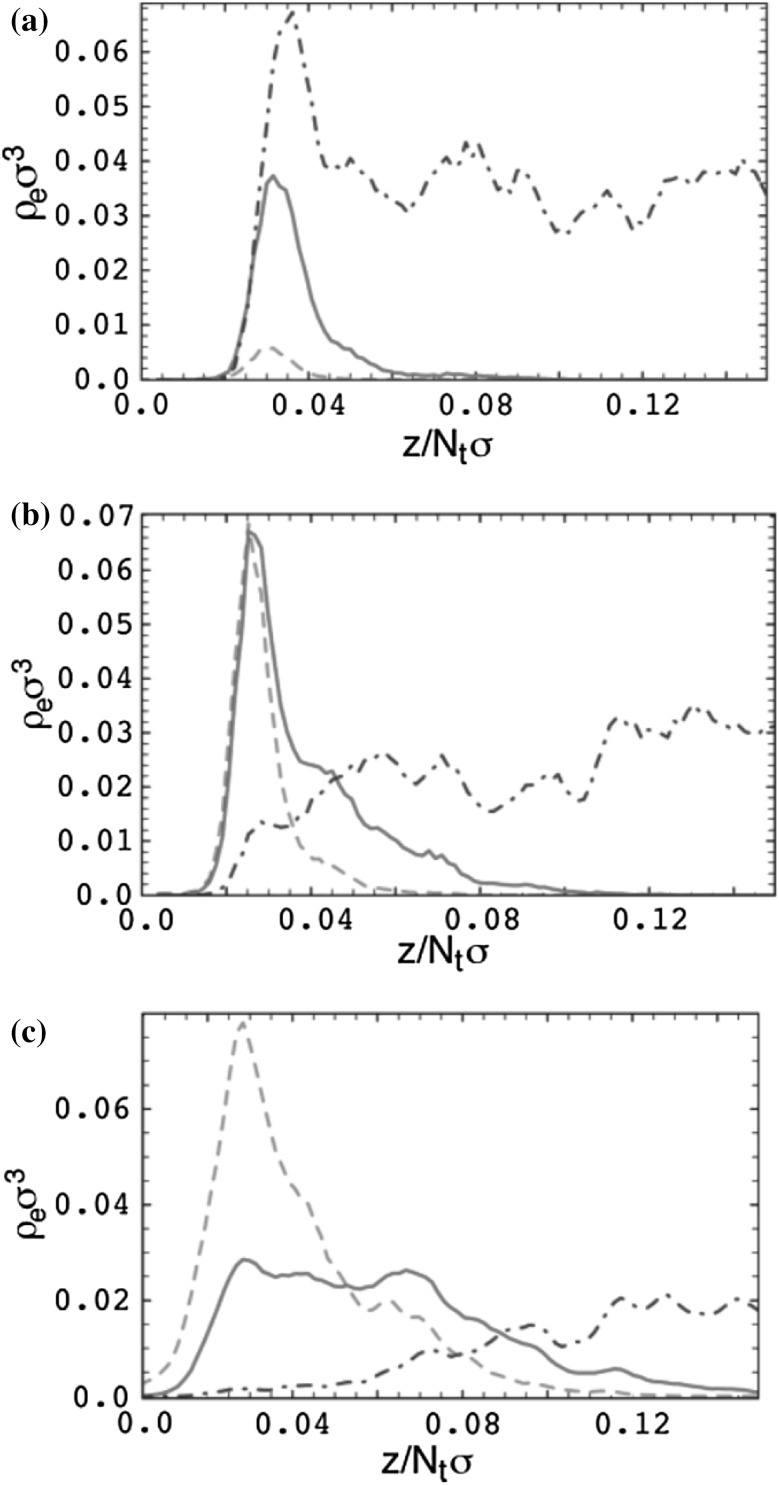
Brush-brush (*dashed*), brush-melt (*solid*) and melt-melt (*dash-dotted*) entanglement densities, $\rho _\text{e}^\text{bb}\left( z\right)$, $\rho _\text{e}^\text{bm}\left( z\right)$, and $\rho _\text{e}^\text{mm}\left( z\right)$ for three different grafting densities of** a** 0.008,** b** 0.03 and** c** 0.08 chains $\sigma ^{-2}$. (Reprinted with permission from Ref. [280]. Copyright (2007) American Chemical Society.)

Hoy and Grest [280] performed primitive path analysis [276] of polymer brushes embedded in long-chain melts. All simulations were for a coarse-grained model [149] in which monomers were represented by beads (of size $\sigma )$ connected by springs. The systems studied consisted of long grafted chains of length $N = 501$ beads, whereas the entanglement length in a melt is approximately $N_\text{e} = 70$ [281]. The polymeric matrix studied consisted of melt chains of length $P = 1000$ beads. As expected, the brush-brush entanglement density, $\rho _\text{e}^\text{bb}\left( z\right)$, increases rapidly with the grafting density for overlapping brushes. The brush-melt entanglement density, $\rho _\text{e}^\text{bm}\left( z\right)$, increases also with the grafting density, but even at low grafting densities there is considerable brush-melt entanglement. Moreover, there is clear crossover from dominance of brush-melt entanglements to brush-brush entanglements as coverage increases. Figure 30 depicts brush-brush, brush-melt and melt-melt entanglement densities for three different grafted densities 0.008, 0.03 and 0.07 (in units of $\sigma ^{-2}$). The peak of $\rho _\text{e}^\text{bm}\left( z\right)$ is always at $z \simeq 15 \sigma$, but the width of the first peak increases dramatically with increasing grafting density. At low *z*, the crossover between a preponderance of brush-melt entanglements and preponderance of brush- brush entanglements clearly occurs at $0.03 \sigma ^{-2}$ grafting density. At this coverage, the peaks of the brush-brush and brush-melt entanglement densities are of nearly equal height. For higher coverages, the peak of the brush-brush entanglement density is higher, the reverse of the situation for lower coverages. Summarizing, when surrounded by melt, the brushes entangle predominantly with the melt at low coverage and with themselves at high coverage. The peak of the brush-melt entanglement density is highest at an intermediate coverage, but the integrated areal brush-melt entanglement density continues to increase with coverage for the studied systems.

### Viscosity

#### Experimental Findings

Nanoparticles have been shown to influence mechanical properties, as well as transport properties, such as viscosity. Until recently, the commonly held opinion was that particle addition to liquids, including polymeric liquids, produces an increase in viscosity, as predicted by Einstein a century ago [282, 283]. However, it was recently found by Mackay and coworkers [208, 284, 285] that the viscosity of polystyrene melts blended with crosslinked polystyrene particles (and later also with fullerenes and other particles) decreases and scales with the change in free volume (due to introduction of athermal excluded volume regions in the melt) and not with the decrease in entanglement. Later, [285] fullerenes and magnetite particles were found to produce the same non-Einstein viscosity decrease effect.

Micron-sized spherical fillers increase the viscosity of a pure polymer melt from $\eta _\text{p}$ to a value of $\eta$ predicted by the Einstein–Batchelor law:

68 $$\begin{aligned} \frac{\eta }{\eta _\text{p}} = 1 + 2.5 \phi + 6.2 \phi ^2 \end{aligned}$$

where $\phi$ is the particle volume fraction [201, 219, 286]. However, for nanosized fillers, $\eta$ can be reduced or increased relative to the pure polymer [208, 284, 287–293]. While there have been extensive simulations on nanocomposites, a few of them have focused on the importance of nanoparticle addition on flow behavior [290, 294].

#### Insight Obtained from Simulations

**Fig. 31:**
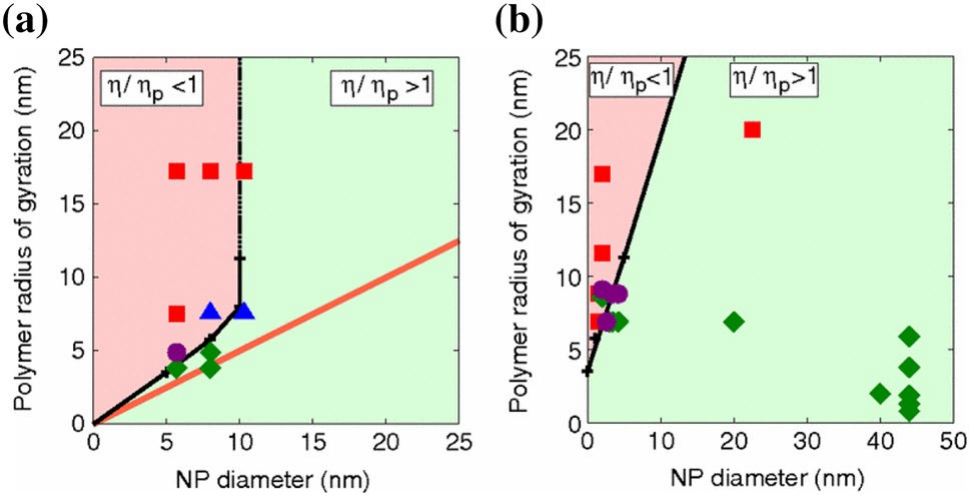
Viscosity of polymer nanocomposites as a function of the polymer radius of gyration and nanoparticle diameter.* Square * symbols correspond to $\eta / \eta _\text{p} < 1$, diamonds to $\eta / \eta _\text{p}> 1$, circles to $\eta / \eta _\text{p} \simeq 1$ at low nanoparticle loading, and triangles to the case where an initial increase of viscosity with nanoparticle loading is followed by a decrease.** a** Experimental data for athermal systems are from [208, 284]. Systems above the* solid orange line* should be miscible. The* black* “viscosity” line is extrapolated from the simulation findings.** b** Corresponding plot for dissimilar mixtures. Only the viscosity line is shown, Data are from [287–293] (Reprinted figure with permission from [196]. Copyright 2001 by the American Physical Society)

Kalathi et al. [196] employed nonequilibrium Molecular Dynamics simulations in order to find out whether the shear viscosity of a polymer melt can be significantly reduced when filled with small energetically neutral nanoparticles. That proved to be the case, apparently independently of the polymer’s chain length. Analogous to solvent molecules, small nanoparticles seem to act as plasticizers and reduce the viscosity of a polymer melt. Their simulations allowed them to organize the viscosity data of filled polymer melts as a function of the dimensions of the matrix chains and the particles. Figure 31(a) plots simulation data for athermal (with respect to the strength of polymer–particle interactions) polymer nanocomposite melts, which correspond to the experiments where the nanoparticles and the melts have the same chemical structure [208, 284], while chemically dissimilar mixtures are considered in Fig. 31(b) [287–293]. In Fig. 31(a), Kalathi et al. have also included the miscibility line from ref. [150] suggesting that the experiments correspond to miscible nanoparticle-polymer mixtures. The “viscosity” line drawn from the simulations separates regions where the nanocomposite’s viscosity is smaller from those where viscosity is larger than that of the pure melt. For short chains, the viscosity crossover occurs when the nanoparticle size is comparable to $R_\text{g}$. In contrast, the limited data for large $R_\text{g}$ suggest that the line is nearly vertical.

**Fig. 32:**
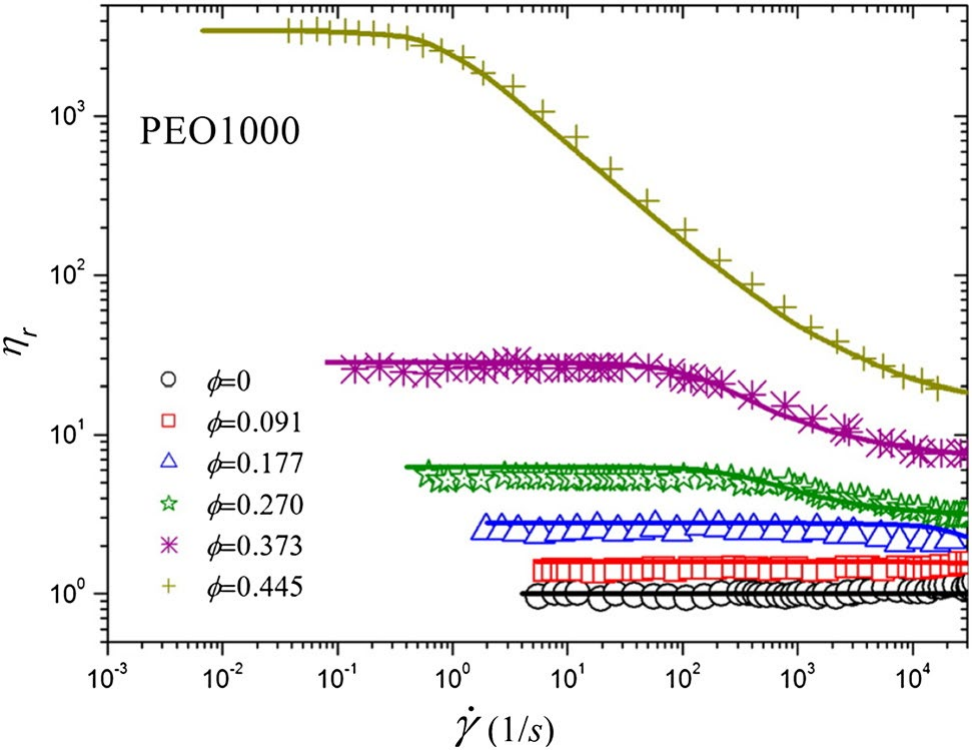
Comparison of the model predictions of Stephanou et al. [295] for the relative viscosity of a polymer nanocomposite as a function of nanoparticle volume fraction and imposed shear with the experimental measurements of Anderson and Zukoski [296]. (Reprinted with permission from Ref. [295]. Copyright (2014) American Chemical Society.)

Stephanou et al. [295] introduced a continuum model for polymer melts filled with nanoparticles capable of describing in a unified way their microstructure, phase behavior, and rheology in both the linear and nonlinear regimes. That model was based on the Hamiltonian formulation of transport phenomena for fluids with a complex microstructure with the final dynamical equations derived by means of a generalized (Poisson plus dissipative) bracket. The model describes the polymer nanocomposite melt at a mesoscopic level by using three fields (state variables): a vectorial (the momentum density) and two tensorial ones (the conformation tensor for polymer chains and the orientation tensor for nanoparticles). A key ingredient of the model is the expression for the Helmholtz energy, *A*, of the polymer nanocomposite. Beyond equilibrium, *A* contains additional terms that account for the coupling between microstructure and flow. In the absence of chain elasticity, the proposed evolution equations capture known models for the hydrodynamics of a Newtonian suspension of particles. Figure 32 presents the relative viscosity predicted by the model of Stephanou et al. for an unentangled PEO melt with molecular weight $M = 1000$ g/mol filled with silica nanoparticles of diameter $D = 43$ nm [296]. Due to the large nanoparticle volume fractions covered in the measurements (up to 50%), neither the Einstein equation nor the Einstein- Berthelot-Green one are applicable [296]. A better choice is the empirical equation proposed by Krieger and Dougherty [297] for dense Newtonian suspensions. It can also be observed that for $\phi \ge 0.27$ the data in Fig. 32 exhibit a plateau in the limit of infinitely high shear rates. At those shear rates, flow is so fast that thermal motion cannot destroy the imposed structure (fully aligned molecules) on polymer chains.

## Mechanical Properties

### Moduli of PNCs

Three different models [298] have been proposed by Einstein [282, 283], Eilers [299], and Guth [300] for estimating the enhancement of the shear modulus of composites incorporating spherical particles:

69 $$\begin{aligned} \frac{G}{G_\text{p}} = {\left\{ \begin{array}{ll} 1 + 2.5 \phi &{} \text{Einstein} \\ 1 + 2.5 \phi + 14.1 \phi ^2 &{} \text{Guth} \\ \left[ 1 + 1.25 \phi + \frac{1.25\phi }{1- 1.35\phi }\right] ^2 &{} \text{Eilers} \end{array}\right. } \end{aligned}$$

with $\phi$ being the volume fraction of particles dispersed and *G* and $G_p$ the shear moduli of the pure polymer and the composite, respectively. Einstein derived his model for small volume fractions of particles, where the enhancement in the shear modulus (or viscosity increase) can be estimated by a linear superposition of the shear distortions arising from individual particles; though this relationship was originally derived for shear viscosity of particle suspensions, it is also applicable to a host of other properties, including the shear modulus of composites. Later, Guth extended this model to higher $\phi$ by accounting for additional shear distortion arising from the interactions between the distortions arising from neighboring particles. Eilers made empirical corrections to Einstein model to account for the dramatic rise in the viscosity of suspensions observed when the volume fraction approaches the close-packing sphere density limit.

**Fig. 33:**
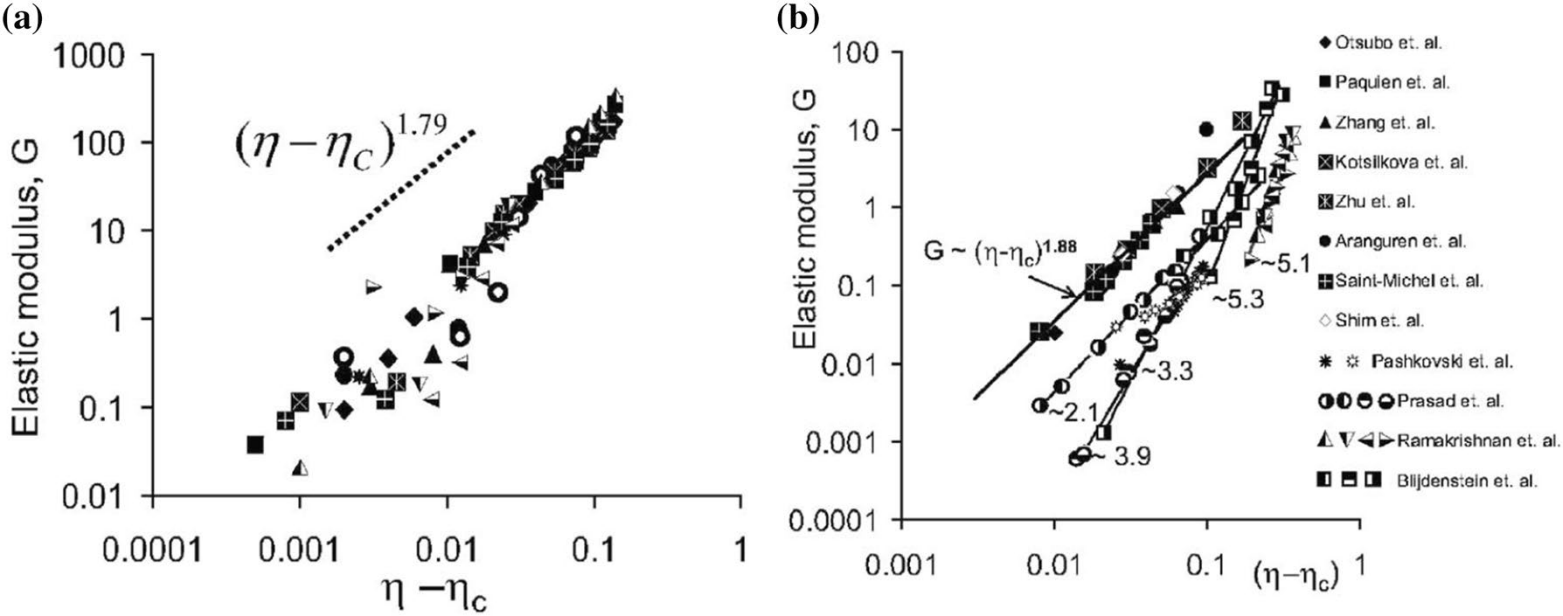
**a** Master curve for the scaled elastic modulus obtained from simulations as a function of the particle volume fraction $\eta - \eta _\text{c}.$ **b** Scaled elastic modulus for experimental polymer-particle systems. The sources of experimental data are listed in refs [301–311]. (Reprinted from [312], with the permission of AIP Publishing.)

Surve et al. [312] employed a combination of polymer mean field theory and Monte Carlo simulations to study the polymer-bridged gelation, clustering behavior, and elastic moduli of polymer-nanoparticle mixtures. Polymer self-consistent field theory was first numerically implemented in order to quantify both the polymer induced interaction potentials and the conformational statistics of polymer chains between two spherical particles. Subsequently, the formation and structure of polymer-bridged nanoparticle gels were examined using Monte Carlo simulations. These authors used the number distribution of bridges, obtained from their simulations, to quantify the elastic properties of the polymer nanocomposites in the postgel regime. Similar to classical network theories, they assumed that the only contribution to the elastic response of the system comes from the backbone of the percolated network and that the “sol” fraction and the dangling ends of the network do not impact elasticity to the system. They defined the backbone of the percolated network as the percolated cluster, excluding the dangling tails and dangling loops, that can be identified as the largest biconnected component of a percolated cluster. Since, for the case of bridging induced percolation, the interparticle bridges served as the stress bearing bonds between the particles, the enhancement in the elastic modulus was assumed to be proportional to the number of such bridges, at a given volume fraction of particles. Figure 33(a) displays the elastic moduli scaled by a constant factor as a function of particle volume fraction, expressed as $\eta - \eta _\text{c}$, with $\eta$ and $\eta _\text{c}$ being the volume fraction and the percolation volume fraction of the particles, respectively. As observed from the figure, the elastic moduli follow a universal power law scaling, $G \propto \left( \eta - \eta _\text{c}\right) ^{\nu _\eta }$, with $\nu _\eta \simeq 1.79.$ If energetic contributions to elasticity are taken into account, higher elastic exponents appear from $\nu _\eta {\sim}2.1$ to 3.8 depending on the relative influence of stretching entropy and bending energy [313, 314] (Fig. 33(b)).

**Fig. 34:**
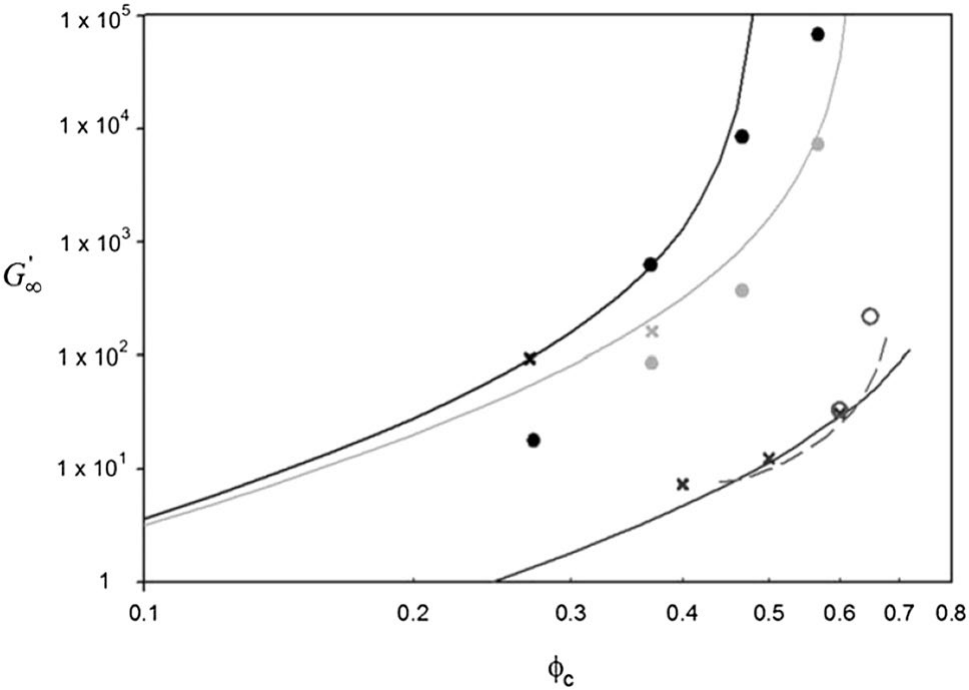
Correspondence between the measured and the predicted moduli, $G_\infty ^\prime$, of radius $R_\text{n} \simeq 100 \; \text{nm}$ silica particles in 2 kg mol$^{-1}$ PDMS (*filled circles*), $R \simeq 100$ nm particles in 13 kg mol$^{-1}$ PDMS (*filled light gray circles*) and $R \simeq 600$ nm particles in 8 kg mol$^{-1}$ PDMS (*open dark gray circles*). Modulus predictions are also shown: MC simulations (matching colored “x” symbols), the analytical Zwanzig-Mountain relation (matching colored* solid lines*), and Hall equation for close packed hard spheres (*dark gray dashed line*). (Reprinted from [315]—Published by The Royal Society of Chemistry.)

McEwan et al. [315] predicted the storage modulus by employing a Zwanzig–Mountain relation and Monte Carlo simulations. In parallel, these authors measured the modulus from rheology experiments on samples well characterized with ultra-small angle X-ray scattering. These authors connected particle microstructure to the storage modulus at infinite frequency, $G_{\infty }^\prime$, through the Zwanzig-Mountain equation for isotropic molecular fluids [316]:

70 $$\begin{aligned} G_{\infty }^{\prime } = {\frac{k_\text{B}T}{R_{{\text{n}}}^{3}}} \left( \frac{3\phi _{\text{c}}}{4 \pi } + \frac{3 \phi _{\text{c}}^{2}}{40 \pi } \int _0^{\infty } g\left( r\right) \frac{\text{d}}{\text{d}r}\left[ r^{4} \frac{\mathop {\text{U}}\limits _ \cdot \left( r\right) /\left( k_{\text{B}}T\right) }{\text{d}r}\right] \text{d}r\right) \end{aligned}$$

where the first term within the parentheses represents an ideal contribution to the modulus due to the presence of the particles and the second term accounts for the contribution due to their interaction and microstructure. The radial distribution of nanoparticles, $g\left( r\right)$, was obtained from Monte Carlo simulations of the particles obeying a Mewis-Russel [317] potential for polymer-grafted spheres. Their results are presented in Fig. 34. Silica particles of radii $R \simeq 100$ nm and 600 nm were synthesized, grafted with hydroxyl-terminated polydimethylosiloxane (PDMS) chains and finally dispersed in PDMS matrices at volume fractions, $\phi _{\text{c}},$ranging from 0.02 to 0.65. The experimental measurements are presented alongside the theoretical results in Fig. 34. It can be clearly seen that the storage modulus increases upon the addition of particles, following an almost universal scaling with the volume fraction of the particles. In all cases, the Zwanzing-Mountain predictions are very close to the experiments and to the predictions of the Hall equation of state for solids [318] (at high volume fractions where the materials behave in a solid-like manner).

**Fig. 35:**
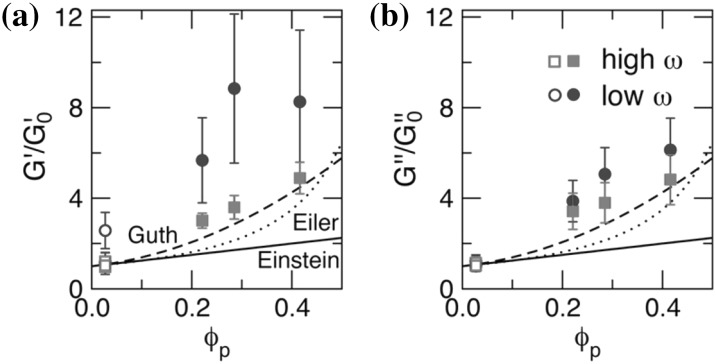
Ratio of storage (**a**) and loss modulus (**b**) of the three bare- and the three grafted-nanoparticle systems to that of pure polymer plotted as a function of the effective nanoparticle volume fraction. The moduli ratios obtained from simulations at low and high frequencies are shown as* blue circles *and* red squares*, respectively.* Open symbols* represent bare nanoparticles, and* filled symbols* represent grafted nanoparticles. The* black solid*,* dotted*, and* dashed lines* represent predictions from the models of Einstein [282, 283], Eilers [299], and Guth [300], respectively. (Color figure online) (Reprinted with permission from Ref. [319]. Copyright (2015) American Chemical Society.)

To provide insights into how polymer-grafted nanoparticles (NPs) enhance the viscoelastic properties of polymers, Hattemer and Arya [319] computed the frequency-dependent storage and loss moduli of coarse-grained models of polymer nanocomposites by employing Molecular Dynamics simulations. Figures 35(a) and (b) present the computed $G^\prime / G^\prime _0$ and $G^{\prime \prime }/G^{\prime \prime }_0$ ratios plotted against $\phi$ for the six polymer nanocomposites containing bare and grafted nanoparticles at low- and high-frequency regimes along with the the moduli predicted by using the theoretical models of Einstein, Eilers and Guth. It is evident that both $G^\prime / G^\prime _0$ and $G^{\prime \prime }/G^{\prime \prime }_0$ increase with increasing volume fraction, consistent with the trend obtained from the strain distortion models, though the different models differ somewhat from each other. Moreover, the moduli ratio computed from simulations at high frequencies are of comparable magnitude to those predicted by the models, especially the model proposed by Guth, whereas the ratios computed at low frequencies tend to exceed all model predictions. At low frequencies, the computed $G^\prime / G^\prime _0$ ratios are more strongly affected compared to $G^{\prime \prime }/G^{\prime \prime }_0$, which is consistent with the expectation that $G^\prime$ is more strongly affected by changes in the relaxation times of the polymer chains (quadratic dependence with the Rouse time) as compared to $G^{\prime \prime }$ (linear dependence).

### Local Moduli

**Fig. 36:**
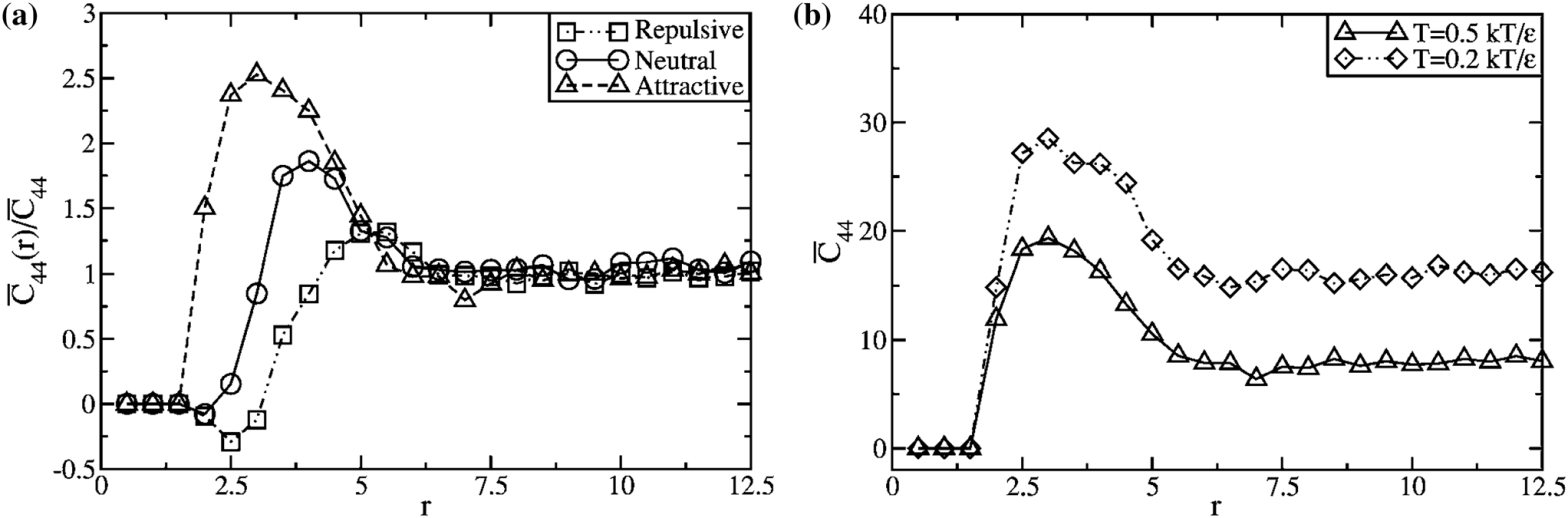
**a** Distribution of local $\bar{C}_{44}$ with respect to the distance *r* from the center of a nanoparticle for three different types of interaction considered at temperature $T = 0.5$.** b** Distribution of the local $\bar{C}_{44}$ with respect to the distance *r* from the center of the nanoparticle for the attractive particle in the melt and glass regime. (Reprinted figure with permission from [320]. Copyright 2005 by the American Physical Society)

It is now generally accepted that a nanoparticle will perturb the conformation of the polymer around it. However, the question still remains open whether such conformational changes are directly responsible for the mechanical behavior of the polymer, i.e. whether there are any reinforcement or weakening effects of a polymer by a nanometer-sized particles, whether such effects are localized, and if so, what is the extent and the magnitude of that localization. Papakonstantopoulos et al. [320, 321] have developed a formalism and applied it to calculate the local mechanical properties of a nanocomposite system in detail. Their coarse-grained, bead-spring Monte Carlo simulations revealed that a glassy layer is formed in the vicinity of the attractive filler, contributing to the increased stiffness of the composite material. Following Yoshimoto et al. [322], the local mechanical properties of the system were determined by discretizing the simulation box into small cubic elements and measuring the internal stress fluctuations within each cubic subdomain [323].

Figure 36(a) shows the local shear modulus as a function of the distance from the surface of the filler. An increase of the local $\bar{C}_{44}$ is observed for the attractive systems in the vicinity of the particle. This pronounced increase may be indicative of the existence of a glassy layer around the particles, even at temperatures above the glass transition temperature ($T/T_\text{g} = 1.16$), which was hypothesized by Berriot et al. [324]. The results of the neutral and repulsive system are more intriguing. The nanoparticle is surrounded by a region of negative modulus which is followed by a second region of higher than the bulk modulus. Figure 36(b) shows the local shear modulus as a function of temperature for the attractive particle. It can be seen that, as the temperature decreases, the shear modulus of the solid-like layer around the particle increases. The thickness of that glassy layer, which is comparable to the radius of gyration of the polymer, also increases. In all cases, far from the particle, the shear modulus decays to the value corresponding to the pure polymer at the given temperature, as expected. Summarizing, it seems that, even above the glass transition temperature, nanoparticles induce the formation of a solid-like layer, whose existence has been invoked to explain experimentally observed increases of the storage modulus in nanocomposites.

### Deformation Simulations

**Fig. 37:**
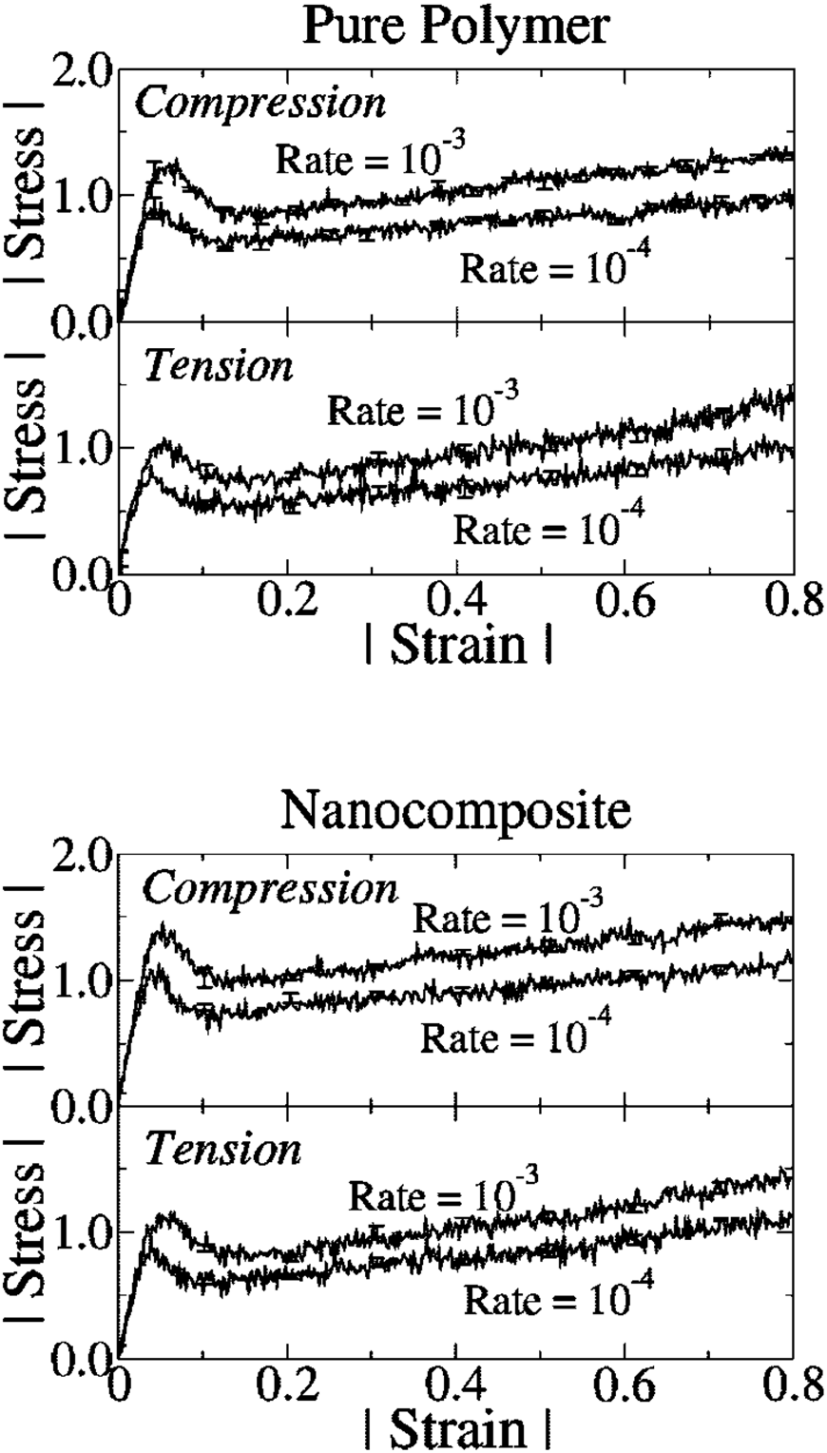
Stress-strain curves for both the pure polymer and the nanocomposite at two true strain rates in both tension and compression. The strain rates are indicated in each figure.* Error bars* are indicative. (Reprinted with permission from Ref. [325]. Copyright (2009) American Chemical Society.)

Riggleman et al. [325] have examined the response of a polymer and a polymer nanocomposite glass to creep and constant strain rate deformations using Monte Carlo and Molecular Dynamics simulations. These authors found that nanoparticles stiffened the polymer glass, as evidenced by an increase in the initial slope of the stress-strain curve and a suppression of the creep response. Figure 37 shows the stress-strain curves obtained by Riggleman et al. [325] for both the neat and the nanocomposite polymer for both tension and compression at two different strain rates. All curves exhibit similar features: it can be discerned an initial elastic response followed by yield and strain softening when the strain $\varepsilon \simeq 0.05$. For strains beyond $\varepsilon \simeq 0.10$ the stress rises again as strain hardening begins. The Young’s modulus, *E*, was obtained by fitting the linear part of the elastic response ($\varepsilon \le 0.02$) $\sigma = E \varepsilon$. Both under tension and compression, the nanocomposite system was found to be stiffer. Moreover, these authors reported that constant strain rate and constant stress deformations had different effect on the material’s position on its energy landscape, in a way that neither the stress nor the strain rate were uniquely indicative of the relaxation times in the material [326].

**Fig. 38:**
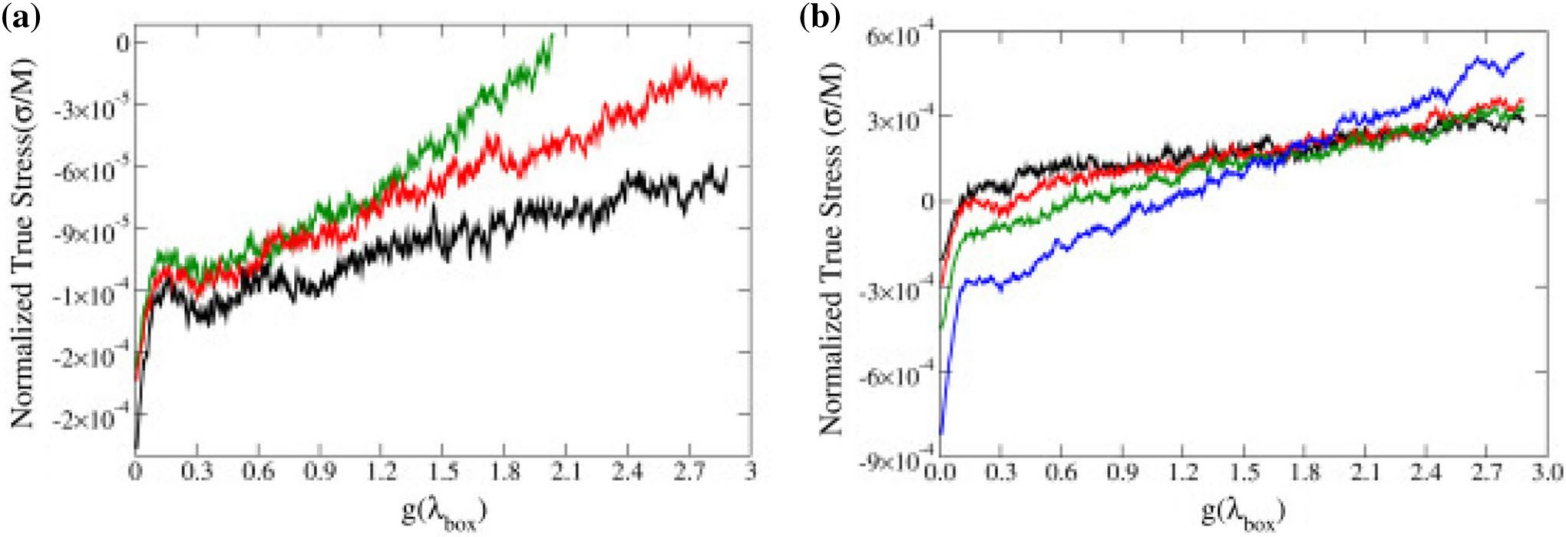
Different contributions to the stress tensor plotted against $g\left( \lambda \right)$.** a** Stress contributions from the non-bonded interaction between particles and the grafted chains for the systems with grafting density 0.2, and $R_\text{n} = 1.5$ (*black*), 3.0 (*red*), and 4.5 (*green*).** b** Stress contributions from the non-bonded interactions between the monomers of the grafted chains for the systems with $R_\text{n} = 3$ and grafting densities 0.05 (*black*), 0.1 (*red*), 0.2 (*green*), and 0.4 (*blue*). All the stress values are normalized by the total number of grafted chains, *M*. (Color figure online) (Reprinted from [327] with permission from Elsevier.)

Chao and Riggleman [327] studied the effect of nanoparticle curvature and grafting density on the mechanical properties of polymer nanocomposites. In their study, they developed a coarse-grained model of a polymer glass containing grafted nanoparticles and examined the resulting effects on the elastic constants, strain hardening modulus, as well as the mobility of the polymer segments during deformation. They found that the elastic constants and yield properties were enhanced nearly uniformly for all nanocomposite systems studied, while the strain hardening modulus depended weakly on the grafted density and the nanoparticle size. Figure 38 shows the mechanical response of the systems studied under compressive deformation at a constant rate, where the measured stress is plotted against the ideal rubber elasticity factor, $g\left( \lambda \right) = 1/\lambda - \lambda ^2$, with $\lambda$ being the macroscopic stretch imposed on the specimens. Early in the deformation, the polymer nanocomposites exhibit an elastic response ($g\left( \lambda \right) < 0.15$) followed by yielding and strain softening. Finally, at larger stretches ($g\left( \lambda \right)> 0.3$), the polymer glasses enter the strain hardening regime, and the stress resumes an increasing trend as strain continues to grow. These authors decomposed the stress calculated in their simulations into its components. The normalized contribution from the non-bonded interactions between the nanoparticles and the grafted chains is presented in Fig. 38(a). It can be seen that the interaction between the nanoparticle and the grafted chains increases with the particle size, and its effect is only observed in the strain hardening region. This finding is coherent with the expected depletion of the matrix from particle surfaces with increasing particle size. Similarly, the stress contributions from the non-bonded interactions among the grafted chains is presented in Fig. 38(b). For low grafting densities (0.05 and 0.1) the non-bonded interaction between beads belonging to grafted chains does not contribute significantly to the stress increase during strain hardening. However, for higher grafting densities (0.2 and 0.4), the non-bonded interactions between the grafted chains contribute significantly to strain hardening. In contrast to the obvious dependence of the non-bonded component of the stress tensor to the grafting density and particle radius, the bonded component of the stress tensor did not exhibit significant changes in behavior across the various nanocomposite systems investigated by Chao and Riggleman [327].

**Fig. 39:**
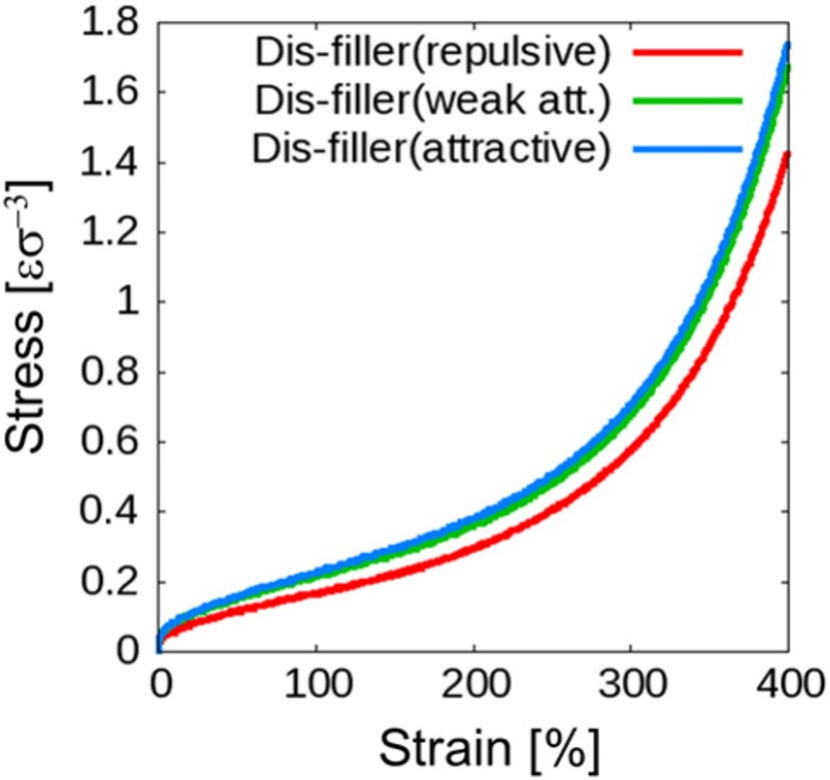
Stress–strain relations of cross-linked polymer nanocomposite networks with dispersed nanoparticles. Values of stress depend on the strength of polymer–nanoparticle interactions. In the high strain region, a rapid increase of the stress can be seen due to extended subchains of the crosslinked network. (Reprinted with permission from Ref. [328]. Copyright (2016) American Chemical Society.)

Hagita et al. [328] performed coarse-grained Molecular Dynamics simulations of nanocomposite rubbers with spherical nanoparticles on the basis of the Kremer-Grest [149] model. Figure 39 shows the stress–strain relations of a small mesh cross-linked polymer network for three nanoparticle–polymer interactions (repulsive, slightly attractive and attractive). There are clear differences between the repulsive and the attractive cases. However, both cases with attractive nanoparticle–polymer interactions seem to behave similarly, probably due to the few contacts existing between the nanoparticles. Thus, the effect of the exact interaction strength on the stress–strain relations is minor. When the nanoparticles are trapped and fixed in a cross-linked polymer network, the number of contacts between them is expected to increase for a larger elongation ratio due to the compression in the directions perpendicular to the elongation axis. These authors [328] have also calculated the two-dimensional scattering patterns of nanoparticles during the elongation of the network. For strain levels >50% they observed a spot pattern in the structure factor and a two-point bar pattern in the scattering intensity.

## Concluding Remarks

We have presented a detailed, result-driven, review of research to address the fundamental problem of PNCs by implementing computer simulations at different levels of description. It is unfeasible through the use of a single simulation technique to capture all the relevant physics of the problem. On the one hand, fully atomistic Molecular Dynamics (MD) can account for the chemical interaction between nanoparticles and the polymer matrix. However, due to the computational demands of atomistic MD, the polymer matrix has to be comprised of oligomers rather than entangled polymers. On the other hand, coarse-grained methods can provide us with an understanding of the underlying phenomena. However, due to the lack of explicit chemical information, coarse-grained methods should be carefully parameterized based on findings of more detailed simulations. In any case, even the wide spectrum of molecular simulation methods developed till now cannot fully capture the macroscopic behavior of PNCs. Coupling molecular simulations to continuum calculations is the way to achieve this [329–331].

## Footnotes

1 If the kinetic energy can be separated into a sum of terms, each of them being quadratic in only one momentum component, the average kinetic energy per degree of freedom is $1/2 k_\text{B} T$, which is a special case of the *equipartition theorem* [92].

2 The following problem was posed by Georges-Louis Leclerc, Comte de Buffon: given a needle of length *l* dropped on a floor striped with parallel lines *t* units apart, to find the probability that the needle will land such that it crosses a line. (The answer being $\left( 2 l\right) /\left( t \pi \right)$.)
